# Supplementary material for: Inherent instability of simple DNA repeats shapes an evolutionarily stable distribution of repeat lengths
Source: Nat Commun. 2025 Dec 9;17:93. doi: 10.1038/s41467-025-66725-5 (PMC12769509; doi:10.1038/s41467-025-66725-5)
Supplement: Supplementary file 1 — Supplementary Information [file 41467_2025_66725_MOESM1_ESM.pdf]

Inherent instability of simple DNA repeats shapes an evolutionarily stable distribution of repeat lengths

Supplementary Information

Supplementary Figures

1 *Motif labeling and distributions of repeat tract lengths (DRLs).* . . . 3
2 *DRL normalization for comparison across genomes of varying lengths.* . . . 4
3 *Distributions of repeat tract length per motif across phylogenies.* . . . 5
4 *Nucleotides gained or lost per indel event.* . . . 7
5 *Instability rate estimates.* . . . 8
6 *Construction of informative priors.* . . . 10
7 *Inference results for three-parameter multiplier-coupled power-law model of repeat instability rates.* . . . 12
8 *Posterior probability distributions for interpolated length-dependent rates.* . . . 13
9 *Posterior probability distributions under logarithm-based parameterization.* . . . 14
10 *Inference results for four-parameter decoupled power-law model of repeat instability rates.* . . . 15
11 *Late-time DRLs from computational model across parameter space.* . . . 18
12 *Constraints from genome size and equilibration time.* . . . 19
13 *Demonstrations of robustness of inference procedure to simplifying approximations.* . . . 20
14 *Comparison between computational model results and numerical solutions to steady state equations for $m = 4$ for parameter values with of constant $\tau_\epsilon + \tau_\kappa = 3.5$.* . . . 21
15 *Comparison between computational model results and numerical solutions to steady state equations for $m = 1$.* . . . 22
16 *Comparison between computational model results and numerical solutions to steady state equations for $m = 16.3$.* . . . 23
17 *Accuracy of analytic approximation for distribution falloff in the $\Delta\tau \gg 1$ regime.* . . . 24
18 *Computational model results for the net flux per mutation type.* . . . 25
19 *Computational model results for directional flux per mutation type* . . . 26
20 *Computational model results for collective non-normalized fluxes showing the relevance of local transitions, fission, and fusion.* . . . 27
21 *Pure power-law parameterization and the onset length of repeat instability.* . . . 28
22 *Assessing the onset length of repeat instability for longer motifs.* . . . 29
23 *Parameterization nesting procedure.* . . . 30

Supplementary Note

1 Analytic modeling of the repeat length distribution in steady state 31
1.1 Mutational processes affecting repeats . . . 31
1.2 Summary of the three-parameter multiplier-coupled model (for long repeats) . . . 33
1.3 Mutational processes as transitions in length space (local vs. nonlocal) . . . 33

2 Finite difference equation for repeat length changes 34
2.1 Changes in repeat length due to expansion, contraction, and insertion . . . 34
2.2 Changes in repeat length due to substitutions . . . 35
2.3 Finite difference equation . . . 36

3 Steady-state dynamics in the short repeat length regime 36

3.1 Geometric solution to the substitution-only difference equation . . . 37
3.2 Interactions between short and long repeats are largely restricted to boundary effects . . . 38

4 Steady-state dynamics for asymptotically long repeat lengths 38
4.1 Local contributions to the change in P_L . . . 38
4.1.1 Competition between first-order local effects . . . 40
4.1.2 Second-order corrections to the local behavior and diffusive dynamics . . . 41
4.2 Repeat fission as a nonlocal contribution to changes in length . . . 42
4.2.1 Substitution-based fission . . . 42
4.2.2 Insertion-based fission . . . 42
4.3 Repeat fusion under random sampling of the length distribution . . . 43
4.4 Steady-state condition for long repeat dynamics . . . 44
4.5 Decomposition of parameter space into dynamical regimes . . . 45
4.5.1 Asymptotic length dependence of local transition rates and $\Delta\tau$ . . . 45
4.5.2 Relative strength of substitution- and insertion-driven fission and L_{fis} . . . 46
4.5.3 Distinguishable dynamical regimes . . . 46
4.6 Unstable dynamics in the asymptotically expansion-biased regime $\Delta\tau \leq 0$ . . . 47
4.7 Stable dynamics in the asymptotically contraction-biased regime $\Delta\tau > 0$ . . . 48
4.7.1 Strong asymptotic contraction bias . . . 49
4.7.2 Strictly local approximation for strong asymptotic contraction bias $\Delta\tau \gg 1$ . . . 49
4.8 Intermediate asymptotic contraction bias . . . 50
4.9 Weak asymptotic contraction bias . . . 50
4.10 Obtaining numerical solutions to the steady state dynamics for $\Delta\tau < 0$ . . . 51

5 Comparison between numerical solutions and computationally modeled distributions 51
5.0.1 Comparisons of dynamical approximations and steady-state distributions . . . 52

6 Estimation of L^* and L_{fis} from the Bayesian posterior distribution 53

Supplementary References 54

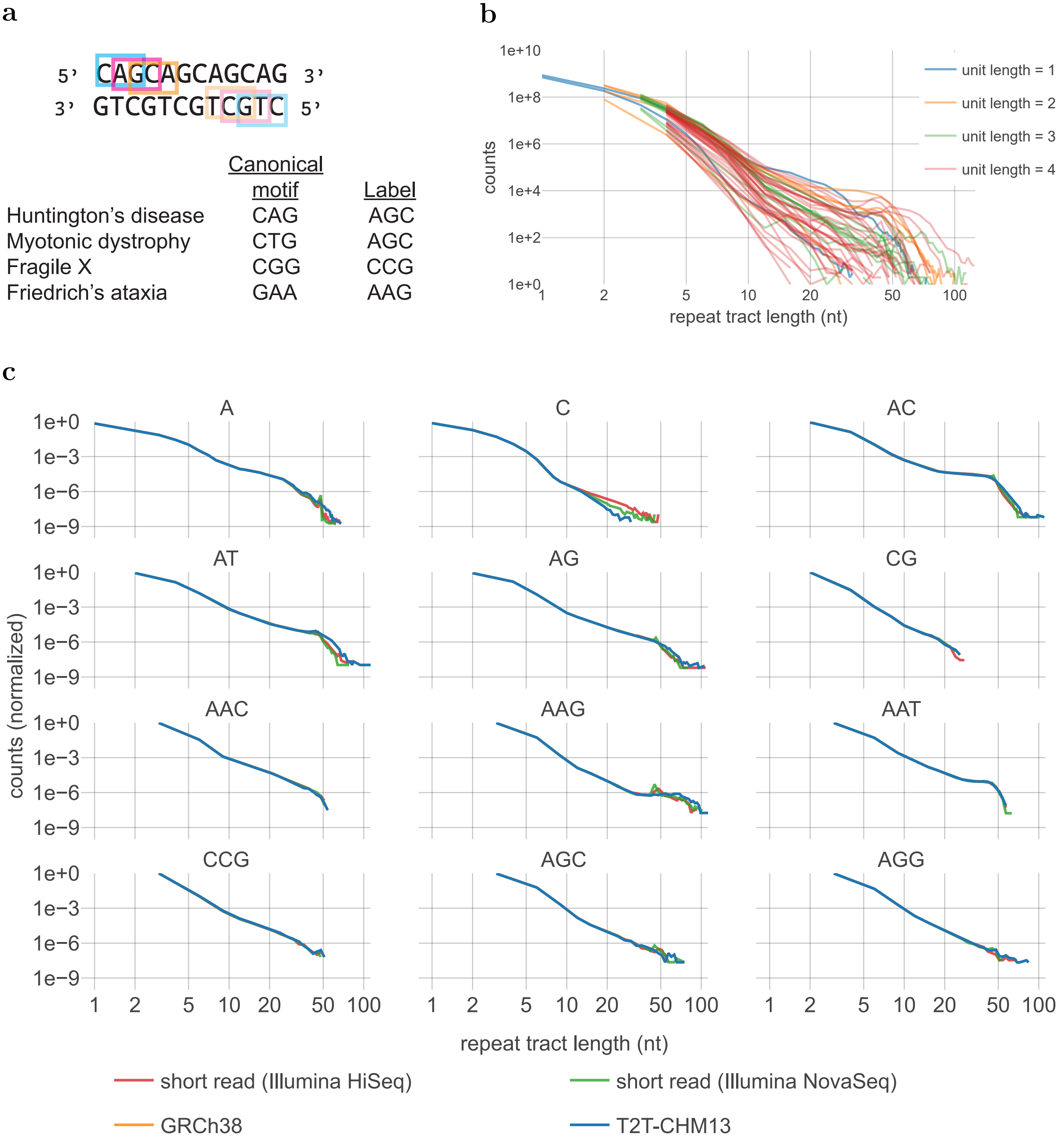

Supplementary Figure 1: *Motif labeling and distributions of repeat tract lengths (DRLs).* **(a)** Motif labels describe estimates pooled across equivalent cyclical permutations and their reverse complements (example shown for 'AGC'), using the alphabetically-first label. Labels of well-studied repeat expansion disorders are provided as examples.**(b)** DRLs in human T2T genome for all distinct motifs with unit lengths 1–4 nt. Motifs of the same unit length are shown in the same color. DRLs vary by motif but share similar qualitative features. **(c)** DRLs in four distinct human genome assemblies with different sequencing technologies. Normalization is required to account for differences in total sequence length (see **Supplementary Figure 2**). T2T-CHM13 was assembled using multiple long read technologies, while other assemblies employed shorter read technologies. Results shown for all motifs of unit length 1–3 (labeling described in **(a)**). Read length does not appear to affect the accurate counting of repeats of sub-disease lengths (with the exception of long mono-C repeats in one example short-read genome).

a

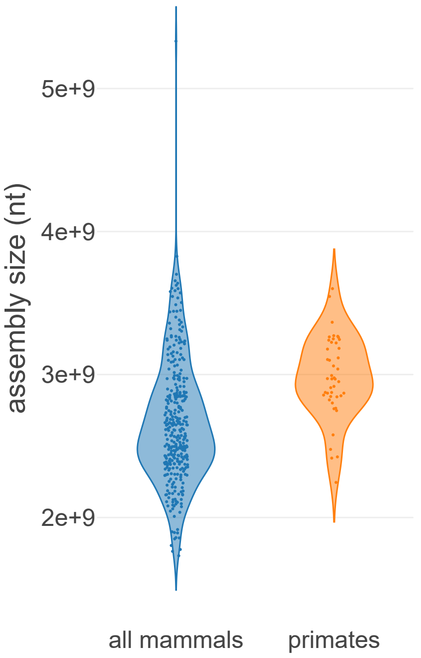

b

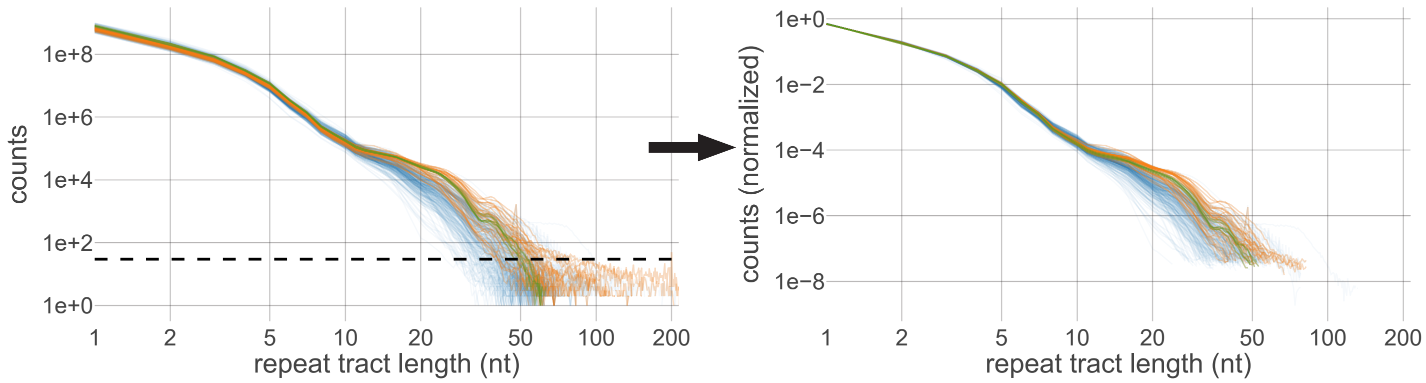

Supplementary Figure 2: *DRL normalization for comparison across genomes of varying lengths.* **(a)** Differing assembly size (i.e., total sequence length in each FASTA file, rather than complete in vivo genome size) for $n = 315$ mammalian genomes (blue), and the subset of $n = 37$ primate genomes, including humans (orange). **(b)** Comparisons between genomes necessarily require normalization due to expected differences in total number of repeats. Schematic example of DRL normalization for mono-A repeats (used to produce **Figure 1b**). Each line represents DRL for an individual species (hominids in green, all other primates in orange, all other mammals in blue). Left plot shows raw (non-normalized) DRLs with a dashed line at 30 counts; DRLs are truncated (at the shortest bin with counts < 30; see **Methods**) to remove highly stochastic bins prior to normalization (right plot).

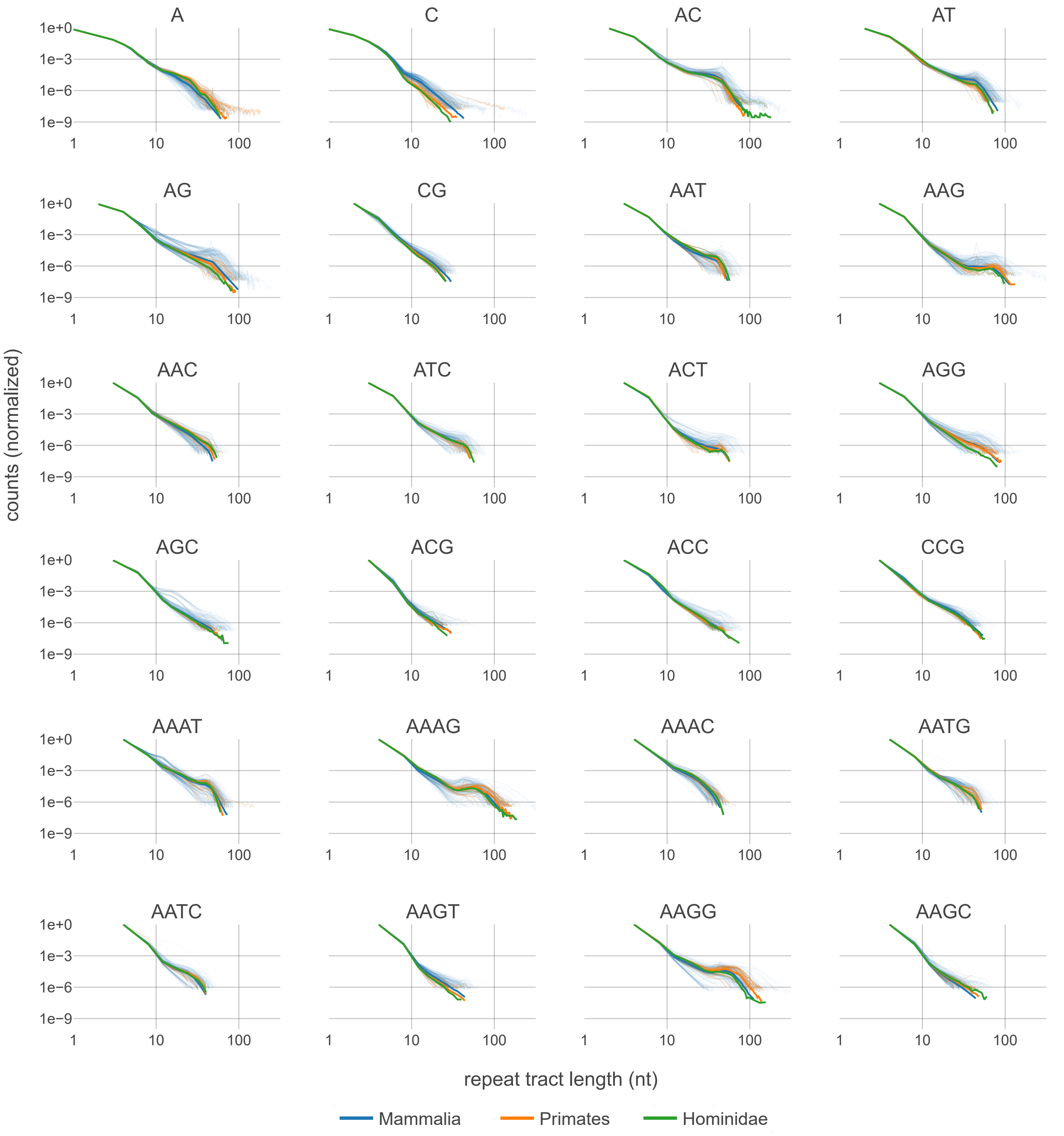

Supplementary Figure 3: *Distributions of repeat tract length per motif across phylogenies.* Normalized DRLs for all motifs of unit length 1–4 in mammals (blue; n = 315), primates (orange; n = 37) and hominids (green; n = 6). Motif labels include equivalent cyclical permutations and their reverse complements (see **Supplementary Figure 1a**). Counts are necessarily normalized to account for different genome lengths (see **Supplementary Figure 2**, **Methods**). Thin transparent lines show individual species; DRLs are truncated at the shortest bin containing counts < 30 prior to normalization (see **Supplementary Figure 2**). Solid line indicates median values per length bin for each phylogeny. Median calculations within phylogenies are inclusive (e.g., primates are included as a subset mammals). Overlapping medians between primates and hominids suggests long-term stability of the DRLs, while individual mammalian species display variability. (*continued on next page*)

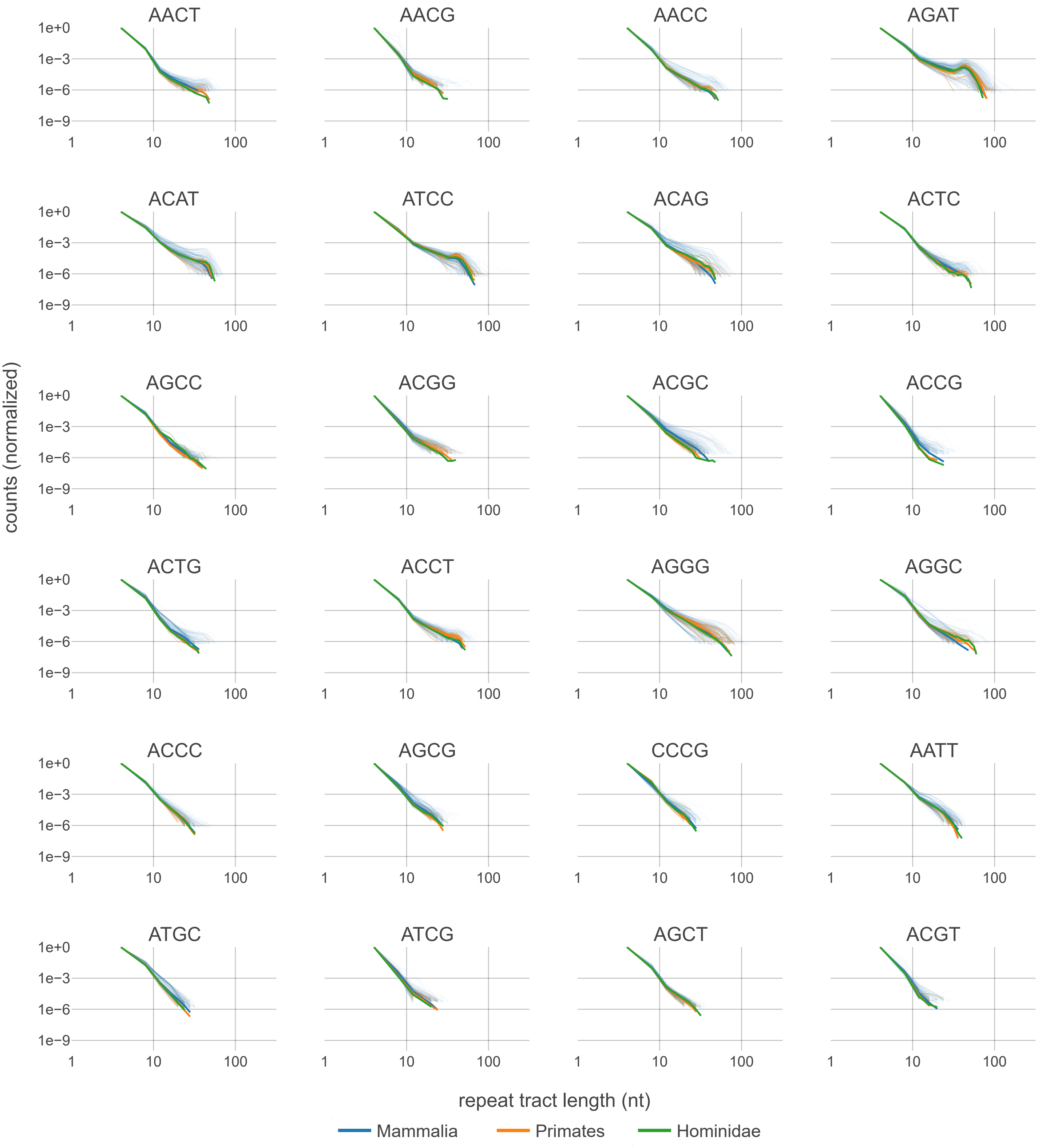

Supplementary Figure 3 (continued): *Distributions of repeat tract length per motif across phylogenies.*

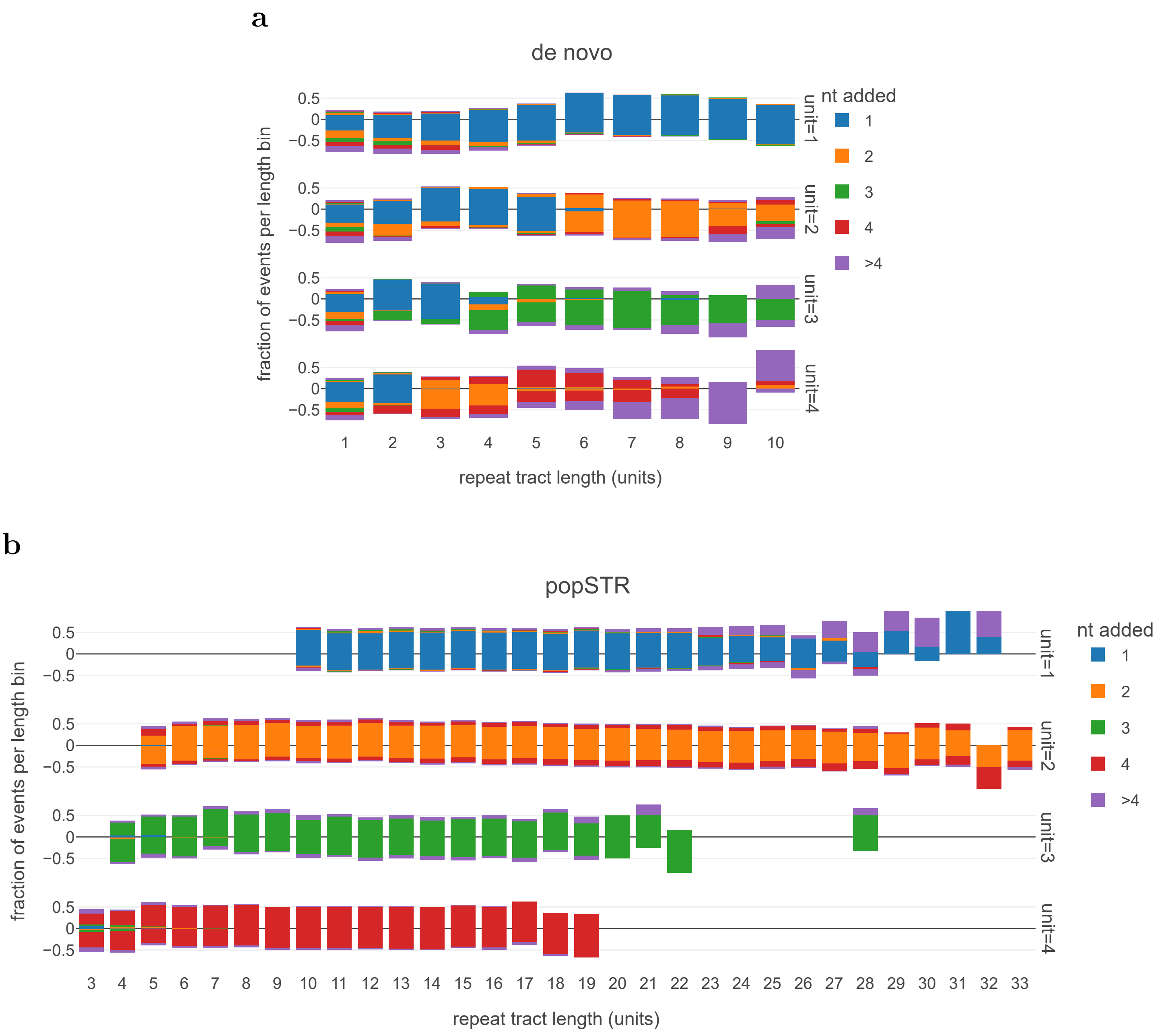

Supplementary Figure 4: *Nucleotides gained or lost per indel event.* De novo indels measured from two different data sources: **(a)** pooled trio sequences (n = 9387) and **(b)** popSTR estimates (n = 6084). Row labels indicate the motif unit length. X-axis displays the tract length of the repeat in number of units. Y-axis displays the fraction of events per length bin, according to the number of nucleotides gained or lost per event (indicated by color). Y-axis sign indicates insertion (positive) or deletion (negative); overall shift up or down indicates bias. Most events involve the gain or loss of a single complete unit, independent of total repeat length; both partial-unit and multiple-unit changes are less frequent. A length threshold is apparent (see **Figure 3a**), above which single unit indels characterize repeat instability. Note that indel calling in the de novo dataset loses accuracy at longer tract lengths; likewise, the popSTR database is not sufficiently populated below 10 nt and above ~ 30 nt.

a

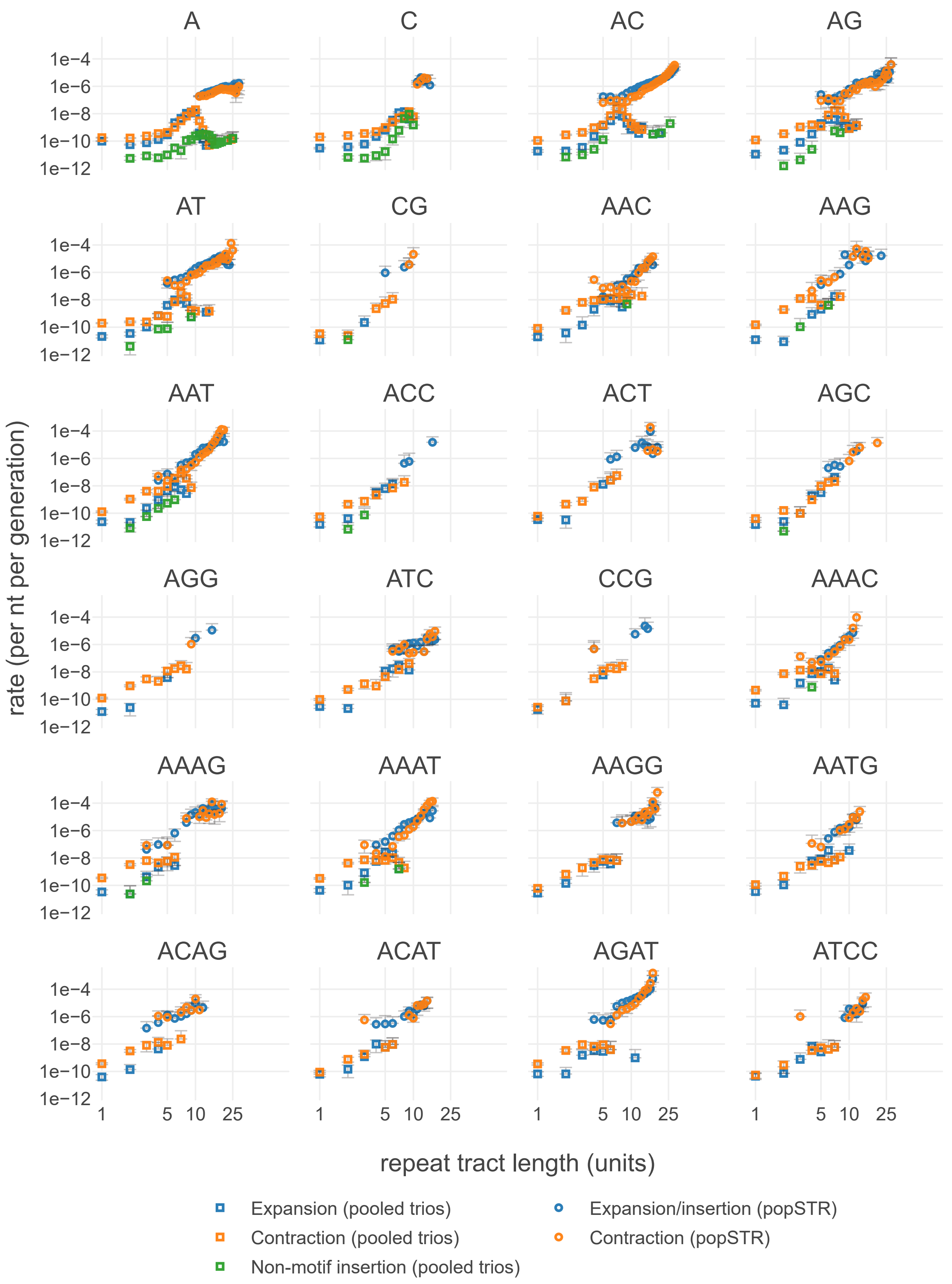

Supplementary Figure 5: *Instability rate estimates.* **(a)** Rate estimates from pooled-trio (n = 9387) and popSTR (n = 6084) datasets for expansion, contraction and non-motif insertions for all repeat motifs of unit lengths 1–4 nt. Statistical error bars show 95% CI assuming Poisson mutation counts. Motif labels include equivalent cyclical permutations and their reverse complements (see **Supplementary Figure 1a**). Complete estimates shown (no points omitted due to systematic errors and/or low sequencing quality).

(*continued on next page*)

b

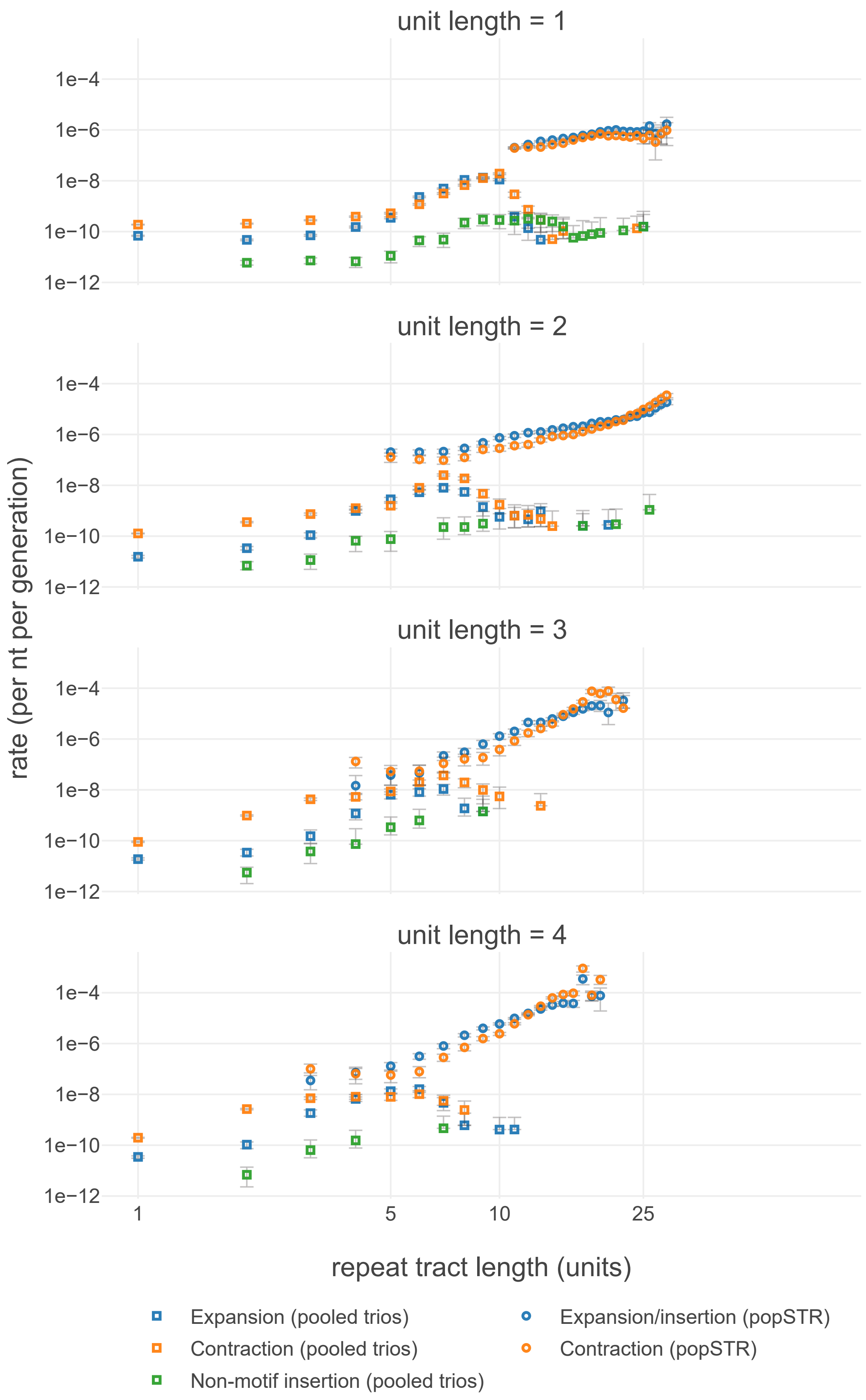

Supplementary Figure 5 (continued): *Instability rate estimates.* **(b)** Rate estimates from de novo and popSTR datasets for expansion, contraction and non-motif insertions, pooled by motif unit length. Statistical error bars show 95% CI assuming Poisson mutation counts. No points omitted due to systematic errors and/or low sequencing quality.

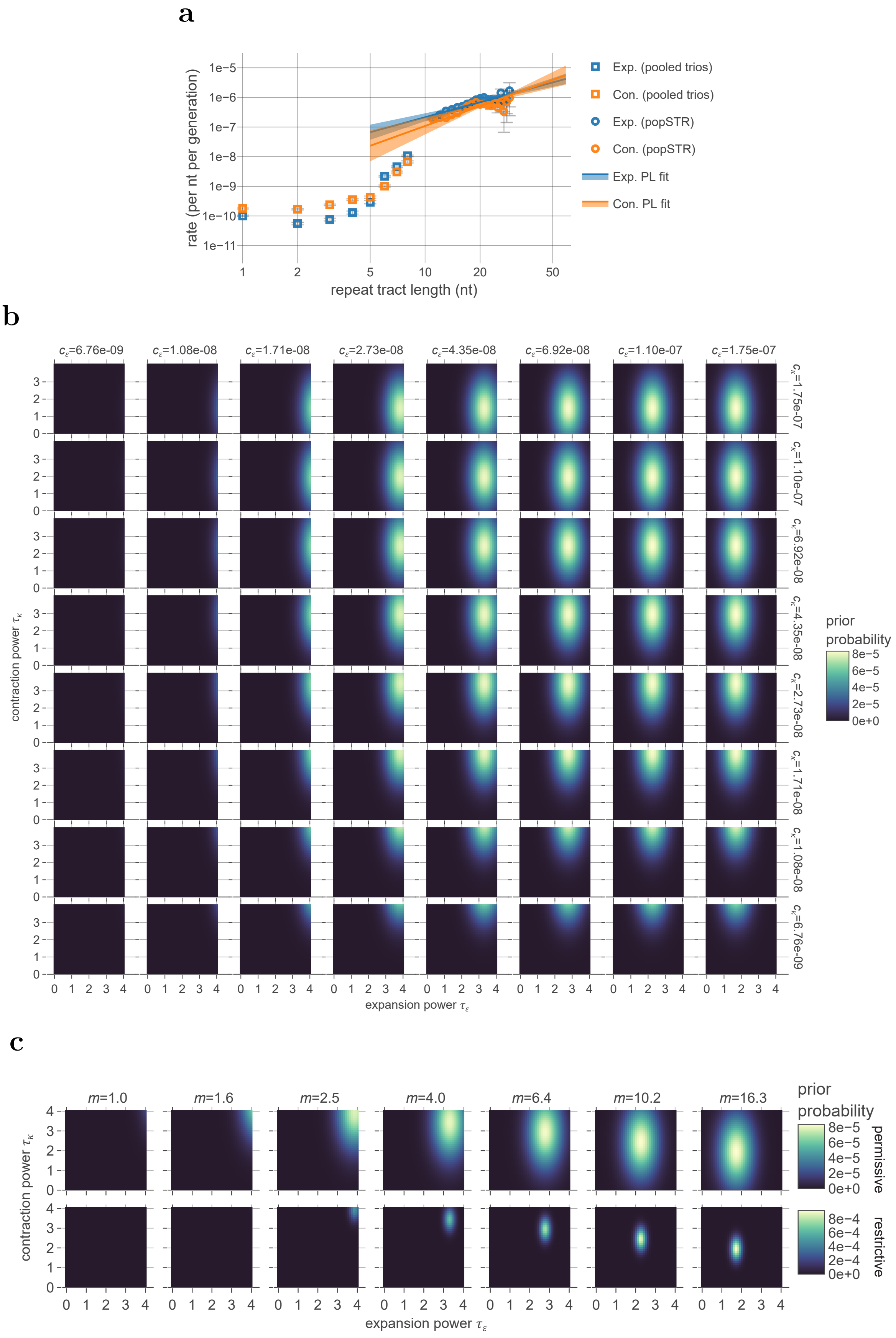

Supplementary Figure 6: *Construction of informative priors.* **(a)** Rate estimates from pooled-trio (n = 9387) and popSTR (n = 6084) datasets for expansion, contraction and non-motif insertions for mono-A repeats, as shown in **Figure 3b**. Lines show best-fit to popSTR expansion (plus insertion) and contraction rate estimates under a linear model in log-log space (i.e. power law; see **Methods**). Transparency approximates 95% confidence region for each linear fit. (*continued on next page*)

Supplementary Figure 6 (continued): *Construction of informative priors.* **(b)** Bayesian informative prior for the four-parameter decoupled power law model (parameterization in **Table 1**). Parameters $(C_\epsilon, C_\kappa, \tau_\epsilon, \tau_\kappa)$ shown as (columns, rows, x-axes, y-axes). Prior was constructed as a multivariate Gaussian probability distribution with mean parameters corresponding to best fit in **(a)**; covariance matrix estimated from confidence band after artificially inflating variances and covariances (1000× for permissive; 100× for restrictive, not shown). See **Methods** for further detail. **(c)** Informative priors for three-parameter multiplier-coupled power law model. Due to nested parameterizations (see **Supplementary Figure 23**), these priors were defined as appropriately re-normalized slices of the four dimensional prior (**top**: permissive; **bottom**: restrictive).

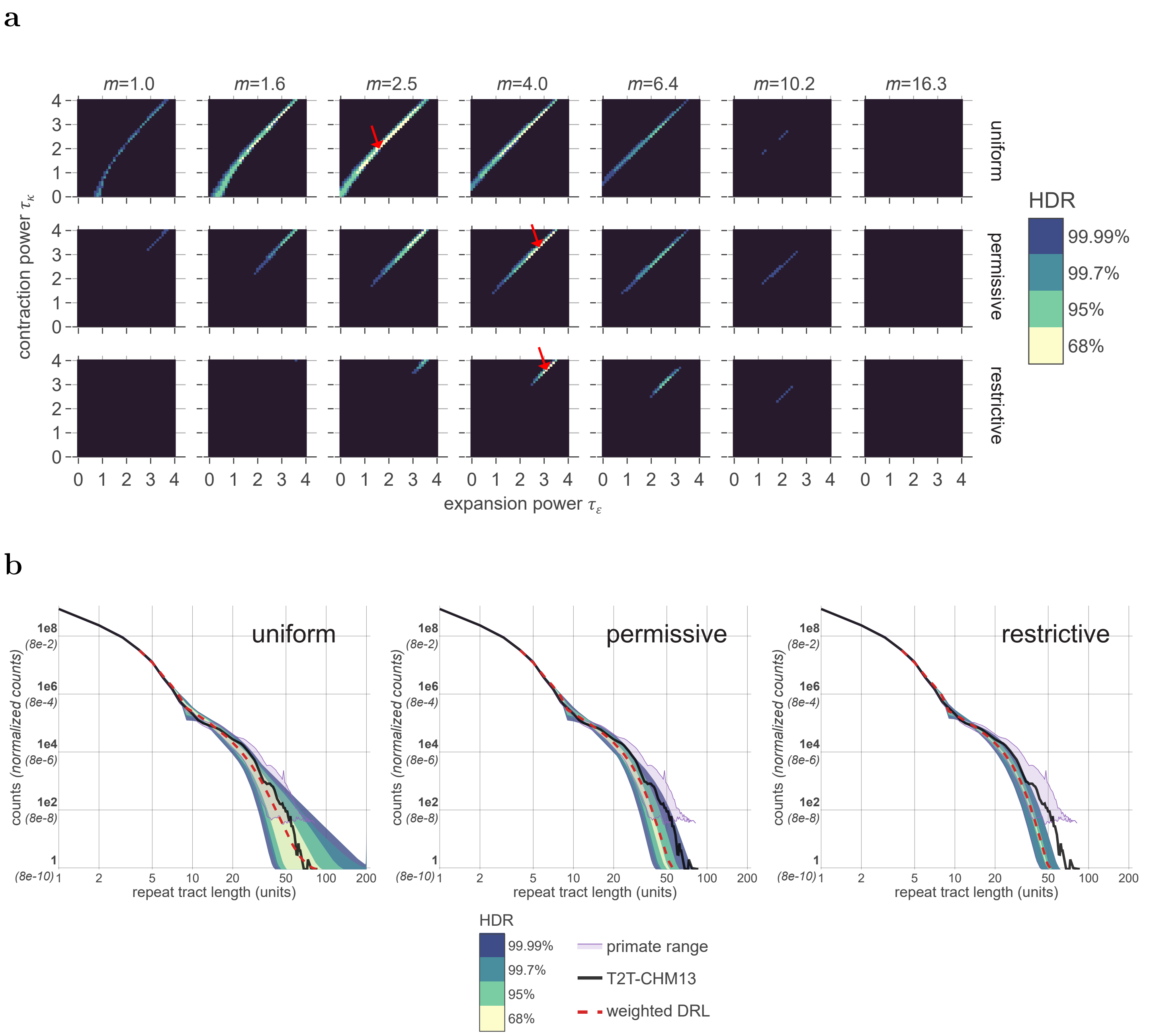

Supplementary Figure 7: *Inference results for three-parameter multiplier-coupled power-law model of repeat instability rates.* **(a)** Plots display Bayesian posterior probabilities after applying uninformative (top row), permissive (middle row) and restrictive (bottom row) informative priors (see **Supplementary Figure 6**). Length-dependent instability rates defined by parameters $(m, \tau_\epsilon, \tau_\kappa)$ shown as columns, x-axes and y-axes, respectively. Color bar indicates highest density range (HDR) of the posterior for specified total probabilities; black region sums to 0.01% of the probability. Red arrows show maximum posterior for each prior. **(b)** Comparison of empirical DRLs to various inference results under specified priors. Counts for all DRLs are necessarily normalized for comparison (see **Methods**); y-axis indicates normalized fractions (parentheses) and counts rescaled to match the number of repeats in the T2T genome (bold labels, black curve). Dashed line represents posterior-weighted DRL (an average of all DRLs weighted by the posterior probability for each parameter combination; see **Methods**). Colored ranges represent the minimum and maximum counts at each length bin across all parameters in the specified HDR (with corresponding colors in **(a)**). Purple region shows the min-max range generated from n = 34 non-human primate genomes (after removing the two most-diverged DRLs and appropriately normalizing; see **Supplementary Figure 2**, **Methods**).

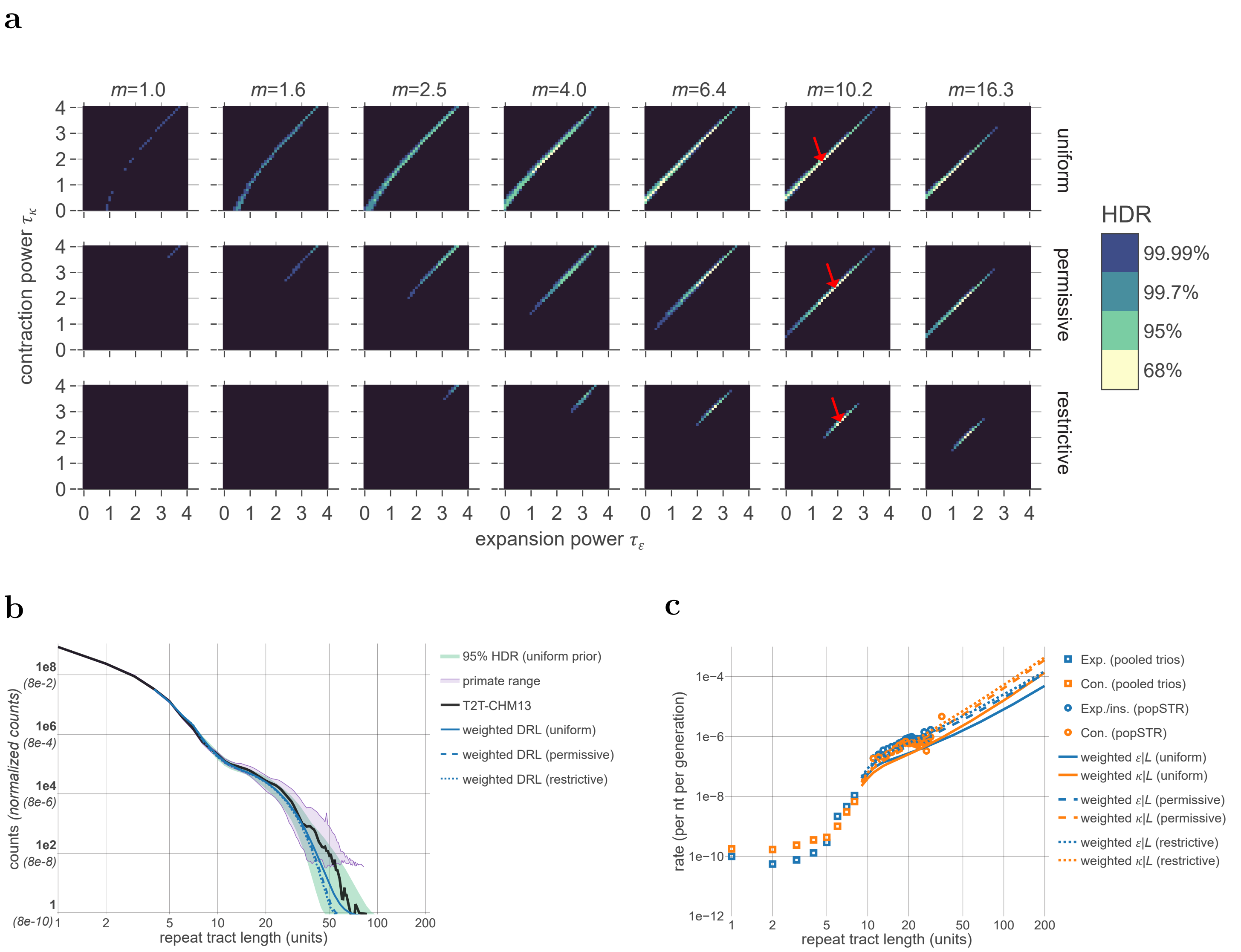

Supplementary Figure 8: *Posterior probability distributions for interpolated length-dependent rates.* **(a)** Inference results for three-parameter multiplier-based power-law model of repeat instability rates after interpolating between lengths 8 and 13 nt (see **Methods**). Plots display Bayesian posterior probabilities after applying uninformative (top row), permissive (middle row) and restrictive (bottom row) informative priors (see **Supplementary Figure 6**). Each coordinate represents a distinct set of length-dependent instability rates defined by parameters $(m, \tau_\epsilon, \tau_\kappa)$ shown in columns, x-axes and y-axes, respectively; τ_ϵ and τ_κ determine the power laws for expansion and contraction, respectively, and m is a multiplicative jump at $L = 9$ (parameterization defined in **Table 1**). Color indicates highest density range (HDR) of the posterior for specified total probabilities; black region sums to 0.01% of the probability. Red arrows show maximum posterior for each prior. Interpolation shifts posterior density to higher m values. **(b)** Comparison of empirical DRLs to inference results for each prior. Counts for all DRLs are necessarily normalized for comparison (see **Methods**); y-axis indicates normalized fractions (parentheses) and counts rescaled to match the number of repeats in the T2T genome (bold labels). Blue lines represent posterior-weighted DRLs for informative and uninformative priors; modeled DRLs are largely consistent with the empirical T2T DRL (black). Range shown in green represents the minimum and maximum counts at each length bin across all parameters in the 95% HDR under the uniform prior. Purple region shows the min-max range generated from $n = 34$ non-human primate genomes (after removing the two most-diverged DRLs and appropriately normalizing; see **Supplementary Figure 2**, **Methods**). Interpolation removes discontinuity in modeled DRLs between $L = 8$ and 9. **(c)** Posterior-weighted repeat instability rates. Tract length dependencies of expansion (blue) and contraction rates (orange) shown for specified priors. For comparison, empirical estimates are shown for pooled trios (squares; directly incorporated in model) and popSTR data (circles; used to construct informative priors). Informative priors show greater consistency with popSTR-estimated rates after interpolation, while the posterior-weighted DRLs (shown in **(b)**) remain consistent with the T2T genome.

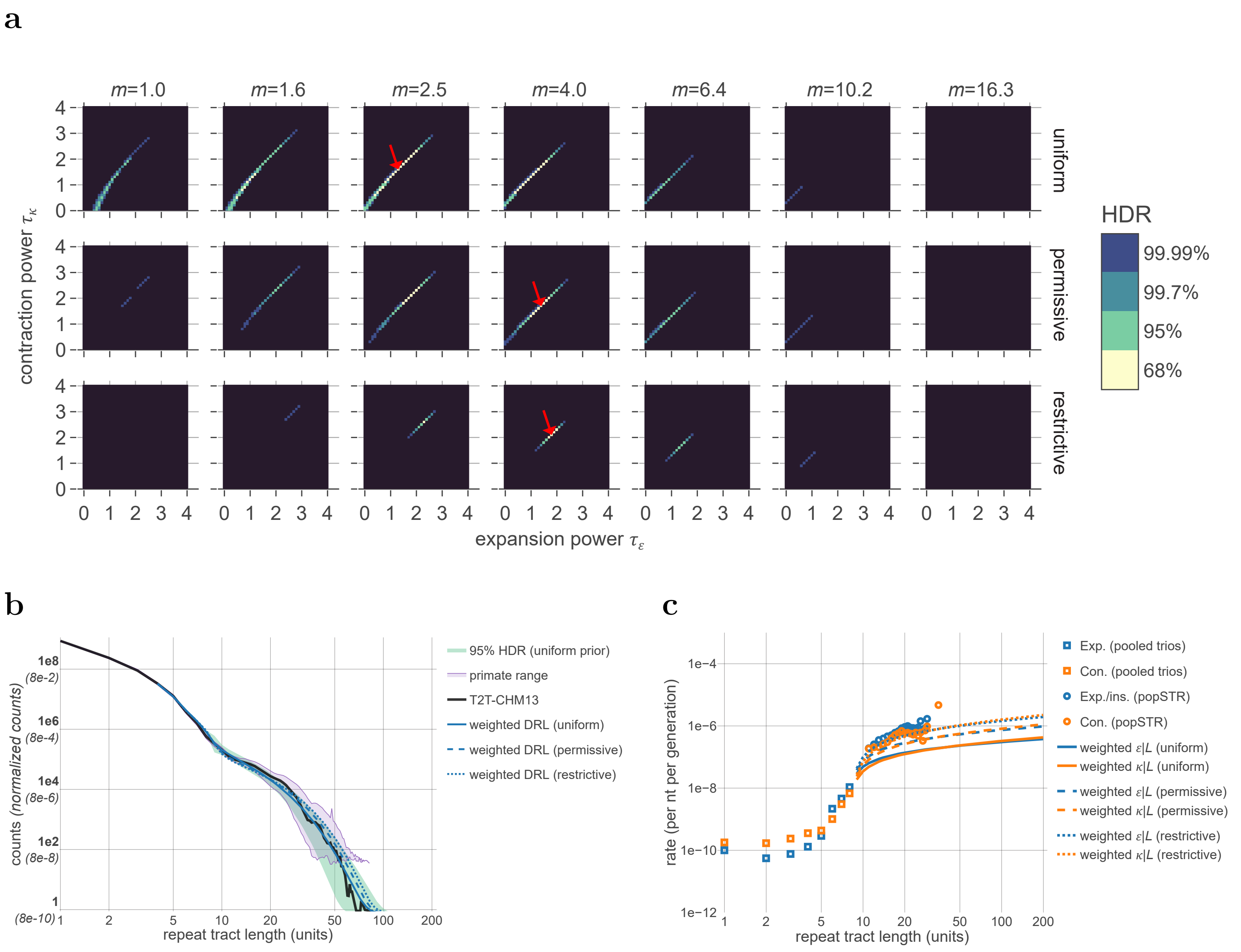

Supplementary Figure 9: *Posterior probability distributions under logarithm-based parameterization.* **(a)** Inference results for three-parameter multiplier-based model of repeat instability rates using a log-based functional form (see **Methods**). Plots display Bayesian posterior probabilities after applying uninformative (top row), permissive (middle row) and restrictive (bottom row) informative priors (see **Supplementary Figure 6**). The parameters $(m, \tau_\epsilon, \tau_\kappa)$ are defined analogously to the power-law model (see **Table 1** for parameterization). Color indicates highest density range (HDR) of the posterior for specified total probabilities; black region sums to 0.01% of the probability. Red arrows show maximum posterior for each prior. Posterior distribution is highly similar to that for the power-law parameterization, indicating that specific functional forms should not be over-interpreted. **(b)** Comparison of empirical DRLs to inference results for each prior. Counts for all DRLs are necessarily normalized for comparison (see **Methods**); y-axis indicates normalized fractions (parentheses) and counts rescaled to match the number of repeats in the T2T genome (bold labels). Blue lines represent posterior-weighted DRLs for informative and uninformative priors; modeled DRLs are largely consistent with the empirical T2T DRL (black). Range shown in green represents the minimum and maximum counts at each length bin across all parameters in the 95% HDR under the uniform prior. Purple region shows the min-max range generated from $n = 34$ non-human primate genomes (after removing the two most-diverged DRLs and appropriately normalizing; see **Supplementary Figure 2**, **Methods**). **(c)** Posterior-weighted repeat instability rates. Tract length dependencies of expansion (blue) and contraction rates (orange) shown for specified priors. For comparison, empirical estimates are shown for pooled trios (squares; directly incorporated in model) and popSTR data (circles; used to construct informative priors). Like the power-law parameterization, this parameterization allows simultaneous consistency with popSTR estimates and empirical DRLs.

a

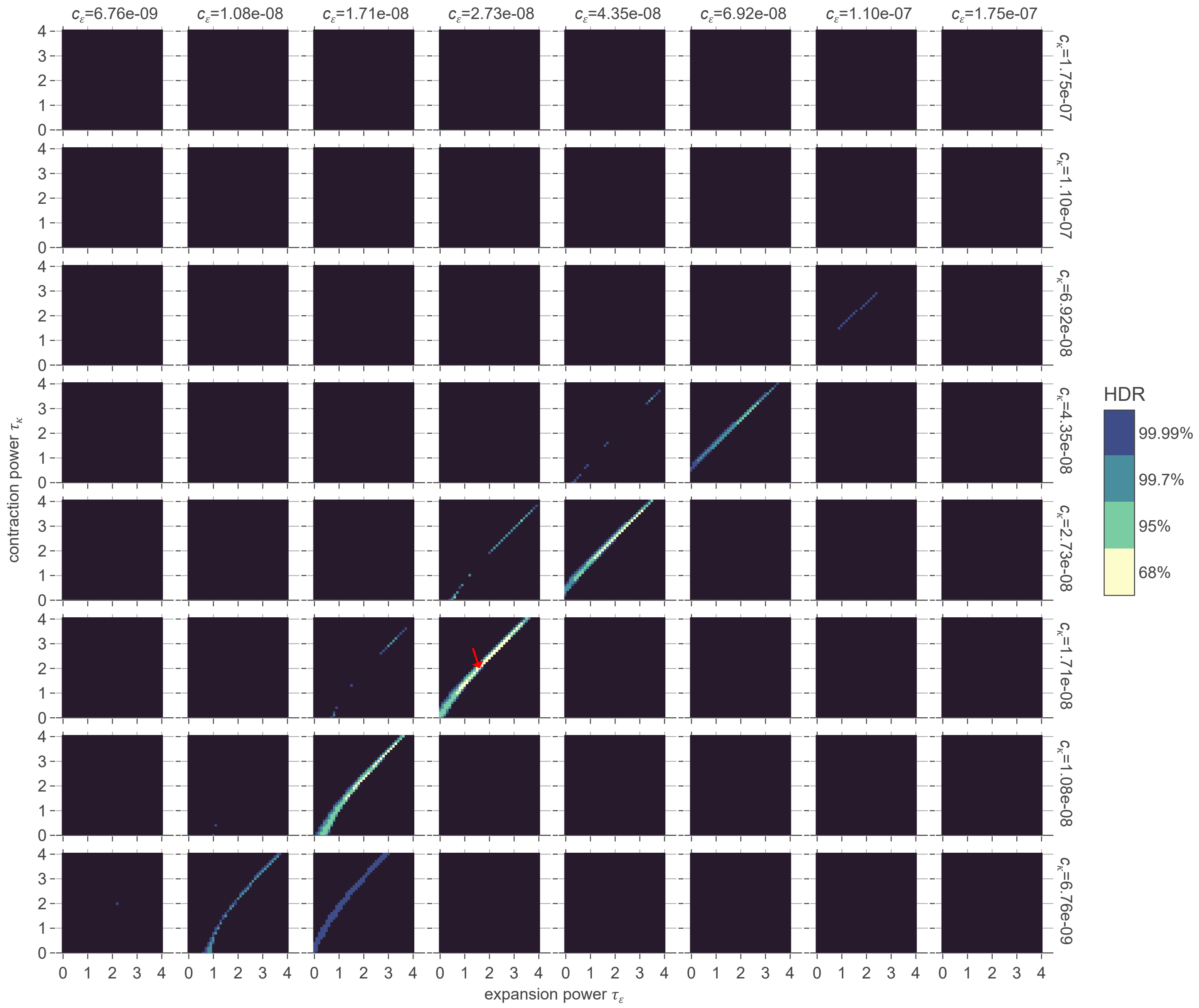

Supplementary Figure 10: *Inference results for four-parameter decoupled power-law model of repeat instability rates.* Bayesian posterior probabilities for the **(a)** uninformative prior, **(b)** permissive informative prior, and **(c)** restrictive informative prior. Each coordinate represents a distinct set of length-dependent instability rates defined by parameters $(C_\epsilon, C_\kappa, \tau_\epsilon, \tau_\kappa)$ shown in columns, rows, x-axes and y-axes, respectively; τ values determine power law exponents, and c values determine the rates at $L = 9$ (parameterization defined in **Table 1**). Color indicates highest density range (HDR) of the posterior for specified total probabilities; black region sums to 0.01% of the probability. Red arrows show maximum posterior. For all three priors, most of the density lies within the nested parameterization corresponding to the three-parameter multiplier-coupled model. **(d)** Comparison of empirical DRLs to inference results for each prior. Counts for all DRLs are necessarily normalized for comparison (see **Methods**); y-axis indicates normalized fractions (parentheses) and counts rescaled to match the number of repeats in the T2T genome (bold labels). Blue lines represent posterior-weighted DRLs for informative and uninformative priors; modeled DRLs are largely consistent with the empirical T2T DRL (black). Range shown in green represents the minimum and maximum counts at each length bin across all parameters in the 95% HDR under the uniform prior. Purple region shows the min-max range generated from $n = 34$ non-human primate genomes (after removing the two most-diverged DRLs and appropriately normalizing; see **Supplementary Figure 2**, **Methods**). **(e)** Posterior-weighted repeat instability rates. Tract length dependencies of expansion (blue) and contraction rates (orange) shown for specified priors. For comparison, empirical estimates are shown for pooled trios (squares; directly incorporated in model) and popSTR data (circles; used to construct informative priors). This parameterization provides a qualitatively similar set of observations to the three-parameter multiplier-coupled model (e.g., similar Bayes factors, posterior-weighted DRLs and instability rates are consistent with empirical estimates and display asymptotic contraction bias) but is less dependent on empirical rate estimates at $L = 8$.

(*continued on next page*)

b

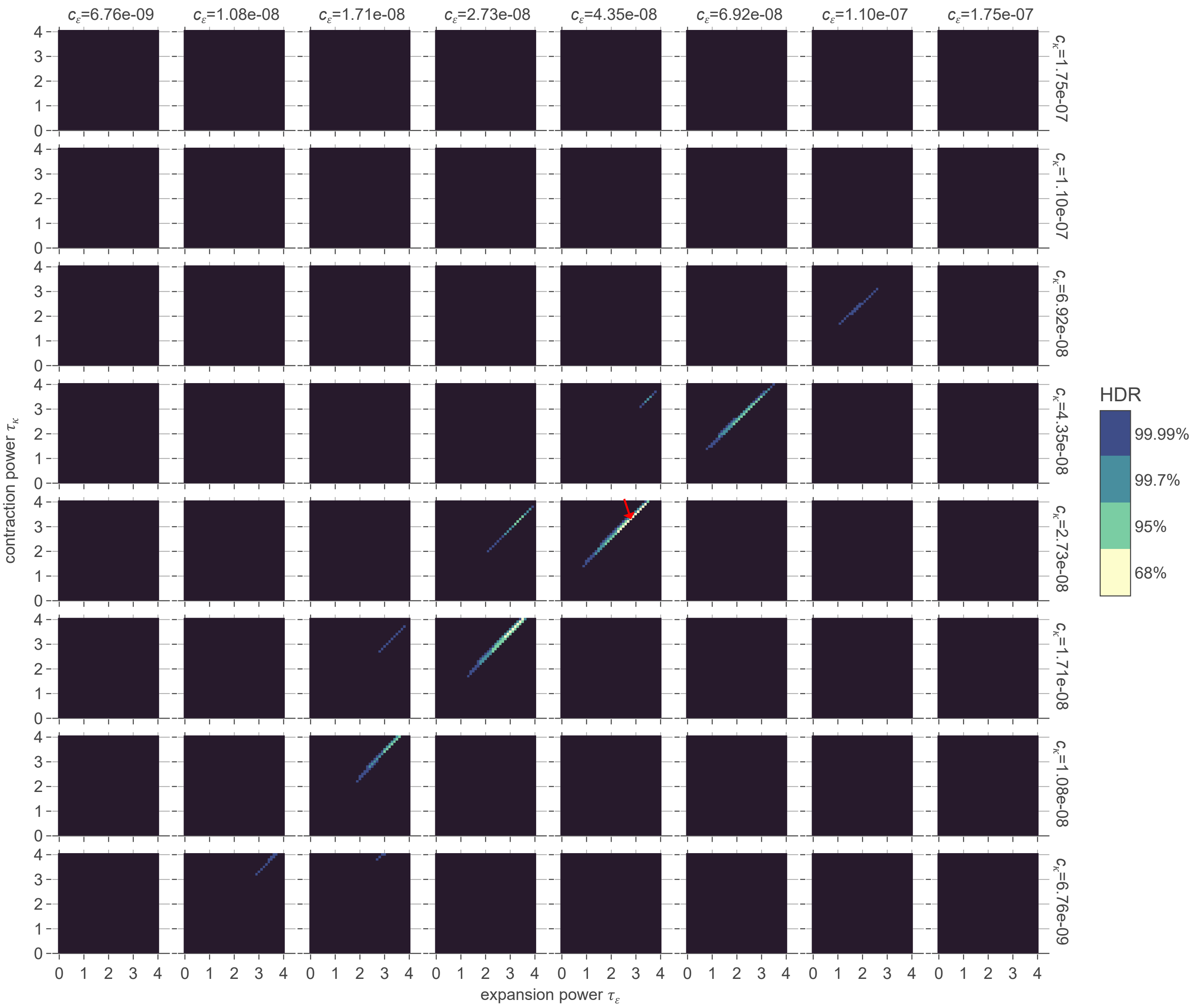

Supplementary Figure 10 (continued): *Inference results for four-parameter decoupled power-law model of repeat instability rates.* **(b)** Bayesian posterior probabilities for the permissive informative prior. (*continued on next page*)

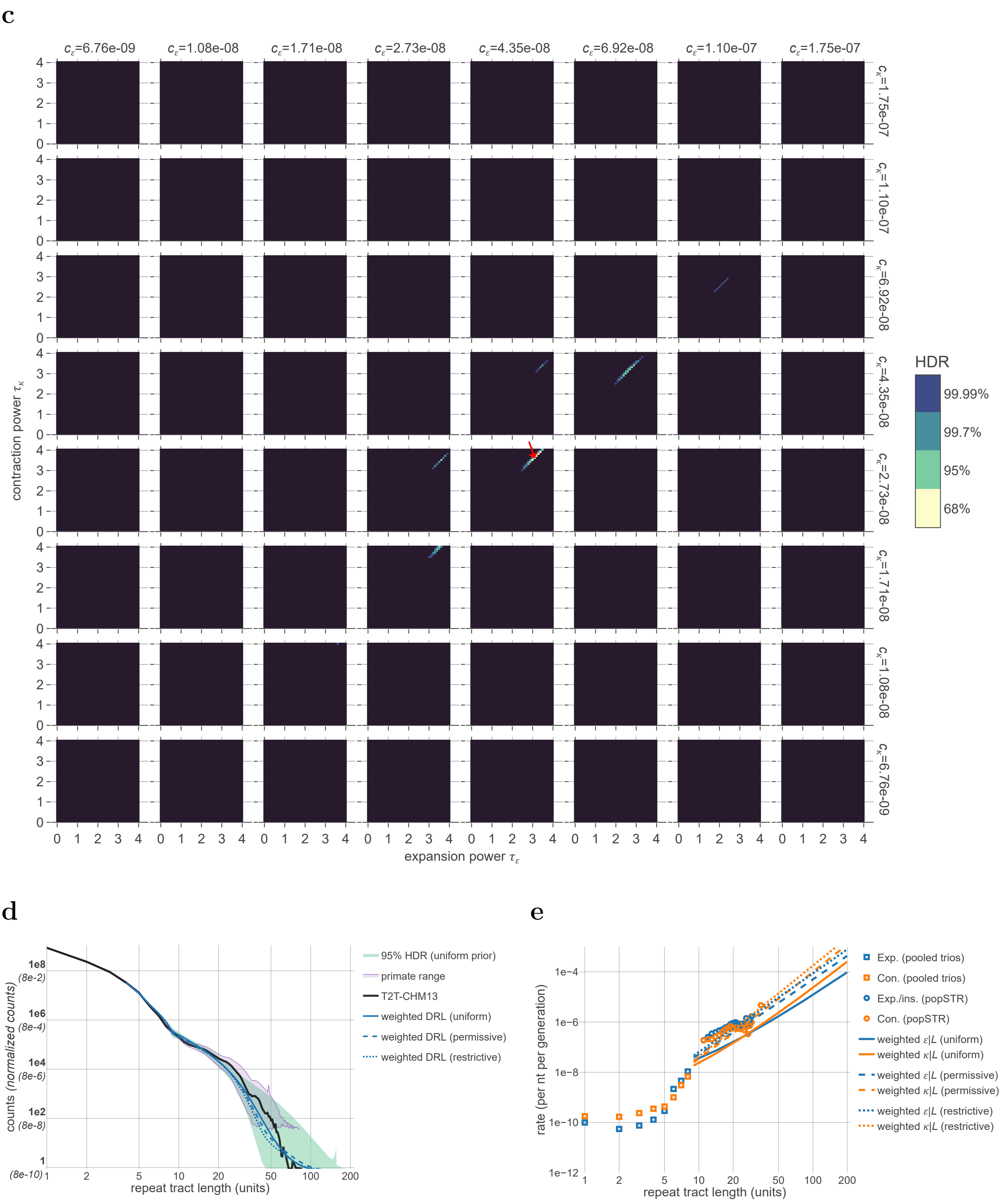

Supplementary Figure 10 (continued): *Inference results for four-parameter decoupled power-law model of repeat instability rates.* **(c)** Bayesian posterior probabilities for the restrictive informative prior. **(d)** Comparison of empirical DRLs to inference results for each prior. **(e)** Posterior-weighted repeat instability rates.

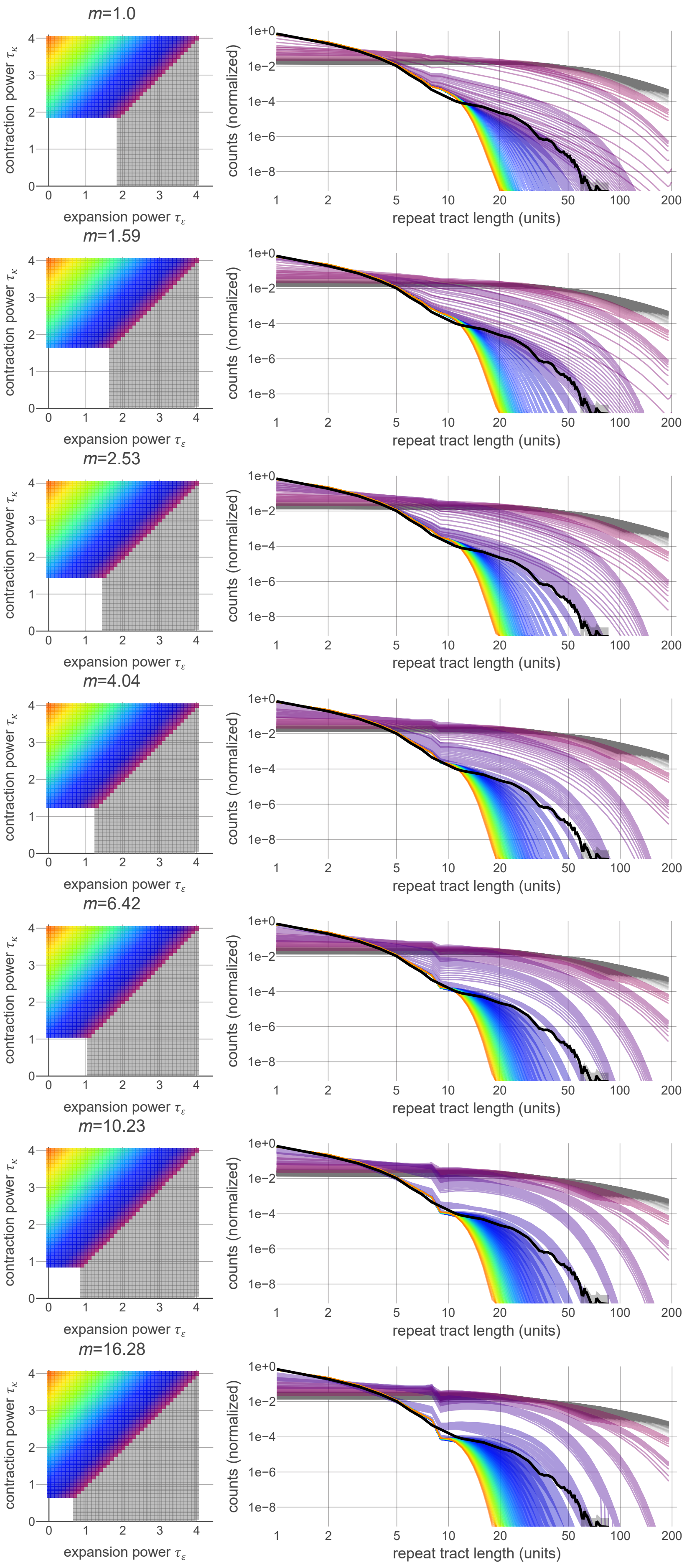

Supplementary Figure 11: *Late-time DRLs from computational model across parameter space.* Results from three-parameter multiplier-coupled power law model. **(left)** Grid of $\tau_\epsilon, \tau_\kappa$ parameter values for various multipliers m. For clarity, lower left region omitted due to low rates (i.e., insufficient to equilibrate in the allotted time). Lines of constant $\Delta\tau = \tau_\kappa - \tau_\epsilon$ are displayed in the same color. Red corresponds to large values of $\Delta\tau$, purple corresponds to low values of $\Delta\tau$, and gray represents negative values of $\Delta\tau$. **(right)** Plots of DRLs at the final time point, one for each parameter combination in the grid, using the same color-coding scheme. Black line depicts the empirical T2T DRL. Larger $\Delta\tau$ results in more rapid truncation of the distribution at lower lengths; smaller values of $\Delta\tau$ result in a more extended tail of long repeats. Negative values of $\Delta\tau$ result in unrealistic inflation of long tail; subsequent normalization leads to lower relative counts in short tract length bins. Higher m values result in larger discontinuity in the DRL between $L = 8$ and 9 units.

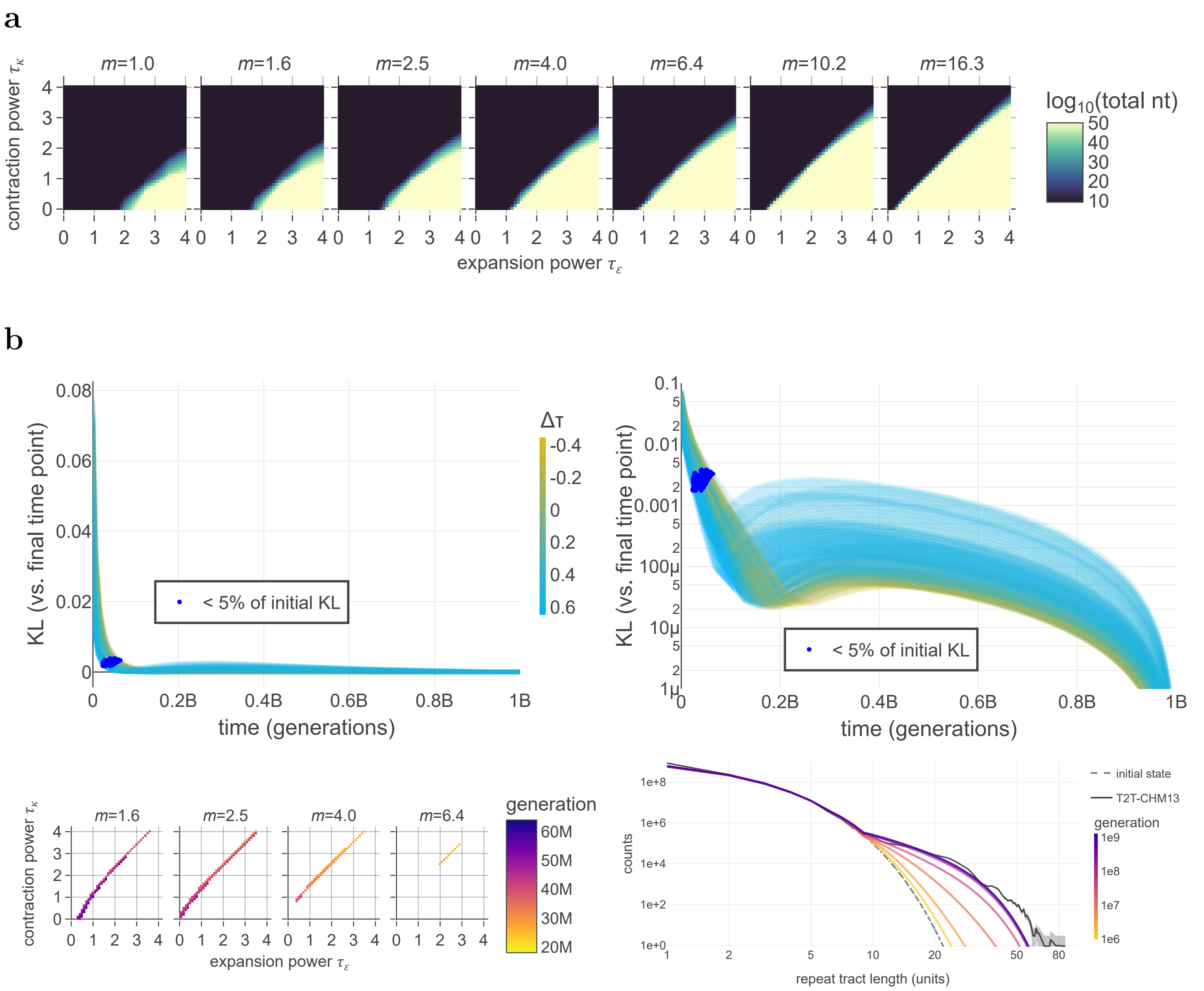

Supplementary Figure 12: *Constraints from genome size and equilibration time.* **(a)** Plot of total number of repeat bases in genome at final time point of computational model. Displayed as a heatmap across parameter space $(m, \tau_\epsilon, \tau_\kappa)$ for the three-parameter multiplier-coupled power-law model. Color specifies $\log_{10}$ of the sum of lengths of all repeats in the DRL. Color range is truncated at 10^{50}. Explosive genome growth occurs for heavily expansion-biased parameter combinations. **(b)** Equilibration time for computational model. **(top left)** Each line represents one parameter combination from the 95% HDR in the three-parameter multiplier-coupled model. Computational model initializes the DRL with the geometric distribution expected under substitutions alone. X-axis displays time in number of generations (under constant time rescaling). Y-axis displays KL divergence from the DRL at the final time point for each parameter combination (such that the KL divergence approaches zero in finite time). Color scale indicates values of $\Delta\tau = \tau_\kappa - \tau_\epsilon$. **(top right)** Same as in top left panel, with $\log_{10}$-scaled y-axis. For all parameter combinations, a two-stage equilibration process is evident, indicating an initial rapid phase in which the bulk of the extended tail of long repeats is established, followed by a slower phase of finer-scale changes. Blue dots indicate the time point at which the KL divergence first drops below 5% of the initial value for each parameter combination, marking the approximate end of the rapid phase. **(bottom left)** For parameters in the 95% HDR, the 5% KL divergence time point is displayed as a heatmap, showing that higher values of m, τ_ϵ, or τ_κ, (and larger $\Delta\tau$) result in faster establishment of the long repeat tail. While not a direct measure of time to achieve steady state, this indicates that, within the timescale of primate evolution, a relatively rapid change in the shape of the DRL is possible. **(bottom right)** Evolution of the DRL over time. Example shown for parameter combination with maximum posterior under uniform prior $(m, \tau_\epsilon, \tau_\kappa) = (2.5, 1.6, 2)$. Colored lines indicate state of the DRL after indicated number of generations (colored in log time). The shape of the DRL is largely established prior to 10^8 generations.

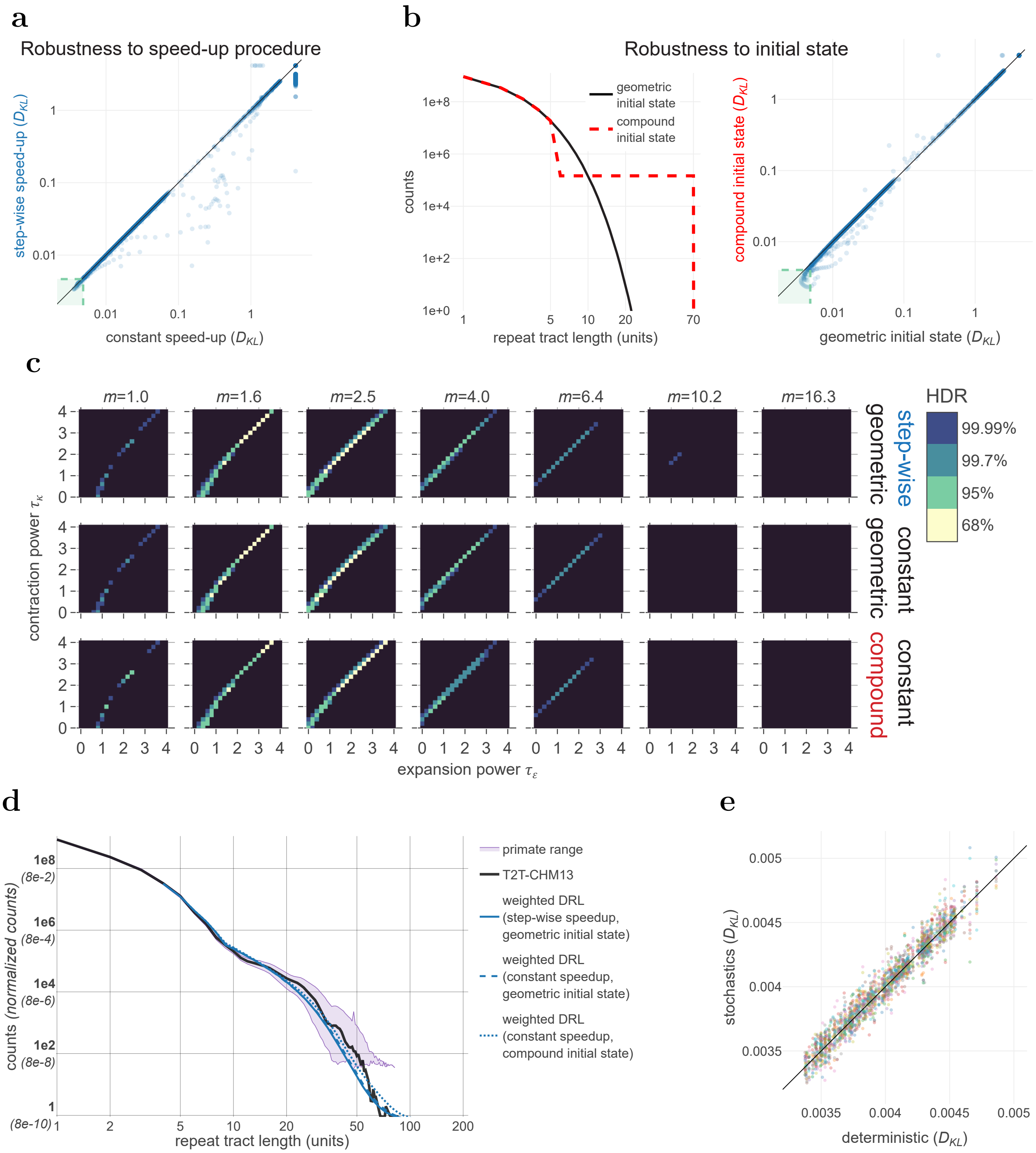

Supplementary Figure 13: *Demonstrations of robustness of inference procedure to simplifying approximations.* All comparisons below produced for the three-parameter multiplier-coupled power-law model. D_{KL} denotes KL divergence relative to the human T2T genome. **(a)** To confirm the validity of the computational speed-up procedure, we compared two different run conditions over a sparse grid of parameter combinations (step size = 0.2 for τ_ϵ and τ_κ). Plot shows correlation between D_{KL} for final timepoint DRLs, comparing runs with constant speed-up (x-axis) and step-wise speed-up (y-axis) procedures to rescale time. Dashed lines denote the boundary of the 95% HDR. **(b)** To confirm that the late-time DRL is independent of the initial condition, we compared runs initialized with two distinct DRLs with the same total weight. Left panel shows two initial DRLs: geometric DRL (used for all other runs; black) and a compound distribution (red) that is geometric with a uniform tail of long repeats. Right panel shows correlation between D_{KL} for final timepoint DRLs resulting from these initial states (over the same sparse grid of parameter combinations). The vast majority of parameter combinations in **(a)** and **(b)** are highly similar. **(c)** Bayesian posterior distributions for the three run conditions included in **(a)** and **(b)**. Color bar indicates highest density range (HDR) of the posterior for specified total probabilities; black region sums to 0.01% of the probability. **(d)** Correspondence between all three posterior distributions in **(c)**, along with posterior-weighted DRLs (shown here), demonstrates robustness of the inference to the computational speedup procedure and initial condition. **(e)** Effect of stochastics on computational model results. Poisson noise was introduced around the number of mutational transitions in each length bin per generation (see **Methods**). n = 10 stochastic runs are shown for each parameter combination in the 95% HDR (see **Figure 5a**). Plot shows the limited impact of stochastics on the D_{KL} summary statistic relative to the 95% HDR (and the empirical range seen in primates).

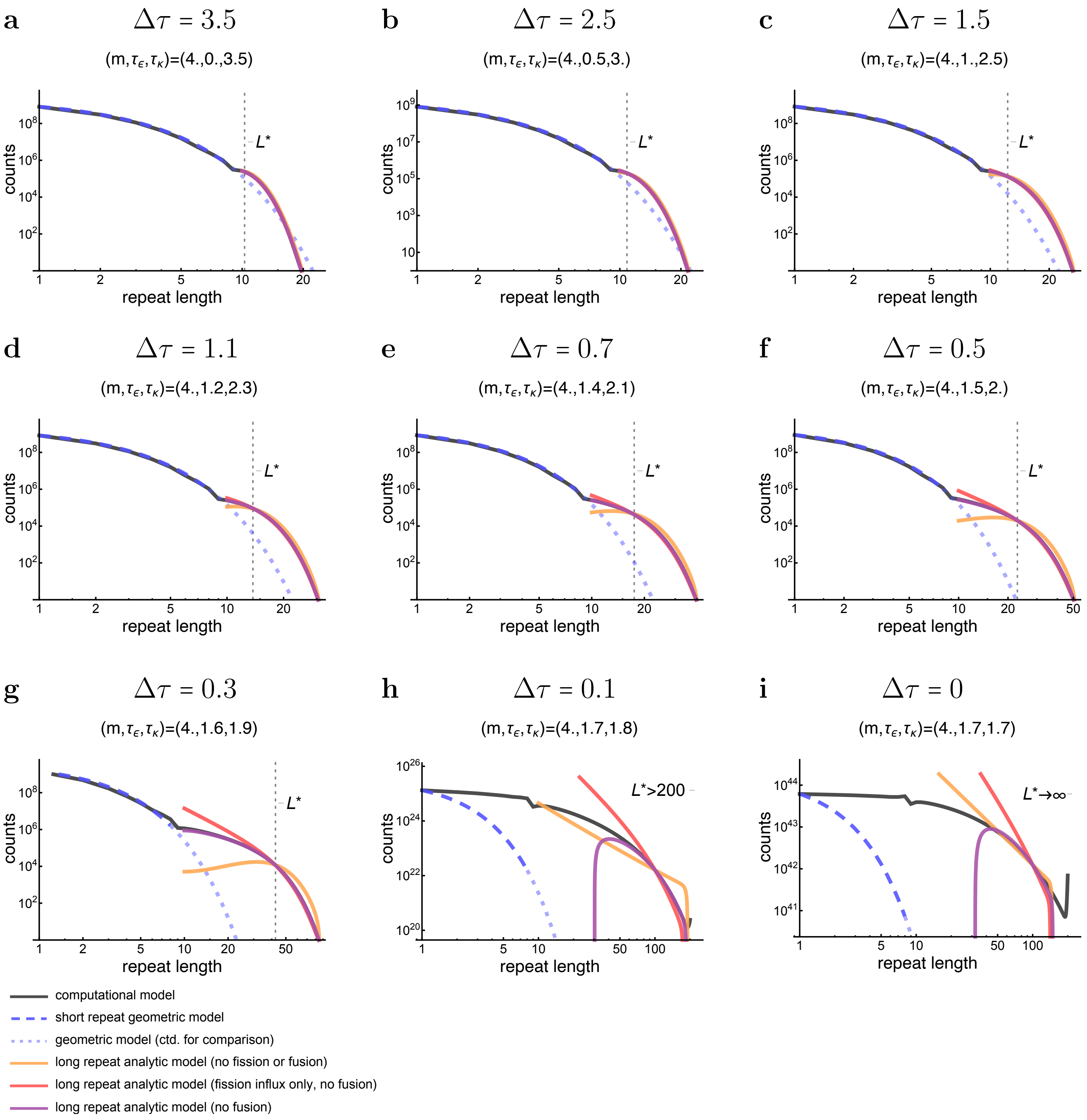

Supplementary Figure 14: *Comparison between computational model results and numerical solutions to steady state equations for $m = 4$ for parameter values with of constant $\tau_\epsilon + \tau_\kappa = 3.5$.* For contrast, $(\tau_\epsilon, \tau_\kappa) = (1.7, 1.7)$ ($\Delta\tau = 0$; $\tau_\epsilon + \tau_\kappa = 3.4$) provides an example of $\tau_\epsilon = \tau_\kappa$. Each inset shows plots of the computationally modeled distribution at the final time point (black), geometric approximation for shorter repeats of length $L < 10$ (blue, continued as blue dashed line for comparison to distribution tail shape), numerical solutions to Equation S46 with no fission (orange), numerical solutions to Equation S45 with fission out but no fission in (red), and numerical solutions to Equation S51 with fission (purple). **(a, b)** Comparisons for parameter combinations with $\Delta\tau > 1.5$ (referred to as $\Delta\tau \gg 1$) show good agreement for all numerical solutions; fission is negligible. **(c)** Boundary between large $\Delta\tau$ and intermediate values where fission out first becomes relevant. **(d, e)** Fission outflux becomes relevant when $\Delta\tau < 1.5$. Local approximation (orange) underestimates DRL for $L < L^*$ and overestimates $L > L^*$ (though, approx. remains close near L_{trunc}). **(f, g)** Fission influx is relevant for $L < L^*$, while the DRL for $L > L^*$ is well approximated when considering only fission outflux and local dynamics (i.e., treating fission influx as negligible). Under informative prior, $\Delta\tau = 0.5$ for all parameters in 95% HDR (i.e., panel (f) represents realistic regime for humans; uninformative prior suggests similar values $\Delta\tau \sim 0.4$–0.6). **(h)** L^* lies far above computational grid boundary ($L_{\mathrm{bound}} = 200$). DRL would truncate at a length $L \gg 200$ and stabilize if the grid was extended far beyond realistic lengths. **(i)** Unstable dynamical regime subject to nonlinear growth. The distribution shows clear interaction with the reflecting boundary.

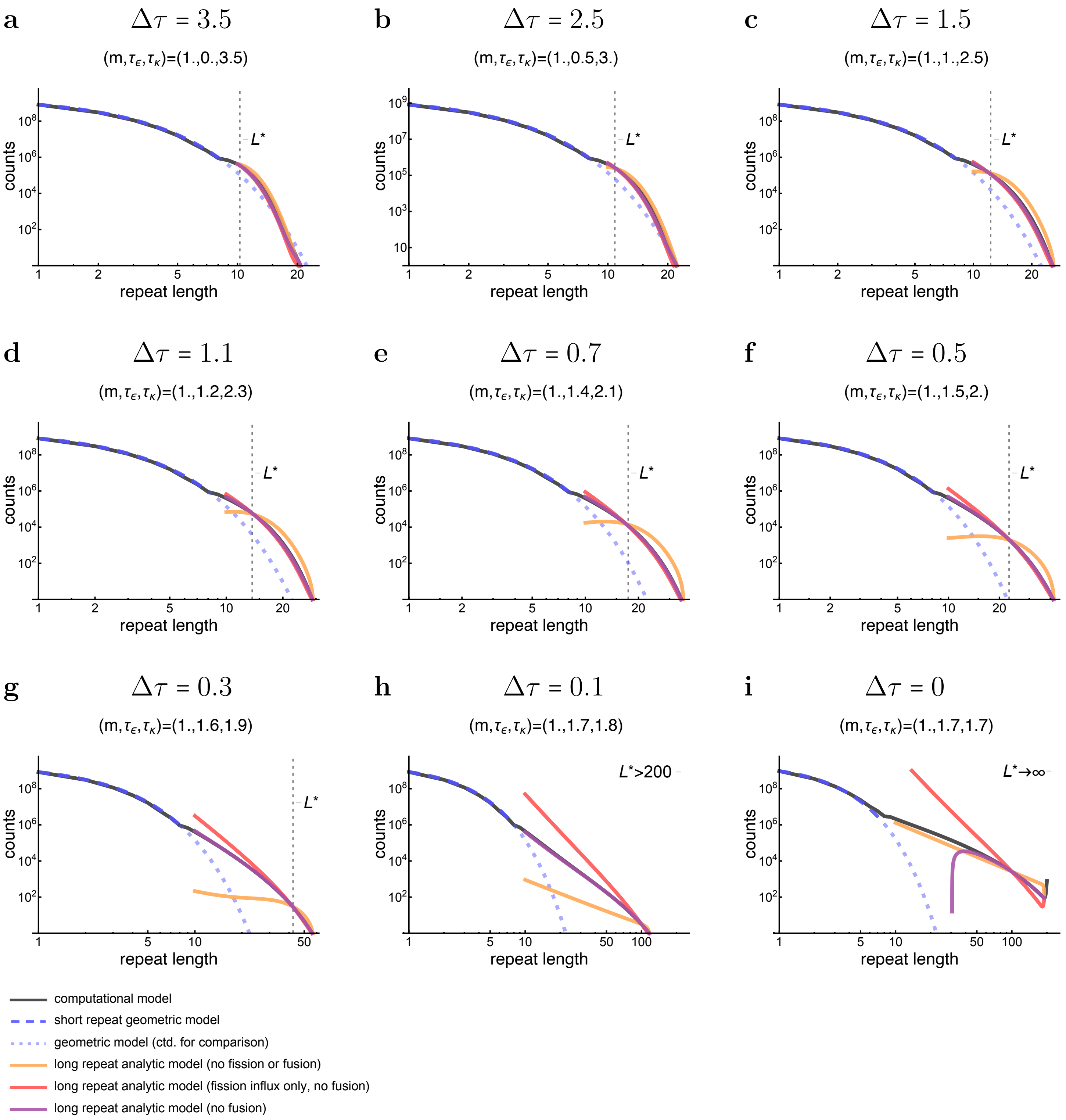

Supplementary Figure 15: *Comparison between computational model results and numerical solutions to steady state equations for m = 1.* Computationally modeled distributions are plotted for the same $(\tau_\epsilon, \tau_\kappa)$ (and therefore $\Delta\tau$) parameters plotted in **Supplementary Figure 14** (shown as the same inset panel), but for $m = 1$. Each inset shows plots of the computationally modeled distribution at the final time point (black), geometric analytic approximation for shorter repeats of length $L < 10$ (blue, continued as blue dashed line for comparison to distribution tail shape), numerical solutions to Equation S46 with no repeat fission (orange), numerical solutions to Equation S45 with fission out but without fission in (red), and numerical solutions to Equation S51 with fission out and fission in (purple). As expected, the local approximation (orange) deviates at larger $\Delta\tau$ for smaller m; substantial deviation can be seen when $\Delta\tau \leq 1.5$. The qualitative properties of each approximation are unchanged.

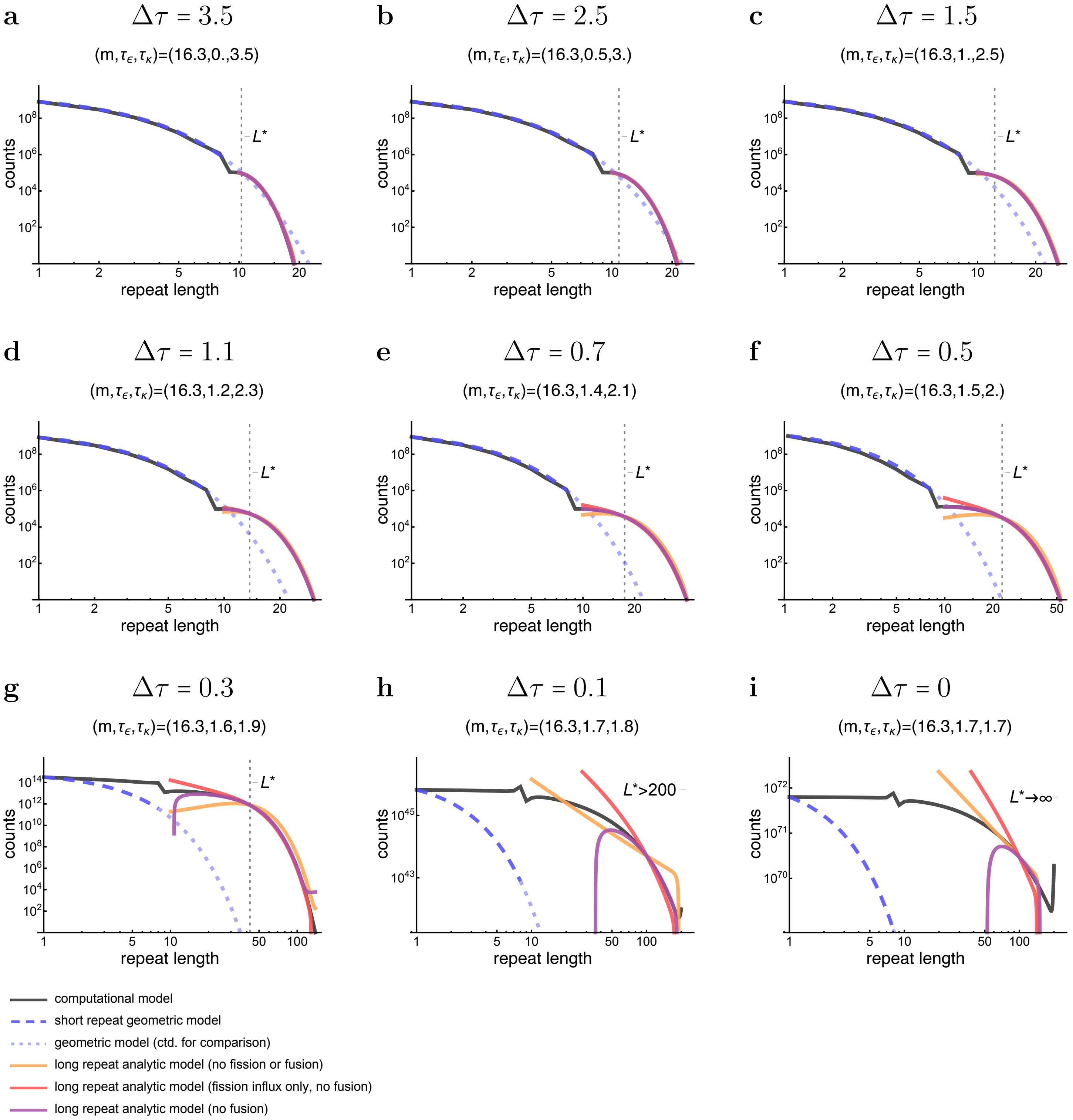

Supplementary Figure 16: *Comparison between computational model results and numerical solutions to steady state equations for* $m = 16.3$. Computationally modeled distributions are plotted for the same $(\tau_\epsilon, \tau_\kappa)$ (and therefore $\Delta\tau$) parameter combinations plotted in **Supplementary Figure 14** (shown in the same location), but for $m \approx 16$. Each inset shows plots of the computationally modeled distribution at the final time point (black), geometric analytic approximation for shorter repeats of length $L < 10$ (blue, continued as blue dashed line for comparison to distribution tail shape), numerical solutions to Equation S46 with no repeat fission (orange), numerical solutions to Equation S45 with fission out but without fission in (red), and numerical solutions to Equation S51 with fission out and fission in (purple). As expected, the local approximation (orange) deviates at smaller $\Delta\tau$ for larger m; in this case, deviation is only visible when $\Delta\tau < 1$. The qualitative properties of each approximation are unchanged.

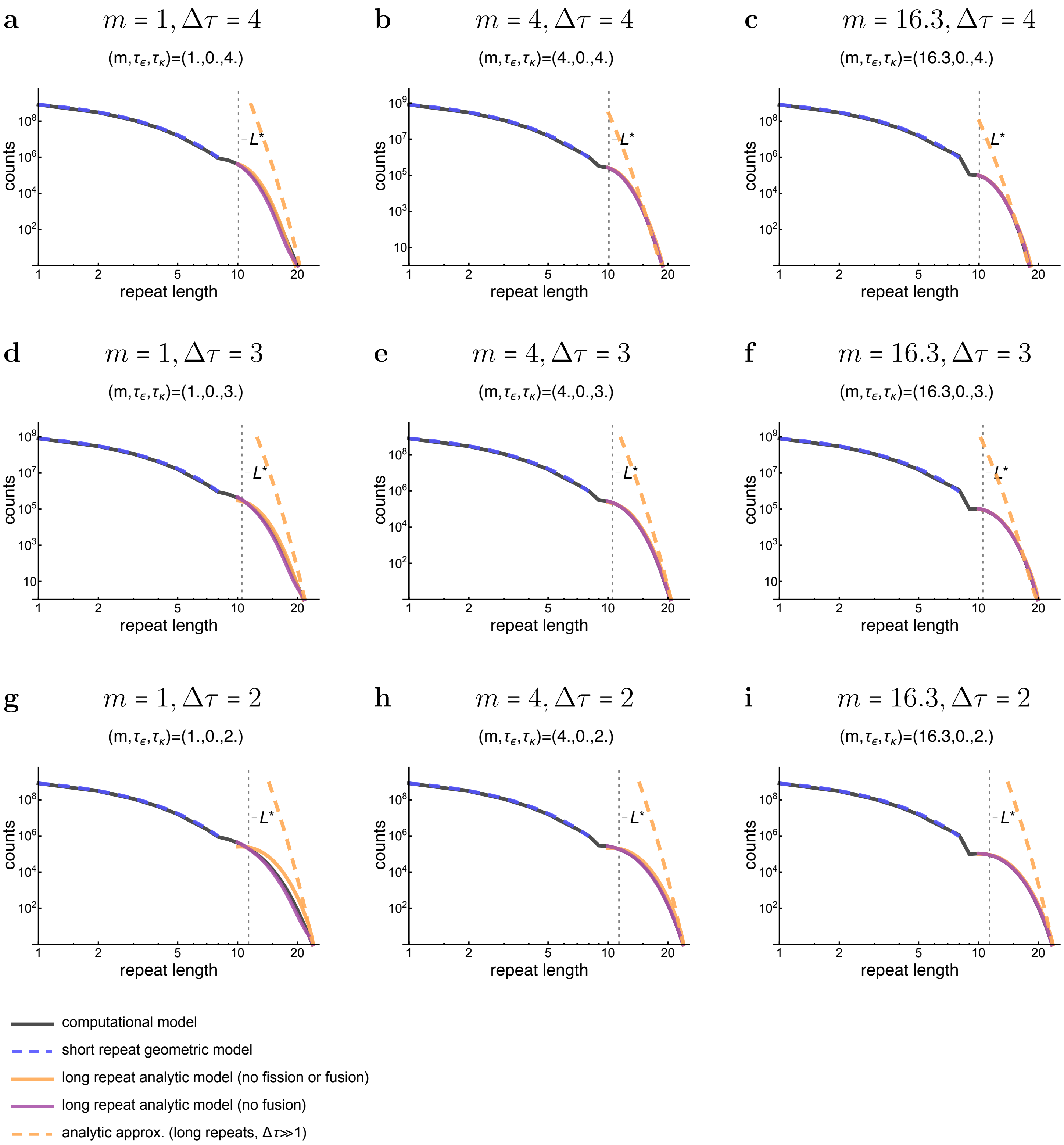

Supplementary Figure 17: *Accuracy of analytic approximation for distribution falloff in the* $\Delta\tau \gg 1$ *regime.* The rough approximation in Equation S48 to the shape of the steady state distribution when $\Delta\tau \gg 1$ is shown for a range of parameter combinations with $\Delta\tau \geq 1$. Each row plots the same combination of $(\tau_\epsilon, \tau_\kappa)$ for multipliers $m = 1$ (left: **(a)**, **(d)**, **(g)**), $m = 4$ (center: **(b)**, **(e)**, **(h)**), and $m \approx 16$ (right: **(c)**, **(f)**, **(i)**). Each column plots the same value of m for $\Delta\tau = 4$ (top: **(a)**–**(c)**), $\Delta\tau = 3$ (middle: **(d)**–**(f)**), $\Delta\tau = 2$ (bottom: **(g)**–**(i)**). For larger $\Delta\tau$ and m values, the analytic solution approximates the computationally modeled and numerically generated distributions, except at lengths adjacent to the short repeat regime (roughly $13 > L > 10$, noting axes are log spaced). At very low m and lower $\Delta\tau$, the local approximation deviates numerically, indicating further analytic approximation used to solve this equation poorly approximates the steady-state DRL.

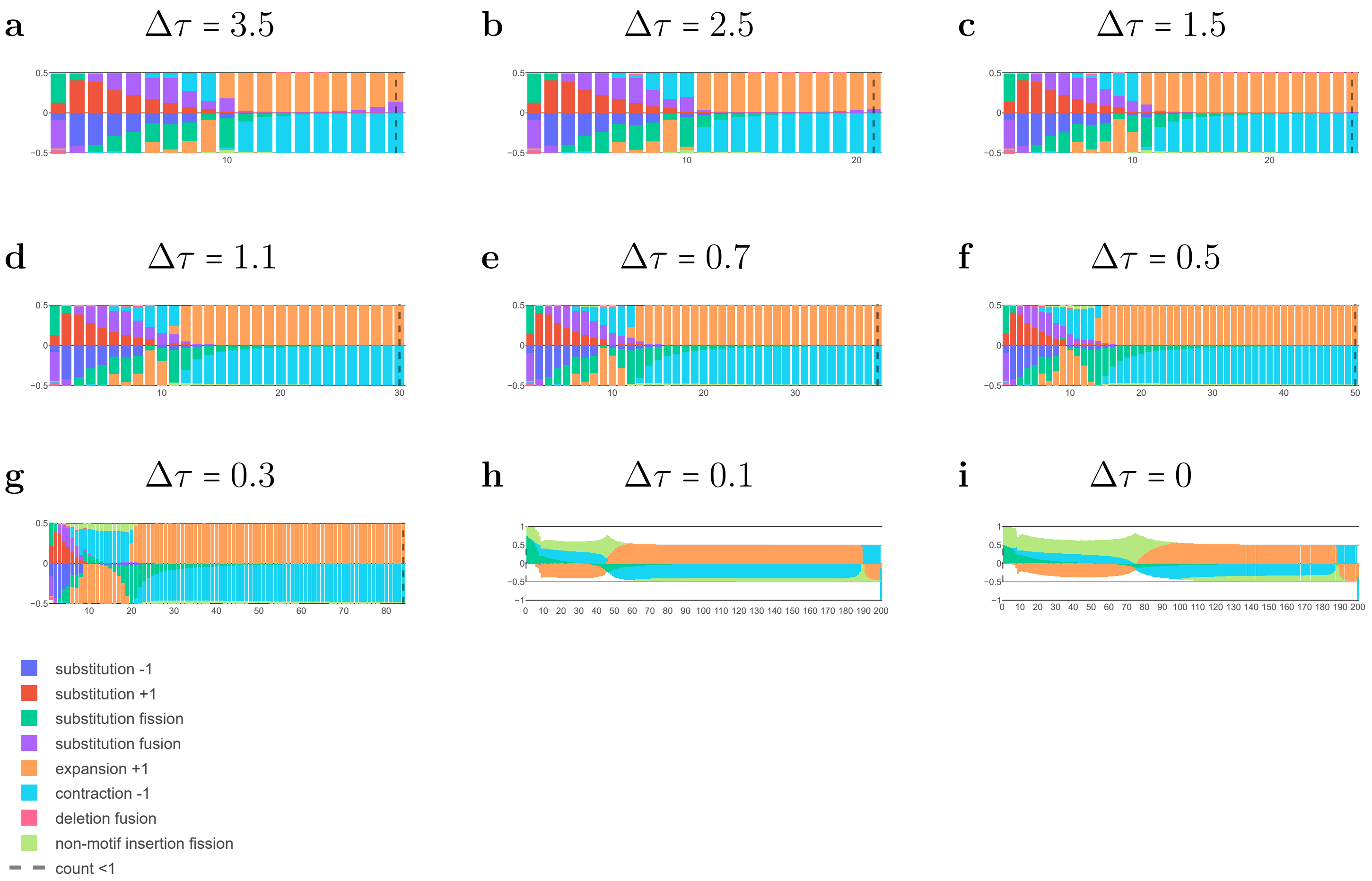

Supplementary Figure 18: *Computational model results for the net flux per mutation type.* Subplots **(a)**–**(i)** correspond to the same parameters shown in **Supplementary Figure 14**. After the final time point of each run, flux in and out of each bin was calculated and attributed to the following transitions: local length increase due to μ substitutions (darker blue); local length decrease due to ν substitutions (darker red); nonlocal fission generated by ν substitutions (darker green); nonlocal fusion due to μ substitutions (purple); local length increase due to expansion (orange); local length decrease due to contraction (lighter blue); nonlocal fusion resulting from deletion of a B base (lighter red); nonlocal fission due to non-motif insertions (lighter green). Dashed black line shows longest populated length L_{trunc} where $\rho(L > L_{\mathrm{trunc}}) < 1$. Net flux per category was computed as flux in minus flux out (i.e., net change in the number of repeats per length class per mutational transition). After computing the net flux for each effect, the sum of magnitudes of all effects was separately normalized at each length (i.e., height of stacked bars sums to one). If a given transition results in a net influx (outflux), associated bar appears above (below) the axis. Bins showing identical heights above and below zero are maintained in detailed balance.

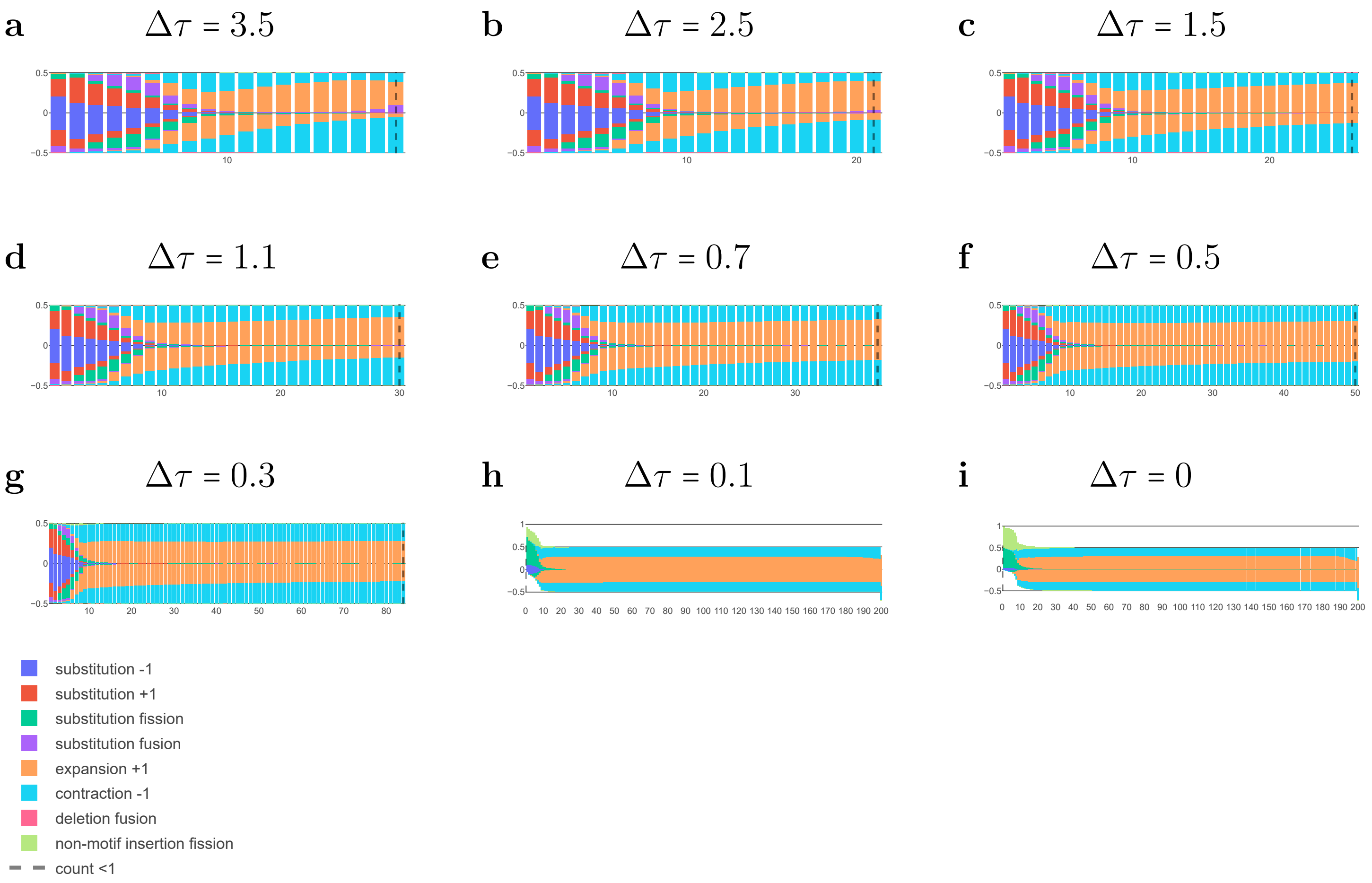

Supplementary Figure 19: *Computational model results for directional flux per mutation type.* Subplots **(a)**–**(i)** correspond to the same parameters shown in **Supplementary Figure 14**. After the final time point of each run, flux in and out of each bin was calculated and attributed the same mutational processes as in **Supplementary Figure 18** (shown in corresponding colors). For each category, flux in and flux out are plotted separately at each length (shown above and below zero, respectively). The sum of the magnitudes of all effects (influxes plus outfluxes) was separately normalized to one at each length. Bins showing identical heights above and below zero (i.e., influx equal to outflux) are maintained in detailed balance. In contrast to **Supplementary Figure 18**, each bar height represents the fraction of total number of transitions (in either direction) due to each signed mutational transition (e.g., fraction of number of transitions from expansion influx events, expansion outflux events, contraction influx events, etc.).

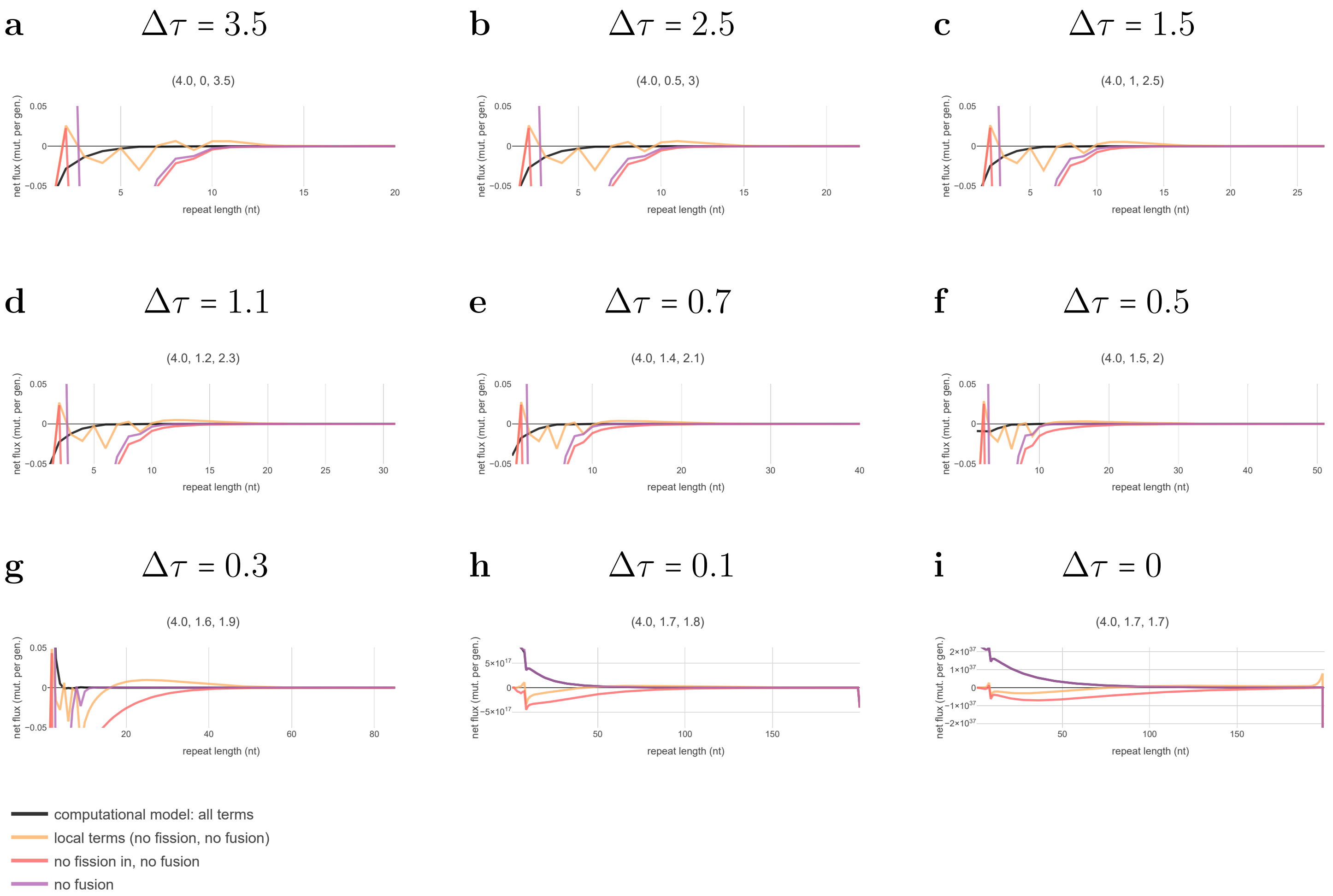

Supplementary Figure 20: *Computational model results for collective non-normalized fluxes showing the relevance of local transitions, fission, and fusion.* Subplots **(a)**–**(i)** correspond to the same parameters shown in **Supplementary Figure 14**. At the final iterated time point, fluxes were calculated for each length class and summed appropriately to test the accuracy of different analytic models of the long length regime specified in Equations S37, S45, and S46. Each model specifies an approximate steady state equation assembled as a subset of the full collection of terms shown in Equation S32. Equation S32, which includes repeat fusion, was summarized by adding the fluxes due to all mutational effects separately at each length (shown in black); detailed balance occurs when all fluxes sum to zero at a given length. Each approximation is deemed appropriate at lengths where they overlap the black curve (restricted to $L > 10$). In contrast to **Supplementary Figure 14**, which tests the accuracy of solutions to the approximated steady state equations, this comparison tests the differential equation more directly by specifying the magnitude of individual terms in the expression (within a given parameter and length regime); in particular, this comparison captures nonlocal effects in Equation S37 directly, without reference to Equation S37. The model missing only fusion (Equation S37; purple) deviates from the full model (Equation S32; black) only for $L \lesssim 10$ indicating fusion is negligible in the long repeat regime. All three approximations overlap for large $\Delta\tau$, indicating the dominant behavior is local (described by Equation S46; yellow); the model with fission treated strictly as an outflux (Equation S45; red) remains a good approximation to the full effects of fission (purple) above roughly $\Delta\tau \sim 0.5$. **(h)**–**(i)**: The reflective boundary imposed in our computational model generates artefactual spikes in fluxes near $L_{\text{bound}} = 200$ due to nonzero counts in these length classes.

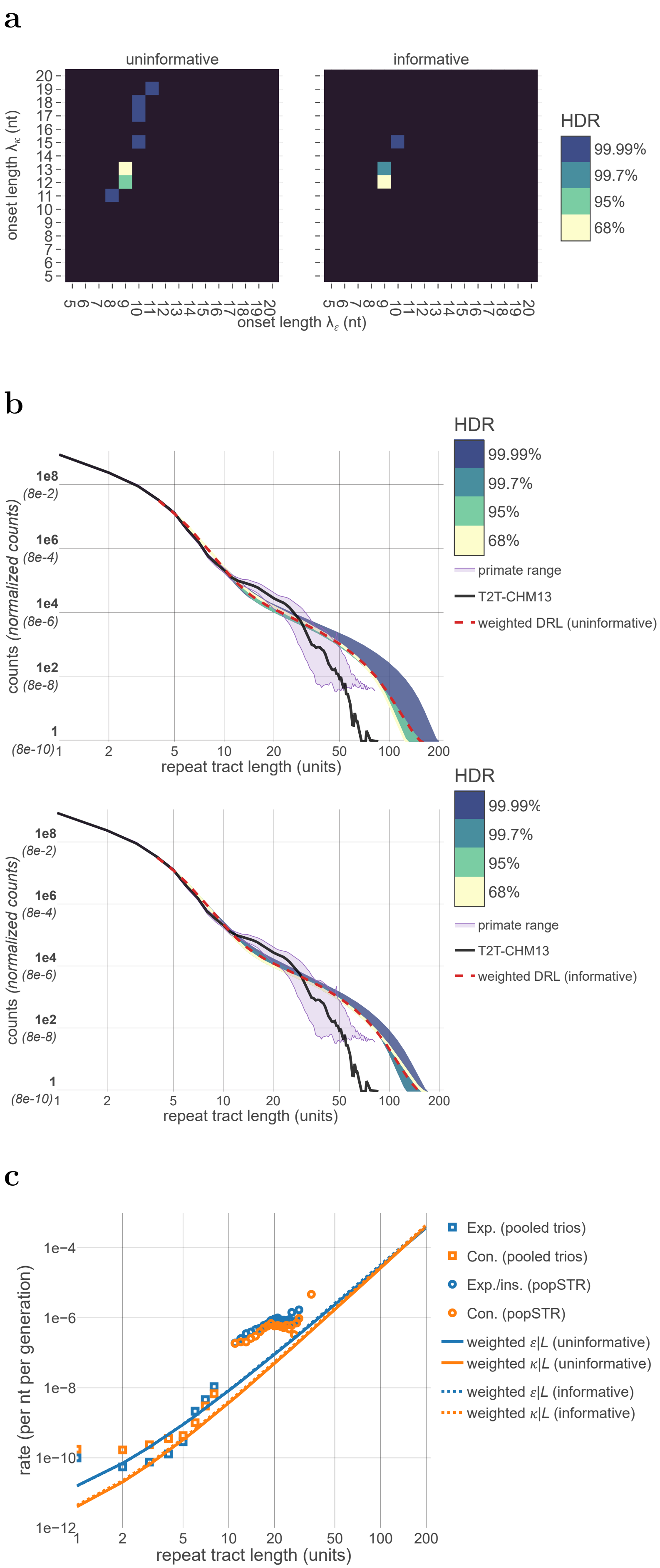

Supplementary Figure 21: *Pure power-law parameterization and the onset length of repeat instability.* Bayesian inference results for the pure power-law parameterization (specified in **Table 1**). This model parameterizes expansion and contraction rates at all tract lengths with no reliance on empirical mutation rate estimates; inference results represent information contained within the DRL alone (rate estimates only incorporated in the informative prior). To infer the onset length of repeat instability, the expansion rate was parameterized in terms of λ_ϵ, the length at which the rate first exceeds the average substitution rate μ (lengthening; B>A). Analogously, λ_κ represents the length at which the contraction rate exceeds the average substitution rate ν (shortening; A>B). Exponents τ_ϵ and τ_κ parameterize the power-law length dependencies for expansion and contraction, respectively. **(a)** To study the onset lengths, the inferred posterior was marginalized over τ_ϵ and τ_κ to produce marginal posterior probabilities for $(\lambda_\epsilon, \lambda_\kappa)$. Color indicates highest density range (HDR) of the marginal posterior for specified total probabilities; black region sums to 0.01% of the probability. Marginal posteriors are highly localized under both an uninformative (left) and informative prior (right). **(b)** Comparison of empirical DRLs to inference results under uninformative and informative priors. Counts for all DRLs are necessarily normalized for comparison (see **Methods**); y-axis indicates normalized fractions (parentheses) and counts rescaled to match the number of repeats in the T2T genome (bold labels, black curve). Dashed line represents posterior-weighted DRL. Colored ranges represent the minimum and maximum counts at each length bin across all parameters in the specified HDR (color corresponds to panel a). Purple region shows the min-max range generated from $n = 34$ non-human primate genomes (after removing the two most-diverged DRLs and appropriately normalizing; see **Supplementary Figure 2**, **Methods**). This toy model recapitulates the important qualitative features of the empirical DRL (i.e., deviation from geometric distribution at 10 nt corresponding to λ values, followed by an extended tail of repeats that truncates at finite length). **(c)** Comparison of expansion (blue) and contraction rates (orange) to posterior-weighted length dependence for specified priors. Length dependence shows initial expansion bias and asymptotic contraction bias (the latter observable in all parameterizations).

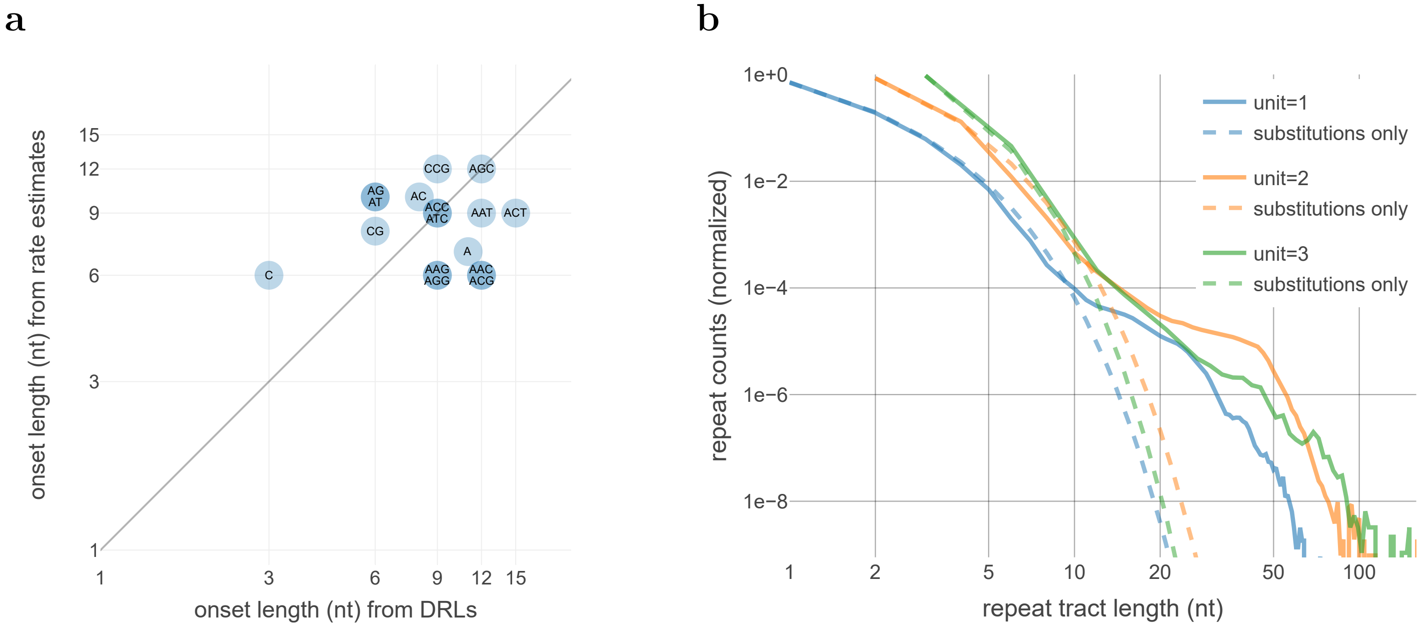

Supplementary Figure 22: *Assessing the onset length of repeat instability for longer motifs.* **(a)** Onset length of repeat instability as calculated from the deviation of the empirical DRL from geometric decay (see panel b) on the x-axis, and from the comparison of per-repeat expansion and contraction rates to μ substitution rates on the y-axis. For both measurements, the length is found where a two-fold deviation first occurs. Motifs are indicated in text overlays; all motifs with unit length ≤ 3 nt are shown. Apart from two outliers (C and ACT), all lengths are within a range of 6—12 nt. **(b)** Solid lines display normalized DRLs in CHM13-T2T summed across mono-, di-, and trinucleotide motifs. Dashed lines represent a computational model of the substitution process alone (omitting all indels). Computational models were separately run for each motif; resulting DRLs were summed according to motif length. This results in a sum of geometric distributions, which describes the low-length (i.e., $<$ 10 nt) portion of each empirical distribution. Empirical deviation from this distribution results from the transition to repeat instability at longer lengths.

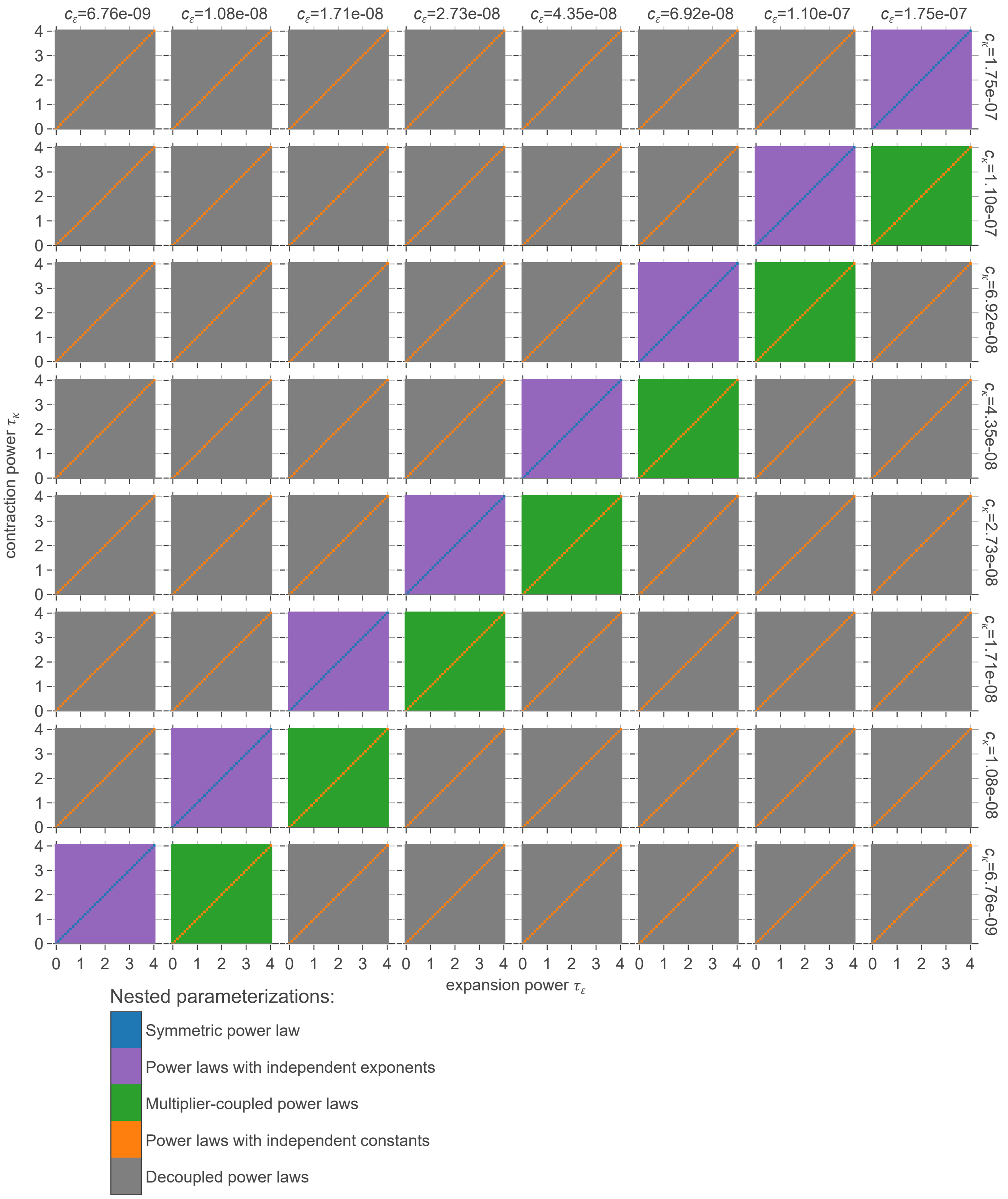

Supplementary Figure 23: *Parameterization nesting procedure.* For computational efficiency and straightforward interpretation of Bayes factor ratios, all parameterizations were nested in a four-dimensional grid of parameters associated with the four-parameter decoupled power law model (all points). Each inset plane depicts a grid of $(\tau_\epsilon, \tau_\kappa)$ values (x-axis, y-axis) denoting the exponents of power-laws for expansion and contraction rates, respectively. Constants C_ϵ and C_κ that determine the expansion and contraction rates at length $L = 9$ units are represented as columns and rows, respectively. Parameterizations with fewer degrees of freedom (defined in **Table 1**) are embedded as lower-dimensional slices of the four-dimensional grid and shown in specified colors. Note that the power-law model with independent constants (orange) appears disconnected in this representation but is simply connected in the discrete four-dimensional space.

Supplementary Note

1 Analytic modeling of the repeat length distribution in steady state

To better understand our observations, we sought to describe the equilibrium that emerges once the distribution of repeat tract lengths (DRL in main text; herein, denoted P_L in discrete form and $\rho(L)$ in the continuum) has reached steady state at asymptotically late times (i.e., as $t \to \infty$, $P(L;t) \to \mathrm{P}_{ss}(L)$, where ss denotes steady state). Importantly, for any given parameterization, this does not occur for all parameter combinations, as a subset remain inherently unstable. For the parameter values that stabilize, the shape of the distribution is described by a dynamic balance that emerges between mutational processes, with distinct mutational effects dominating the dynamics in different length regimes. For simplicity, the analysis provided here is restricted to the case of mononucleotide repeats consisting of various numbers of A bases. Alternative bases that terminate each sequence, labeled B, represent any T, C, or G base (i.e., any non-motif base for A repeats) at either end of an A repeat. The distribution of tract lengths for B strings will be ignored throughout. Further, our analytic treatment focuses on the three-parameter multiplier-coupled parameterization (see **Methods**, **Table 1**, Equation S1; henceforth, 'multiplier model') describing the length dependence of the set of repeat instability rates; this parameterization was determined to have the strongest statistical support amongst models tested (see **Table 1**, **Results**). Where possible, dynamical equations are provided in general form in terms of an arbitrary parameterization of the rates.

1.1 Mutational processes affecting repeats

As described in the manuscript, the mutational processes that are included in our model are the following, enumerated here with corresponding variables (ϵ_L, κ_L, etc.) that represent the (parameterized) length-dependent rates of each mutation type. For the reader's convenience, a schematic representation of these mutational processes is provided in **Figure 2a** along with corresponding effects on repeat length shown in **Figure 2b**.

1. *Lengthening substitutions* μ: Point mutations $B \to A$ that increase the length of a repeat while preserving the total length of the genome. Such mutations only increase repeat length when they occur on bases adjacent to an existing repeat. Note that two additional transitions result from such mutations: fusion of nearby repeats (merging of two shorter repeats to form a longer repeat) and generation of new 'repeats' of length L=1 (included in our description for completeness, despite the lack of repetition). The distinction between these processes can be categorized by the number of B bases adjacent to the mutated site, one, zero, and two, for lengthening, fusion, and generation, respectively. The per-target rate mu is assumed to be the same constant for any repeat length L such that the per-repeat rate of these substitutions is necessarily linear in length, scaling as $\mu \times L$ for all repeat lengths.

2. *Shortening substitutions* ν: Point mutations $A \to B$ that decrease repeat length while preserving genome length. Such mutations also result in repeat fission (interruption of a repeat that forms two smaller repeats) and repeat destruction (mutation of L=1 repeats, removing them from the distribution). Again, these transitions can be categorized by adjacency of the mutated base to one, zero, and two B bases for repeat shortening, fission, and destruction, respectively. The per-repeat rate of shortening substitutions is again linear in repeat length: $\nu \times L$.

3. *Contractions* κ_L: Deletions of a single A base that decrease repeat length and genome length by one unit (i.e., one nucleotide for mononucleotide repeats). Extended deletions of two or more bases are assumed to be subdominant for simplicity (consistent with estimates provided in **Figure 3a** and **Supplementary Figure 4**). In the multiplier model (see **Table 1**, Equation S1), repeat contractions occur at a length-dependent rate constrained by empirical estimates and further parameterized by two variables; all rates for lengths $L \leq 8$ are fixed by trio-based rate estimates (see **Methods** and **Figure 3b**), the rate at $L = 9$ is determined by a constant multiple m relative to the value at $L = 8$ (i.e., $\kappa_{L=9} = m \times \kappa_{L=8}$), and rates for $L > 9$ follow a power law dependence of the form $\kappa_{L>9} = C_\kappa L^{\tau_\kappa}$. Here, the length-independent constant C_κ sets the initial value of the power law that parameterizes the contraction rate for all $L \geq 9$ (for intuition, if the power law

$C_\kappa L^{\tau_\kappa}$ was artificially extended below $L = 9$, C_κ would be the rate associated with $L = 1$: $C_\kappa L^{\tau_\epsilon}|_{L\to 1} = C_\kappa$); the subscript c refers to the crossover in behavior between substitution-dominated mutation rates at lengths $L < 9$ and the onset of repeat instability-dominated rates for $L > 9$. Under this parameterization, solving for $\kappa_9 = C_\kappa L^{\tau_\kappa}|_{L=9} = m\ \kappa_8$ yields the definition $C_\kappa = \kappa_{L=8} \times m/9^{\tau_\kappa}$, which is dependent on both τ_κ and m, but constant for a fixed parameter combination $(m, \tau_\epsilon, \tau_\kappa)$. The per-repeat target size for contractions in a repeat of length L is simply L, as the deletion of any base in an A repeat has an equivalent effect. Thus, the per-repeat rates are given by multiplying κ_L by an additional factor of L (i.e., the per-repeat contraction rate becomes $\kappa_L L = C_\kappa L^{1+\tau_\kappa}$ for long repeats $L \geq 9$). For all length-dependent rates (e.g., κ_L), we use the subscript L to denote the length dependence for both a discrete list of rates (e.g, κ_L defined in the discrete range of positive definite integers $L \in \mathbb{Z}^+$) and in the continuum where these rates are interpreted as continuous functions (e.g., $\kappa_L \to \kappa(L)$ becomes a continuous function describing the contraction rate in the continuous space $L \in \mathbb{R}^+$, but we preserve the notation $\kappa_L \equiv \kappa(L)$ in this case for consistency).

4 *Expansions* ϵ_L: Insertions of a single A base that increase repeat length and genome length by one unit. Again, extended insertions are assumed to occur at subdominant rates (see **Figure 3a** and **Supplementary Figure 4**). Expansions are defined to be dependent on length in the same way as contractions, defining low lengths empirically and including the same value for the parameter m, but with a distinct power law for rates at large lengths: $\epsilon_{L>9} = C_\epsilon L^{\tau_\epsilon}$. We have again expressed this dependence using the length-independent constant $C_\epsilon = \epsilon_8 \times m/9^{\tau_\epsilon}$ for notational convenience. We envision insertion as the replacement of a single A base with a pair of bases AA such that the per-target lengths are again multiplied by the target size L (e.g., asymptotically, $\epsilon_L L = C_\epsilon L^{1+\tau_\epsilon}$).

5 *Non-motif insertions* ι_L: Insertions of a single B base that interrupts a repeat, resulting in repeat fission. For conciseness, non-motif insertions will simply be referred to as *insertions* herein (with the implicit distinction between insertions leading to expansions and non-motif insertions). Extended insertions of more than a single base are again ignored (see **Figure 3a**, **Supplementary Figure 4**). The per-target rate of insertions is again length dependent in the same way, with $\iota_{L\leq 8}$ derived from empirical data, with the parameterization-dependent $\iota_9 = m\iota_8$ defined using the same multiple m, and rates for lengths $L > 9$ defined by the power law $\iota_L = C_\iota L^{\tau_\iota}$. In this case, the per-repeat rate is dependent on a target size of $L - 1$ (B must be inserted between two A bases in the pre-mutated repeat and there are $L-1$ pairs of adjacent motifs providing targets for $AA \to ABA$). Herein, we will assume that the rate ι_L was estimated by collecting the average per-repeat rate of insertions and dividing by the length of the repeat L (i.e., as an effective per-target rate), as in **Methods**; however, if the per-target rate ι_L is estimated directly (i.e., by separately counting each $AA \to ABA$ event), one may resort to the approximation $L - 1 \approx L$ in the asymptotic large L regime, which is the only relevant length range for insertion-based processes due to their highly subdominant rates (relative to, e.g., the expansion or contraction rates in the same regime). We make one additional assumption here to limit the number of free parameters in the multiplier model: with empirical motivation (see manuscript text), we impose the constraint $\tau_\iota = \tau_\epsilon$ under the assumption that they are both consequences of the same biological mechanisms such that the rates scale in parallel as L^{τ_ϵ}, albeit with non-motif insertions occurring at significantly lower rates. Thus, the per-repeat rate of insertions in this model is given by $\iota_L L = C_\iota L^{1+\tau_\iota} = C_\iota L^{1+\tau_\epsilon}$ (i.e., multiplying ι_L by L, rather than $L-1$, to recover the estimated per-repeat rate). As before, this is expressed in terms of the length-independent constant $C_\iota = \iota_{L=8} \times m/9^{\tau_\epsilon}$, where we have substituted in $\tau_\iota = \tau_\epsilon$.

All additional mutational processes and their impact on the repeat length distribution are assumed to be subdominant or ignored for analytic simplicity. For example, deletions of B bases are ignored throughout our analysis despite potential relevance to fusion rates, as the direct estimates of the deletion rate for length one B strings showed an orders of magnitude suppression relative to the substitution rate μ (a directly competing process resulting in repeat fusion; see **Methods**). We make no attempt to model the correlated dynamics of the B string length distribution such that insertions of B bases that lengthen B strings are also ignored, along with the insertion or deletion of extended sequences (i.e., $L \to L \pm k$, where $k > 1$; see above). With the exception of extended insertions and deletions, these effects were included in the results of our computational model at appropriate length-independent rates (e.g., the rate of $B \to BB$ was assumed to be the same as the rate of $BB \to BBB$, ignoring potential repeat instability due to the indistinguishability between $B \in \{C, T, G\}$), but do not change the qualitative behavior of the repeat length distribution. For the multiplier-coupled model, we assume the multiplier parameter m is positive definite and the exponent parameters τ_κ and τ_ϵ are positive semidefinite, which together imply that $C_\kappa, C_\epsilon, C_\iota > 0$.

1.2 Summary of the three-parameter multiplier-coupled model (for long repeats)

For the reader's reference, we summarize the mutation rate parameterization under the three-parameter multiplier-coupled model specified in **Table 1** for each mutation type. Repeat lengths below $L = 9$ units are taken directly from empirical point estimates at each length (see **Figure 3b**) calculated for expansion, contraction, and insertions. Substitution rates μ and ν are taken from motif-dependent empirical estimates and assumed to be length independent. For repeat length $L \geq 9$, the per-target rates for expansion (ϵ_L), contraction (κ_L), and insertion (ι_L) are parameterized as follows.

$$\begin{aligned}\epsilon_L &= C_\epsilon L^{\tau_\epsilon} \equiv \epsilon_8 m \left(\frac{L}{9}\right)^{\tau_\epsilon} \\ \kappa_L &= C_\kappa L^{\tau_\kappa} \equiv \kappa_8 m \left(\frac{L}{9}\right)^{\tau_\kappa} \\ \iota_L &= C_\iota L^{\tau_\epsilon} \equiv \iota_8 m \left(\frac{L}{9}\right)^{\tau_\epsilon}\end{aligned} \tag{S1}$$

The parameter-dependent constants C_ϵ, C_κ, and C_ι are computed from ϵ_8, κ_8, and ι_8, respectively, the empirical estimates at $L = 8$. The dependence on the number nine is a result of the largest length with reliable empirical estimates; for the purposes of the following analysis it is only important that this number is of order 10. Per-repeat rates are computed by multiplying each per-target rate by repeat length L (e.g., $\epsilon_L L$ for expansions, etc.). Noting that we have made the simplifying assumption that insertions and expansions obey the same power law (i.e., $\tau_\iota = \tau_\epsilon$), this provides a three-parameter description of the unobserved length dependencies of the mutation rates in terms of $(m, \tau_\epsilon, \tau_\kappa)$. This parameterization was used in our computational model (along with other models specified in **Table 1**) to propagate the mutational process over time and substituted into more general analytic expressions below for direct comparison.

Note that it may also be of interest to consider the more general four-parameter decoupled power-law model (see **Table 1**) by using the left-side definitions in Equation S1 (i.e., independent constants C_ϵ and C_κ, with $C_\iota = C_\epsilon/100$); where possible, results that require parameterization have been expressed in terms of these more generic constants for this purpose.

1.3 Mutational processes as transitions in length space (local vs. nonlocal)

Having defined each mutational process, we note that the location of the same type of mutation along the repeat sequence can result in dramatically different effects, as alluded to above. The catalogue of changes to repeat length can be separated into ***local*** transitions and ***nonlocal*** transitions (see schematic in **Figure 2b**), which provides an important distinction in the continuum approximation described below. Local transitions result from mutations that increase or decrease length by one motif unit, while nonlocal transitions change length by more than one unit.

Local transtions: $L \to L \pm 1$ (e.g., expansion, contraction)
Nonlocal transitions: $L \to L \pm k$ for $k > 1$ (e.g., fission, fusion)

While expansion is a strictly local processes in repeat length ($L \to L+1$) with probabilities associated with $\mathrm{P}_L \to \mathrm{P}_{L+1}$ transitions, μ substitutions are local *only* when they occur at sites adjacent to exactly one repeat boundary (e.g., $BBAB \to BAAB$, rather than $ABAB \to AAAB$). In contrast, μ substitution of a base initially adjacent to two distinct repeats results in an inherently nonlocal process: two repeats (e.g., length L_1 and L_2) are fused into a single longer repeat of length $L_1 + L_2 + 1$. This transition is both nonlocal in length space and non-conservative with respect to the mass of the probability distribution P(L). Substitutions at bases not adjacent to repeats generate new $L = 1$ 'repeats' and do not conserve mass (effectively a 'source' at the $L = 1$ boundary). This is counteracted by ν substitutions (with a distinct rate) that remove $L = 1$ repeats (effectively, a 'sink' at the same boundary). Analogously, repeat fusion is counteracted by repeat fission due to ν substitutions that occur in the non-boundary bases (i.e., in the 'body') of the repeat; these are again nonlocal in length space and non-conservative in distribution mass.

Fission has a target size linear in the length of the initial repeat (strictly, $L - 2$, ignoring the boundaries). In contrast, fusion has a target dependent on the number of B strings of length one. Local decreases in length can occur due to contraction (at a per-repeat rate *at least* linear in the length) or via ν substitutions (at a rate 2ν corresponding to the finite target size of two bases for repeats $L \geq 2$). Additional fission events can occur due to insertions. As noted above, additional fusions due to deletions of B bases are ignored due to negligible rates.

The nonlocal, non-conservative, and non-linear (e.g., power law dependencies and fusion rates dependent on two distinct length classes) nature of this ensemble of dynamics makes the difficulty of simultaneously modeling these effects immediately clear. Instead, motivated by the questions surrounding the maintenance and prevalence of long (and potentially disease-causing) repeats in the genome, we proceed with an analysis of the asymptotic dynamics. This approach provides a more straightforward understanding of the dominant processes appropriate in long repeat regime (i.e., $L \gg 1$) and the contrast to forces shaping the short repeat regime (roughly defined here as $L < 10$). We first focus on an analytic description of the distribution at short lengths due to its recognizable shape and relative simplicity.

2 Finite difference equation for repeat length changes

We first modeled the dynamics of repeat length changes by writing a discretized finite difference equation that describes the combined effects of all five mutational processes on the distribution $\mathrm{P}_L(t)$ as it evolves over time.

$$\mathrm{P}_L(t+1) \approx \mathrm{P}_L(t) + \Delta_\epsilon \mathrm{P}_L(t) + \Delta_\kappa \mathrm{P}_L(t) + \Delta_\iota \mathrm{P}_L(t) + \Delta_\mu \mathrm{P}_L(t) + \Delta_\nu \mathrm{P}_L(t) \tag{S2}$$

Here, we have assumed that all mutation rates are sufficiently small that the mutational processes can be linearized (i.e., the rate of multiple mutations on the same repeat in a single generation is negligibly small). This equation describes the discrete distribution at time $t+1$ as it evolves from the distribution at the previous time step due to changes induced by expansion, contraction, insertion, and substitutions (from both μ and ν mutations), respectively. This can be rearranged to express the change to the distribution for each length bin L in a single generation.

$$\Delta_t \mathrm{P}_L \approx (\Delta_\epsilon + \Delta_\kappa + \Delta_\iota + \Delta_\mu + \Delta_\nu)\mathrm{P}_L \tag{S3}$$

Each term in this expression is defined by the single-generation effect of a given mutational type on the number of length L repeats P_L.

2.1 Changes in repeat length due to expansion, contraction, and insertion

Expansions introduce single-unit changes in length ($L \to L+1$), independent of their location within the repeat (target size L, as defined above). The change in the number of repeats in the Lth class can be written as a combination of an influx due to insertion from below ($L-1 \to L$) and an outflux due to expansion to the class above ($L \to L+1$), as follows.

$$\Delta_\epsilon \mathrm{P}_L = \epsilon_{L-1}(L-1)\mathrm{P}_{L-1} - \epsilon_L L \mathrm{P}_L \tag{S4}$$

As defined above, ϵ_L represents the length-dependent expansion rate, which dramatically and monotonically increases above lengths of order ten. Contractions have the opposite effects: an influx from above ($L+1 \to L$) and an outflux to below ($L \to L-1$), with a length-dependent mutation rate κ_L and target size L.

$$\Delta_\kappa \mathrm{P}_L = \kappa_{L+1}(L+1)\mathrm{P}_{L+1} - \kappa_L L \mathrm{P}_L \tag{S5}$$

Importantly, we have omitted the effect of contraction-based repeat fusion, under the assumption that this process is subdominant to substitution-based fusion at all repeat lengths (see description of substitution-based fusion, below). (Non-motif) insertions are described by more complicated transitions, due to the position-dependent effects on length. Additionally, insertions are non-conservative, replacing one repeat with two repeats of shorter length ($L \to k, L-k$, where $k \geq 1$). The outflux due to insertions occurs at a length-dependent rate ι_L with target size L, which is a sum over the L possible locations for the insertion that result in distinguishable transitions (up to the symmetry $k \leftrightarrow L-k$; e.g., transitions $5 \to 4, 2$ are equivalent to $5 \to 2, 4$, despite distinguishable mutational targets). Note that, for the purposes of writing the finite difference equation, one need not keep track of the eventual state(s) resulting from an outflux. A focal class L can gain counts due to insertions in any repeat longer than L: insertions that occur L units away from either repeat boundary result in an increase in P_L, such that each length class $l > L$ has 2 potential targets for transitions $l \to L, l-L$ (in the case where $l = 2L$, there is a single target, but two length L repeats are added to P_L). Together, the effects of insertion on a focal L class can be written as follows.

$$\Delta_\iota \mathrm{P}_L = -\iota_L L \mathrm{P}_L + 2\sum_{l=L+1}^{\infty} \iota_l \mathrm{P}_l \tag{S6}$$

Here, we assume that P_L decays sufficiently rapidly as L increases such that $\sum_l \iota_l \mathrm{P}_l$ remains finite and P_L is normalizable. This sum characterizes repeat fission due to insertions alone, which are inherently nonlocal and

non-conservative transitions. However, note that contributions from transitions $L+1 \to L$ are local (though non-conservative) effects included in the same contribution. In this sense, this sum can be considered a combination of local transitions $L+1 \to L$ (contributing $2\iota_{L+1}\mathrm{P}_{L+1}$) and nonlocal transitions $l \geq L+2 \to L$ (contributing $2\sum_{l=L+2}^{\infty}\iota_l \mathrm{P}_l$); this decomposition is relevant only when insertion rates are appreciable at long repeat length, which we approximate in the continuum limit (detailed below). Similarly, the target size L in the outflux in Equation S6 is a sum of targets for local and nonlocal transitions (with targets 2 and $L-2$, respectively).

2.2 Changes in repeat length due to substitutions

Substitutions behave distinctly from the above transitions, though some aspects of the transitions they induce are analogous. For example, the outflux due to shortening substitutions is analogous to insertions (with target size L), but with a length-independent rate ν; this combines substitutions at the boundary that result in local transitions $L \to L-1$ (with target 2) and substitutions in the repeat body (target $L-2$) that result in repeat fission. The influx due to ν substitutions is again analogous to insertions and can be represented by a sum over contributions from local transitions $L+1 \to L$ and from all fissions of repeats of length $l \geq L+2 \to L$.

$$\Delta_\nu \mathrm{P}_L = -\nu L \mathrm{P}_L + 2\nu \mathrm{P}_{L+1} + 2\nu \sum_{l=L+2}^{\infty} \mathrm{P}_l = -\nu L \mathrm{P}_L + 2\nu \sum_{l=L+1}^{\infty} \mathrm{P}_l \tag{S7}$$

Again, we assume $\sum \mathrm{P}_l$ rapidly converges to a finite value such that P_L is normalizable, though this condition is weaker than for insertions due to the (naively) monotonic increase in the per-target rate ι_L.

Length-increasing substitutions with length-independent rate μ per target result in an outflux restricted to sites adjacent to the repeat boundary (i.e., μ mutations $B \to A$ at either end of $BA...AB$). This includes local transitions when the mutated B is initially adjacent to less than two A bases (e.g., $BBA \to BAA$ for transitions $L \to L+1$ or $BBB \to BAB$ for transitions $L = 0 \to 1$) and nonlocal transitions when the mutated base results in repeat fusion ($ABA \to AAA$). Here, the total target size is 2 and the net outflux is independent of the resulting repeat length(s).

The influx due to μ substitutions has distinct contributions from local transitions ($L-1 \to L$) and nonlocal fusion of shorter repeats ($L_1, L_2 \to L$, where $L_1 + L_2 = L-1$). For the purposes of describing the discrete dynamics, we can introduce a time-dependent constant $p_F(t)$ that represents the probability that mutation of a B base terminating a repeat results in repeat fusion, rather than a local transition; $p_F(t)$ (henceforth, the time-dependence of p_F will be left implicit for brevity) is dependent on the genomic distribution of B string lengths at time t (i.e., the fraction of B strings of length one) or, alternatively, the probability that the three-unit context of the mutated B results in a substitution $ABA \to AAA$ (i.e., the probability that the mutated B is adjacent to two A repeats). The rate of substitution-based repeat fusions is therefore proportional to μp_F and the rate of local length-increasing substitutions is proportional to $\mu(1-p_F)$. The quantity p_F approaches a time-independent constant in steady state and can be measured empirically from the B string length distribution, computed from other genome-wide quantities like the length of the genome and total number of repeats, or computed theoretically in simple cases (see discussion below), but is ultimately unimportant to our analysis and results. The combined effects of μ mutations on repeats of length L can be characterized as follows.

$$\Delta_\mu \mathrm{P}_L = -2\mu \mathrm{P}_L + 2\mu(1-p_F)\mathrm{P}_{L-1} + \mu p_F \sum_{l=1}^{L-2} \mathrm{P}_l \mathrm{P}_{L-1-l} \tag{S8}$$

The sum in the rightmost term is the discrete form of a convolution of the distribution P_L with itself, which has an intuitive interpretation. For integers distributed according to the discrete probability distribution P_L (assuming P_L is properly normalized), the probability of randomly sampling two integers $L_1, L_2 (< L-1)$ that sum to a fixed value $L-1$ is given by the convolution $\sum_{l=1}^{L-2} \mathrm{P}_l \mathrm{P}_{L-1-l}$; a substitution adds one unit to form a repeat of length L. Here, we have assumed that distinct classes of P_L remain uncorrelated in steady state for simplicity. This may be an oversimplification of the true steady state because, for example, fusion events may preferentially reverse repeat fission (i.e., fusion can re-form the original length of an interrupted repeat generated by fission; the length-dependent per-target rate of insertion-based interruptions can result in correlation between length classes, further complicating the dynamics).

We note that there can be a similar contribution that originates from deletions of a B adjacent to two repeats (i.e., deletion-based repeat fusion). However, based on the estimated mutation rates (see Methods), background deletions are orders of magnitude less frequent than μ substitutions such that deletion-induced fusion transitions occur at negligible rates.

2.3 Finite difference equation

Summing the above contributions to $\Delta \mathrm{P}_L$ yields the full finite difference equation describing the change in each length class P_L subject to two-way substitutions, expansions, contractions, and insertions.

$$\Delta_t \mathrm{P}_L = -(\nu L + 2\mu)\,\mathrm{P}_L + 2\nu \sum_{l=L+1}^{\infty} \mathrm{P}_l + 2\mu(1-p_F)\mathrm{P}_{L-1} + \mu p_F \sum_{l=1}^{L-2} \mathrm{P}_l \mathrm{P}_{L-1-l}$$
$$-(\epsilon_L + \kappa_L + \iota_L)\,L\mathrm{P}_L + \epsilon_{L-1}(L-1)\mathrm{P}_{L-1} + \kappa_{L+1}(L+1)\mathrm{P}_{L+1} + 2\sum_{l=L+1}^{\infty} \iota_l \mathrm{P}_l \tag{S9}$$

For clarity, the first line of terms summarizes changes due to substitution and the second summarizes insertion- and deletion-based changes due to repeat instability. Inclusion of deletion-based fusion and/or correlations between length classes, both of which are treated as negligible, would add an additional convolution and/or a dependence on the covariance between length classes, respectively.

Guided by striking differences in the length-dependencies of substitutions and repeat instability rates (**Figure 3b**), we further analyze the steady state dynamics by separating into distinct length regimes in which a subset of mutational processes dictate the vast majority of changes in length. Under this separation of length scales, the dynamics of short repeats (roughly, $L \le 8$ for mononucleotide-A repeats) and long repeats (roughly $L > 10$) are largely dominated by distinct mutational forces: the distribution of short repeats is maintained in a dominant balance between the opposing effects of μ and ν substitutions (including both fission and fusion), while length changes to long repeats are dominated by a (parameter-dependent) balance between expansions, contractions, and fission (potentially including fission resulting from ν substitutions). The assumption of dominant balance allows for a dramatic simplification of the full set of contributions to Equation S9; due to their low rates, all neglected terms provide minor corrections to the resulting approximation to the steady state distribution, which we demonstrate post-hoc.

Equation S9 represents the deterministic change of the repeat length distribution in a reference sequence (i.e., a single individual) due to mutations alone. Here, we have assumed that natural selection is absent such that the accumulation of mutations in the reference results from mutations aggregating along the lineage ancestral to this individual. This process occurs over sufficiently long times that a steady state is eventually reached. Once in steady state, the time-averaged distribution of repeat lengths (i.e., averaging over stochastic behavior) can be equated to the steady state distribution obtained from Equation S9.

3 Steady-state dynamics in the short repeat length regime

In contrast to previous studies, the empirical distribution we constructed (see **Figure 1b**) includes very short contiguous sequences and, notably, single base counts for comparison. The relative rates of expansions, contractions, and insertions to both types of substitutions makes it clear that repeat instability is are largely irrelevant to the maintenance of such short sequences (see **Figure 3b**). From a biological standpoint, this implies that repeat instability is technically irrelevant (i.e., occurs at highly suppressed rates) until repeats exceed roughly $L = 8$ (this differs slightly between motifs of distinct lengths and is more accurately described as roughly 6-12 nucleotides, rather than repeat units, for motifs of unit length $l_m \le 3$ nucleotides; see **Supplementary Figure 22**); this corresponds to the length range where expansion and contraction rates are comparable to substitution rates such that repeat instability becomes relevant to the dynamics.

The shape the distribution of short repeats is well-approximated as a balance between substitutions alone (see, e.g., black vs. dashed blue lines in **Figure 6**). The distribution is generated by the random process of sequence evolution under two-way substitution with distinct rates μ and ν. Given indefinite time, this process equilibrates to a steady state genome in which the probability of randomly sampling an A base is given by the fraction $p_A = \mu/(\mu+\nu)$ and the complementary probability of sampling a B base is given by $p_B = 1 - p_A = \nu/(\mu+\nu)$. Here, the relevant substitution rates μ and ν are the single-unit context rates (i.e., averaged over longer contexts) $\mu_{B\to A}$ and $\mu_{B\to A}$, respectively, as this distribution is not characterized by distinctions between local transitions, fission, and fusion. Under this model, a repeat is simply a contiguous sequence of A bases that is L bases (or repeated units) long and adjacent to a B base on either side. Conditioning on an initial B base, a length L string of A bases occurs at a frequency approximately given by the probability of randomly sampling an A base L successive times, followed by a terminating B base. The frequency of a length L repeat is therefore given by geometrically distributed distribution proportional to ${p_A}^L p_B$.

$$\mathrm{P}_L = \mathcal{N}\left(\frac{\mu}{\mu+\nu}\right)^L \left(\frac{\nu}{\mu+\nu}\right) \propto \left(\frac{\mu}{\mu+\nu}\right)^L \tag{S10}$$

Here, $\mathcal{N}$ is a normalization constant defined as $\mathcal{N} = 1/\sum_{L=1}^{\infty} \mathrm{P}_L$, where $\sum_L \mathrm{P}_L$ is the total mass of the distribution. As the steady state distribution is no longer geometrically distributed when repeat instability becomes relevant (roughly above $L = 8$), the value of $\mathcal{N}$ cannot be determined by the short repeat dynamics alone; the normalization can only be evaluated after identifying the steady state distribution over all length classes. Given that the constant $\mathcal{N}$ is unknown, the constant probability associated with sampling repeat-terminating B bases (i.e., the factor of p_B) can be absorbed into the definition of $\mathcal{N}$.

3.1 Geometric solution to the substitution-only difference equation

To demonstrate the utility of the finite difference equation in a simpler setting (without repeat instability), one can show that the geometric distribution, when normalized, provides the solution to the short length approximation to Equation S9 in steady state. Under substitutions alone, the short length regime is well-approximated by the following difference equation.

$$\Delta \mathrm{P}_L \approx -\nu L \mathrm{P}_L - 2\mu \mathrm{P}_L + 2\nu \sum_{k=L+1}^{\infty} \mathrm{P}_k + 2\mu(1-p_F)\mathrm{P}_{L-1} + \mu p_F \sum_{k=1}^{L-2} \mathrm{P}_k \mathrm{P}_{L-1-k} = 0 \tag{S11}$$

The finite difference equation becomes a steady state condition by imposing time independence (i.e., $\Delta \mathrm{P}_L = 0$). For consistency with Equations S7 and S8, the local influx from ν substitutions is included in the sum representing fission and the parameter $p_F = (1 - q_F)$ will be defined below to represent the appropriate rate of fusion in the present context. The normalized geometric distribution is given by the following expression in terms of p_A and $p_B = 1 - p_A$, defined above.

$$\mathrm{P}_L = p_B {p_A}^{L-1} = \frac{\nu \mu^{L-1}}{(\mu+\nu)^L} \tag{S12}$$

Though fusion and fission are inherently non-conservative transitions that change the number of repeats in the distribution, we make the approximation that individual fission and fusion events do not significantly alter the normalization constant in steady state. The term representing fission is now the incomplete sum over a geometric series, which can be explicitly evaluated as follows.

$$2\nu \sum_{k=L+1}^{\infty} \mathrm{P}_k = 2\nu p_B \sum_{k=L+1}^{\infty} {p_A}^{L-1} = 2\nu p_B \frac{{p_A}^L}{p_B} = 2\mu \mathrm{P}_L \tag{S13}$$

Thus, the influx due to both repeat fission and local transitions, both ν substitution-based effects, exactly cancel the outlux due to μ mutations.

To evaluate the convolution term, note that the probability distribution for the length of B strings under two-way substitution $\tilde{\mathrm{P}}_\lambda$ is geometric by the same arguments as for the A distribution, but with reversed probabilities $p_A \leftrightarrow p_B$. Normalizing string lengths $\lambda = [1, \infty)$ (i.e., conditional on a B string of length $\lambda \geq 0$), the normalized probability distribution is as follows.

$$\tilde{\mathrm{P}}_\lambda = p_A {p_B}^{\lambda-1} \tag{S14}$$

The probability that any given A string is terminated by a B string of length $\lambda = 1$ is simply $\tilde{\mathrm{P}}_1 = p_A$. This determines the steady state probability of fusion p_F, for which a μ substitution in a length one B string results in the transition $L_1, L_2, \to L_1 + L_2 + 1$. The complement is the probability of locally increasing the length by a single unit (as opposed to fusion) due to an adjacent μ substitution, $q_F = 1 - \tilde{\mathrm{P}}_1 = 1 - p_A = p_B$. After cancelling the ν substitution influx with the μ substitution outflux, we can rewrite the remaining terms in Equation S11 by substituting in $p_F = p_A$, $q_F = p_B$ and evaluating the partial sum in the fusion term.

$$\begin{aligned} -\nu L \mathrm{P}_L + 2\mu q_F \mathrm{P}_{L-1} + \mu p_F \sum_{k=1}^{L-2} \mathrm{P}_k \mathrm{P}_{L-1-k} &= -\nu L \mathrm{P}_L + 2\mu p_B \mathrm{P}_{L-1} + \mu p_A {p_B}^2 \sum_{k=1}^{L-2} {p_A}^{k-1} {p_A}^{L-k-2} \\ &= -\nu L \mathrm{P}_L + 2\mu \frac{p_B}{p_A} \mathrm{P}_L + \mu p_A {p_B}^2 \sum_{k=1}^{L-2} {p_A}^{L-2} \\ &= -\nu L \mathrm{P}_L + 2\nu \mathrm{P}_L + \nu(L-2) p_B p_A {p_A}^{L-2} \\ &= -\nu(L-2)\mathrm{P}_L + \mu(L-2)\mathrm{P}_L = 0 \end{aligned} \tag{S15}$$

We find that a geometric distribution of both A and B repeats satisfies the steady state equation under two-way substitution, which justifies the aforementioned approximation for the substitution-dominated short length regime of the distribution.

3.2 Interactions between short and long repeats are largely restricted to boundary effects

Here, we are only concerned with the geometric falloff in Equation S10 that defines the shape of the distribution in this length regime, which is given by the proportionality on the right hand side of the above expression. Importantly, this length dependence remains largely independent of the dynamics of the long length regime, which only affects the normalization constant. This separation of the dynamics for short and long length repeats is a reasonable approximation because the rate of transitions between these regimes is low and primarily limited to the intermediate lengths at $L \sim 8$–10 (i.e., the boundary between the short and long repeat regimes). As discussed below, we allow for an influx from the long length regime but note that it is inherently negligible because the relative mass of short length repeats vastly outweighs the mass in the long repeat tail of the distribution. In this sense, the short repeat regime can be treated as a probability sink for the long repeat distribution for length transitions that exit the long length regime. Similarly, the mass in the long length regime is sourced by short repeats that approach the length regime boundary and are quickly subject to expansion-biased instability; this can be considered a local conditions on the boundary between the regimes without altering the dynamics elsewhere. If the steady state is in detailed balance (i.e., the flux in and out of each length class vanishes independently), fluxes at the length regime boundary must cancel such that both ends of the distribution maintain their relative weights.

While it is immediately clear that the distribution of A repeats deviates from a simple geometric decay due to the combined action of expansion, contraction, and insertion in the long length regime, the B string length distribution is unaltered by expansion and contraction, which solely manipulate A repeat length. While likely unrealistic, we proceeded under the assumption that B strings do not constitute repeats and are therefore not directly subject to repeat instability. Therefore, any deviation from a simple geometric distribution can only be due to the effects of insertion, which generates new length one B strings during each transition. Although the additional source of B strings will contribute to the total number of B strings in the eventual steady state balance between the A and B distributions, localization of this influx to the lowest length class (B strings of length $L = 1$) implies that the normalized distribution of B lengths remains approximately geometric.

4 Steady-state dynamics for asymptotically long repeat lengths

The steady-state distribution at long lengths has a qualitatively distinct shape from the short length regime, exhibiting a much heavier tail and slower decay than the geometric distribution. Although our de novo rate estimates do not cover the entirety of this range, we observed an initially rapid, monotonic increase in the per-target expansion and contraction rates with length; this must eventually reach the well-documented extreme rates observed in repeat expansion disorders. Assuming sustained, naively monotonic rate increases with length, per-target repeat instability rates must eventually exceed substitution rates due to the stronger dependence on length (this is already observable at lengths of order 10 in **Figure 3b**). Above some sufficiently long length, the instability-based length changes comprise the majority of mutations, dominating the mutational dynamics; this suggests that the long repeat regime of the steady-state DRL is shaped by the mutational processes with the strongest length dependencies. However, the point at which substitution-based fission (which scales linearly with repeat length) can be ignored is entirely parameter dependent. The forces relevant to the longest length repeats in the DRL are thus some subset of expansion, contraction, substitution-based fission, and insertion-based fission (or the combination of all four).

To disentangle the relative importance of each mutational process, we employed a large L continuum approximation to the finite difference equation to analyze the long-length dynamics. For long repeats, $L \gg 1$ such that the distance between length bins can be treated as infinitesimal; this allows for a continuous approximation to the discrete Equation S9 appropriate only for repeat lengths of at least order 10. The distinction between local and nonlocal terms becomes important in the continuum, as local differences are approximated by continuous derivatives, while the nonlocal sums in Equation S9 (i.e., mutational transitions from across the rest of the distribution) are approximated as integrals.

4.1 Local contributions to the change in P_L

We first focus on local changes to the length distribution due to each of the forces. Starting with expansion in Equation S4, the discrete change to a focal length class P_L is the difference between an influx due to $L - 1 \to L$ length-increasing transitions and an outflux due to $L \to L + 1$ length-increasing transitions. The strictly local nature of expansion allows for a local approximation to $\Delta_\epsilon \mathrm{P}_L$ using a Taylor expansion in the continuum. To elucidate the continuum limit, we treat expansions in detail, below, but note that the approximation for all local contributions can be obtained analogously. We first define the distance between discrete length classes $\Delta L = 1$, which suggests

that we must change units before formally taking the continuum limit and assessing the accuracy of the resulting approximation.

$$\begin{aligned}\Delta_\epsilon \mathrm{P}_L &= (L-1)\epsilon_{L-1}\mathrm{P}_{L-1} - L\epsilon_L \mathrm{P}_L \\ &= F_\epsilon(L-\Delta L) - F_\epsilon(L) \\ &= F_\epsilon(x-\Delta x) - F_\epsilon(x)\end{aligned} \tag{S16}$$

Here, ϵ_L is the length-dependence of the expansion rate (e.g., $\epsilon_L = C_\epsilon L^{\tau_\epsilon}$ for $L \geq 9$ under the power-law model in Equation S1). The function $F_\epsilon(L) = \epsilon_L L \mathrm{P}_L$ was defined solely for notational convenience. In the third line, repeat length L was rescaled under a change of variables defined by $x = L/c$ (with $\Delta x = \Delta L/c = 1/c$); c is an arbitrary dimensionful constant with the same units as L (i.e., measured in a number of motif units) such that x is dimensionless. If x is kept fixed while L grows large, Δx gets increasingly smaller. Thus, choosing an appropriate c allows the backward finite difference $\Delta F_\epsilon(x) = F_\epsilon(x-\Delta x) - F_\epsilon(x)$ to be approximated by a Taylor expansion in small Δx truncated at a some finite order (for all practical purposes, this is equivalent to a Taylor expansion around 'small' ΔL after setting $\Delta L = 1$ when $L \gg 1$, but side steps the concern that $\Delta L = 1$ motif unit is definitionally not infinitesimal). Taking the continuum limit $L \to \infty$ and $\Delta x \to 0$, F_ϵ becomes the continuous function f_ϵ and the discrete derivative approaches the continuous derivative $\Delta F_\epsilon \to \partial_x f_\epsilon$. For finite L (and thus finite c), the first-order continuum approximation is accurate to order Δx^2.

$$\Delta_\epsilon \mathrm{P}_L = \frac{\partial x}{\partial L}\frac{\partial f_\epsilon(x)}{\partial x} + \mathcal{O}(\Delta x^2) \approx -\partial_L\left[\epsilon_L L \rho(L)\right] \tag{S17}$$

This is expressed in terms of a general, length-dependent expansion rate ϵ_L (at sufficiently long lengths) to allow for generic parameterizations, including Equation S1. To arrive at this approximation, the variable change was inverted (i.e., imposing $L = cx$, where $\partial L/\partial x = 1/c$; the chain rule is explicit in the middle expression for clarity) after taking the continuum approximation. Here, the notation ∂_x^n represents the nth derivative with respect to x and will be used henceforth for brevity. In the length continuum, we assume that the discrete distribution P_L can be well-approximated by the continuous, differentiable function $\rho(L)$ (defined in the rescaled length units $\rho(x) \equiv \lim_{\Delta x\to 0} P(x)$) such that the derivative $\partial_L \rho(L)$ represents the flux through length L. The continuum approximation is only applicable for $L \gg 1$ and all expressions dependent on $\rho(L)$ should be considered implicitly conditioned on $L \gtrsim 10$ such that the long-length scaling behavior of the mutation rates can be used directly (i.e., empirically-derived rates for $L < 9$ can be ignored such that length scaling for each rate is dictated by the parameterization, e.g., Equation S1). Importantly, at finite L, corrections to the lowest-order approximation to the finite difference $\Delta F(L)$ for a discrete function $F(L)$ cannot be treated as negligible when $\partial_L f(L)$ vanishes (see discussion of second-order corrections, below). Equation S17 describes a strictly local transition that drives a length-dependent flux through the focal length L, which represents the collective impact of expansion rates on the distribution; if desired, the chain rule can be applied to show a separate flux and length-dependent loss of mass due to expansion (i.e., $\Delta_\epsilon \mathrm{P}_L \approx -\rho(L)\partial_L(\epsilon_L L) - \epsilon_L L \partial_L \rho(L)$). Noting that the derivative is negative throughout the roughly monotonic empirical distribution, the flux due to expansion may describe an overall increase or decrease in length, depending on the relative magnitude of the non-derivative contribution.

The effects of contraction $\Delta_\kappa \mathrm{P}_L$ can be summarized analogously in terms of contributions to and from the adjacent shorter and longer length bins, respectively, which becomes a local length-dependent flux in the opposite direction in the continuum limit. This sign difference emerges because the continuum approximation is to a forward finite difference for contractions and to a backward finite difference for expansions (i.e., the difference between continuum approximations for $L+1 \to L$ and $L-1 \to L$ transitions).

$$\Delta_\kappa \mathrm{P}_L = (L+1)\kappa_{L+1}\mathrm{P}_{L+1} - L\kappa_L \mathrm{P}_L \approx \partial_L\left(\kappa_L L \mathrm{P}_L\right) \tag{S18}$$

Like expansions, contractions amount only to local changes to the distribution, but the length dependence of the per-repeat rate remains important to our understanding of the dynamics.

In contrast, substitutions induce both local and nonlocal transitions. We first isolate the local transitions from in Equation S7 and S8, treating the nonlocal contributions as fissions and fusions (see below). In discrete form, the local influx and outflux towards increasing length due to μ substitutions and towards decreasing length due to ν substitutions can be represented as follows.

$$\left(\Delta_\mu + \Delta_\nu\right)_{local} \mathrm{P}_L = 2\mu(1-p_F)\left(\mathrm{P}_{L-1} - \mathrm{P}_L\right) + 2\nu\left(\mathrm{P}_{L+1} - \mathrm{P}_L\right) \tag{S19}$$

Unlike expansion and contraction, the target size for local changes in length due to substitution is a length-independent factor of 2 associated with mutations at either boundary (e.g., $AAA \to AAB$ and $\to BAA$ for ν substitutions; μ substitutions reverse these transitions), which have a distinct effect from mutations at non-boundary loci. The target size for μ substitutions is reduced further because a fraction p_F transitions on the boundary result in fusion, rather than local increases in length (i.e., leaving a target of $2(1-p_F) = 2q_F$ per repeat). The first-order continuum approximation to Equation S19 is the following.

$$(\Delta_\mu + \Delta_\nu)_{local} \mathrm{P}_L \approx -\partial_L \left(2\left(\mu(1-p_F) - \nu\right)\rho(L)\right) \tag{S20}$$

Insertions include a similar local term, but this comes with an additional complication. If an insertion occurs adjacent to either boundary (with target size 2: $AA...AA \to ABA...AA$ and $\to AA...ABA$), the resulting repeat fission desribes the replacement of one length L repeat with one length $L-1$ repeat and one length 1 'repeat' ($L \to L-1, 1$). This is the only example of repeat fission that also contains a local transition, but will not be explicitly accounted for in our treatment of insertion-based fission below. The local component of this contribution can be written as follows.

$$(\Delta_\iota)_{local} \mathrm{P}_L = 2\iota_{L+1}\mathrm{P}_{L+1} - 2\iota_L \mathrm{P}_L \approx \partial_L \left(2\iota_L \rho(L)\right) \tag{S21}$$

Due to the limited target of two possible insertions resulting in this local transition, the appropriate length dependence inside the derivative is twice the per-target rate ι_L, rather than a dependence on the per-repeat insertion rate. The correlated nonlocal contribution to the $L = 1$ bin can be ignored because the relatively low rate of insertions results in an exceedingly small influx into $L = 1$ relative to the geometrically distributed mass maintained by substitutions.

4.1.1 Competition between first-order local effects

The length-independent (and modest) target size, matching power law exponent (assumed for all parameterizations), and globally suppressed rate of insertions relative to expansions (based on empirical estimation, where possible; see **Figure 3b**) together ensure that local insertions remain subdominant to expansions (i.e., $(\Delta_\iota)_{local}\mathrm{P}_L \ll \Delta_\epsilon \mathrm{P}_L$ for any L); as a result, local insertions may be neglected entirely. By the same argument, and given that expansion and contraction rates far exceed the substitution rates at long lengths, local changes in length due to substitutions are likely negligible in the $L \gg 1$ regime of interest. Additionally, we will at this point treat time as continuous (i.e., assuming infinitesimal generation time after appropriately rescaling the units of all rates) such that the per-generation change in occupancy of P_L can be approximated by the time-derivative $\partial_t \rho(L)$ (note that we revert back to the notation $\Delta \mathrm{P}_L$ when referencing the discrete equations). Under these approximations, the first-order approximation to the local dynamics can be summarized by the following.

$$\begin{aligned}(\partial_t \rho(L))_{local} &\approx -\partial_L \left[\left((\epsilon_L - \kappa_L)L - 2\iota_L + 2(\mu(1-p_F) - \nu)\right)\rho(L)\right] \\ &\approx -\partial_L \left[\left((\epsilon_L - \kappa_L)L\right)\rho(L)\right]\end{aligned} \tag{S22}$$

The quantity $\epsilon_L - \kappa_L$ represents the bias between expansion and contraction at a given length; the lowest-order approximation to the local dynamics thus describes a flux through each length class due to the length-dependent expansion-contraction bias.

The sign difference between the expansion and contraction terms suggests that, if their magnitudes are nearly identical at a given length (i.e., $(\Delta_\epsilon + \Delta_\kappa)\mathrm{P}_l \approx 0$ such that length changes are nearly symmetric at length l), contributions from insertion and/or substitution, which subdominant otherwise, could become relevant (in principle). Under the somewhat simplistic multiplier-coupled parameterization in Equation S1, this can occur only at a single, specific (but parameter-dependent) length L^*.

$$L^* \equiv \left(\frac{C_\epsilon}{C_\kappa}\right)^{\frac{1}{\Delta\tau}} = 9\left(\frac{\epsilon_8}{\kappa_8}\right)^{\frac{1}{\Delta\tau}} \tag{S23}$$

We have expressed this in terms of the collapsed parameter $\Delta\tau$, the difference between the contraction and expansion exponents (referred to extensively below).

$$\Delta\tau \equiv \tau_\kappa - \tau_\epsilon \tag{S24}$$

L^* is independent of the multiplier m and dependent only on $\Delta\tau$, rather than on τ_ϵ and/or τ_κ individually. Note that the factor of 9 in Equation S23 is simply a consequence of defining the multiplier model in terms of the estimated rates

ϵ_8 and κ_8 (and qualitatively unimportant). While this quantity is notable because of the potential for subdominant local contributions (from insertion and substitution), second-order corrections to the finite difference equation (due to expansion and contraction) dramatically exceed these effects due to their scaling with target size. However, L^* also aids in qualitative interpretation because it describes the transition from expansion- to contraction-biased dynamics in this parameterization.

4.1.2 Second-order corrections to the local behavior and diffusive dynamics

When the first-order approximation to the local dynamics vanishes (due to nearly symmetric expansion an contraction rates across some range of lengths), subsequent corrections to the continuum approximation at finite L can dominate the leading-order local behavior. The computationally-explored parameter space (i.e., values of $(m, \tau_\epsilon, \tau_\kappa)$ in the multiplier model) included L^* values well-within the populated range of the long length tail of the DRL. In such cases, further corrections to the local behavior are necessary to characterize the dynamics in the continuum approximation.

With this in mind, we obtained the next-order approximation (i.e., the first strictly non-vanishing contribution) to Equation S9 by Taylor expanding the expressions for expansion, contraction, insertion, and substitution to second order in the continuum limit. For expansion, we approximate Equation S17 to order Δx^2 in the dimensionless variable x (i.e., the appropriately rescaled L) to produce the following approximation.

$$\begin{aligned} \Delta_\epsilon \mathrm{P}_L &= \left[f_\epsilon(x) - [\partial_x f_\epsilon(x)]\,\Delta x + \frac{1}{2}\left[\partial_x^2 f_\epsilon(x)\right]\Delta x^2 + \mathcal{O}(\Delta x^3) \right] - f_\epsilon(x) \\ &\approx -\partial_L\left[\epsilon_L \rho(L)\right] + \frac{1}{2}\partial_L^2\left[\epsilon_L \rho(L)\right] \end{aligned} \tag{S25}$$

All other mutational effects can be expanded to the same order analogously. Importantly, the second-order contribution is positive-definite for each mutational process such that their sum is non-vanishing (in contrast to the sign difference that allows the first-order term to vanish at L^*). This results in the following approximation to the continuous-time local dynamics in the large length regime.

$$(\partial_t \rho(L))_{local} \approx -\partial_L\left[\left((\epsilon_L - \kappa_L)L - 2\iota_L + 2\left(\mu(1-p_F) - \nu\right)\right)\rho(L)\right] + \frac{1}{2}\partial_L^2\left[\left((\epsilon_L + \kappa_L)L + 2\iota_L + 2(\mu(1-p_F) + \nu)\right)\rho(L)\right] \tag{S26}$$

This expression provides a continuum approximation for all *local* length transitions in terms of arbitrary, length-dependent mutation rates (estimated directly, or parameterized). Higher order corrections would only become necessary in the neighborhood of a length at which the first and second derivative terms both vanish (for example, in the unrealistic scenario where extrema in the length dependencies $L\epsilon_L\rho(L)$, $L\kappa_L\rho(L)$, $\iota_L\rho(L)$, and $\rho(L)$ occur at the same length). Using the multiplier model in Equation S1, the relative importance of each mutational process to local transitions can be assessed by contrasting their relative magnitudes and length dependencies.

$$\begin{aligned} (\partial_t \rho(L))_{local} \approx & -\partial_L\left[\left(C_\epsilon L^{1+\tau_\epsilon} - C_\kappa L^{1+\tau_\kappa} - 2C_\iota L^{\tau_\epsilon} + 2\left(\mu(1-p_F) - \nu\right)\right)\rho(L)\right] \\ & + \frac{1}{2}\partial_L^2\left[\left(C_\epsilon L^{1+\tau_\epsilon} + C_\kappa L^{1+\tau_\kappa} + 2C_\iota L^{\tau_\epsilon} + 2(\mu(1-p_F) + \nu)\right)\rho(L)\right] \end{aligned} \tag{S27}$$

The second derivative terms in this approximation can be collectively viewed as diffusion-like changes to repeat length. Unlike bias-driven fluxes from the first derivative, the second-order term represents symmetric, bidirectional transitions (i.e., equiprobable local increases and decreases in length). Due to the monotonic increase in instability rates, these diffusion-like effects grow stronger at longer lengths. Relative to expansions and contractions, substitutions and insertions contribute negligibly to the second derivative; both contributions have a finite target sizes and, despite increasing per-target rates, insertion occurs far less frequently than expansion at all lengths (i.e., $C_\iota \ll C_\epsilon$ such that $\iota_L \ll \epsilon_L$, independent of parameter values). The local dynamics are therefore well approximated by the following expression, dependent only on expansion and contraction.

$$(\partial_t \rho(L))_{local} \approx -\partial_L\left[\left(\epsilon_L - \kappa_L\right)L\rho(L)\right] + \frac{1}{2}\partial_L^2\left[\left(\epsilon_L + \kappa_L\right)L\rho(L)\right] \tag{S28}$$

This general form can again be evaluated under any parameterization (e.g., Equation S1) to approximate the ensemble of local transitions for long repeats, provided the assumption that expansion exceeds insertion remains valid. These local changes in length are a competition between bias-driven directional flux and diffusive changes in length, both generated primarily by expansion and contraction. Diffusive effects necessarily dominate when bias vanishes (e.g., in the neighborhood of L^*); at all other lengths, the relative importance of these effects is a parameter dependent competition between the net expansion-contraction bias (i.e., asymmetric component of instability) and the average magnitude of expansion and contraction (symmetric component).

4.2 Repeat fission as a nonlocal contribution to changes in length

In contrast to the local effects described above, repeat fission and fusion are inherently nonlocal processes. Fission is a consequence of interruptions due to insertions or ν substitutions that generates both a flux out of the focal L class (producing two shorter repeats) and a corresponding flux into the same class from fissions of longer repeats; the total number of repeats is altered by the fission of one contiguous repeat into two. Conceptual distinctions between substitution and insertion in this context are minor in the continuum: insertions allow transitions to and from adjacent length classes (e.g., $L \to L-1, 1$), while substitutions require transitions to and from at least two length bins away (e.g., $L \to L-2, 1$). Additionally, insertion within a repeat necessarily results in fission and does not conserve total genomic length (though, this does not *a priori* alter the mass of the A repeat distribution), unlike substitutions. The most pertinent difference owes to the length-dependent per-target rate of insertions, which can dramatically increase the number of such events as repeat length increases, despite initially much lower per-site rates at lower repeat lengths (see **Figure 3b**). In both cases, the nonlocal nature of fission-generated length transitions complicates the modeling of the dynamics, as the associated influx from all higher length bins amounts to a sum of distinct contributions to the change in P_L at each length (see Equations S6 and S7).

4.2.1 Substitution-based fission

Fission occurs only as a consequence of ν substitutions, with no dependence μ. After removing the target for local transitions at the repeat boundaries (see Equation S19), the remaining target for nonlocal transitions is $L-2$, corresponding to the body of the repeat. While the local transitions generate derivatives in the continuum (Equation S26), the remaining terms in Equation S7 result in an integral over the distribution of repeats longer than the focal class P_L.

$$\begin{aligned}(\Delta_\nu \mathrm{P}_L)_{fiss} &= -\nu(L-2)\mathrm{P}_L + \sum_{l=L+2}^{\infty} 2\nu \mathrm{P}_l \\ &\approx -\nu(L-2\Delta L)\rho(L) + 2\nu \int_{L+2\Delta L}^{\infty} \rho(\lambda)d\lambda \\ &\approx -\nu L\rho(L) + 2\nu \int_{L}^{\infty} \rho(\lambda)d\lambda \end{aligned} \tag{S29}$$

Here, the lower limit of the sum differs from Equation S7 after removing local transitions $L+1 \to L$. However, the continuum approximation applies only to the asymptotically long length regime where $L \gg \Delta L = 1$; in this limit, the approximations $L + 2\Delta L \approx L$ (integration limit) and $L - 2\Delta L \approx L$ (target size for outflux) were used to obtain the final line, above. The linear length scaling in the outflux represents the fact that any substitution in the repeat body alters the repeat length. In contrast, there are exactly two fission targets that generate transitions from each longer length class: specific substitutions L units away from either boundary of a length λ repeat result in a shorter repeat of length L (along with one of length $\lambda - L - 1$, which does not add to the length L bin). In the special case where $\lambda = 2L + 1$, there is only one relevant target for substitution (the middle base), but this event generates two length L repeats, rather than one. All longer repeats therefore contribute identically to the influx, independent of length, resulting in an integral over the distribution of longer length bins. The net direction of fission (i.e., influx minus outflux) depends on the focal length and the number of longer-length repeats in the distribution tail (i.e., how rapidly it decays and truncates).

4.2.2 Insertion-based fission

Fission due to insertions can be described analogously by taking the continuum limit of the nonlocal component of Equation S6 after removing the local transitions included in Equations S21 and S26. Note that, in this case, the length dependence of the per-target rate ι_L appears under the sum (and integral).

$$\begin{aligned}(\Delta_\iota \mathrm{P}_L)_{fiss} &= -(L-2)\iota_L \mathrm{P}_L + \sum_{l=L+2}^{\infty} 2\iota_l \mathrm{P}_l \\ &\approx -(L-2\Delta L)\iota_L\rho(L) + \int_{L+2\Delta L}^{\infty} 2\iota_\lambda \rho(\lambda)d\lambda \\ &\approx -L\iota_L\rho(L) + \int_{L}^{\infty} 2\iota_\lambda \rho(\lambda)d\lambda \end{aligned} \tag{S30}$$

Analogous to substitutions, the target size $L-2$ and summation limit $l = L+2$ refer only to nonlocal transitions away from the repeat boundary, both of which are approximately L in the $L \gg 1$ continuum. The resulting contributions

resemble Equation S29, but the length dependence of ι_L alters the length scalings (e.g., under the multiplier model, the outflux scales as $L^{1+\tau_\epsilon}$, rather than L). The influx is instead a weighted integral over the longer-length distribution tail; the net direction of fission therefore depends on the length dependence of ι_L (e.g., net fission in the multiplier model depends on the parameter τ_ϵ). This difference in overall length scalings can result in a competition between substitution and insertion that can transition from substitution-dominated to insertion-dominated fissions as the focal length is increased.

4.3 Repeat fusion under random sampling of the length distribution

Repeat fusion, the process by which either μ substitutions or, at a lower rate, deletions of length one B strings (henceforth *B-dels*) result in the merging of two shorter repeats into a longer repeat, substantially complicate our model of repeat length dynamics. For simplicity, we only describe μ substitution-based fusion (as shown in Equation S8), but note that differences between substitution- and B-del-generated fusion are entirely analogous to those between substitution- and insertion-generated fission. The only notable exception is that we found no evidence that the rate of deletions between repeats (namely, deletions of single-nucleotide or single-unit interruptions) harbor any dependence on the length of the repeat. Additionally, empirical estimates of *de novo* rates from trio data indicate that the per-target rate of B-dels (roughly 2×10^{-10} per generation) is suppressed relative to the per-target rate for μ substitutions (roughly 4×10^{-9} per generation; see **Methods**) by more than an order of magnitude, suggesting B-dels provide an entirely negligible correction to substitution-based fusion rates. Henceforth, all discussion of fusion is focused on substitution-generated events.

Fusion can only occur due to mutations at B sites immediately adjacent to two A sites and thus occur at a rate proportional to the fraction B strings of single-unit length p_F (see Equation S8 and subsequent discussion below Equation S14). In the absence of insertions, this probability is given by $p_F = \mu/(\mu+\nu)$ (expansions and contractions of A repeats do not alter this rate). Although insertions could alter this rate, their inclusion results in a (slightly) greater influx into the $L = 1$ class of B strings. This amounts only to an additional source at the low length boundary; while this alters the total number of B strings, the normalized probability distribution remains geometric (i.e., Equation S14) with a slightly modified rate constant. We confirmed via our computational model that this geometric distribution is nearly identical to that under two-way substitution alone, due to the overwhelming mass in the length one class and negligible influx due to the low overall insertion rates. However, the computational model is an abstraction that assumes B-strings are not susceptible to repeat instability. In practice, the value of p_F could be better estimated from the empirical distribution of B strings, but this was unnecessary in the current setting, as any such estimate does not impact our analysis or results.

We proceed by taking the continuum limit of Equation S8 after removing local contributions (i.e., those proportional to $(1-p_F)$).

$$
\begin{aligned}
(\Delta_\mu)_{fuse}\mathrm{P}_L &= -2\mu p_F \mathrm{P}_L + \mu p_F \sum_{k=1}^{L-2} \mathrm{P}_k \mathrm{P}_{L-1-k} \\
&\approx -2\mu p_F\ \rho(L) + \mu p_F \int_{\Delta L}^{L-2\Delta L} \rho(\lambda)\rho(L-\Delta L-\lambda)d\lambda \\
&\approx -2\mu p_F \rho(L) + \mu p_F \int_0^L \rho(\lambda)\rho(L-\lambda)d\lambda
\end{aligned}
\tag{S31}
$$

As written, the sum contains an implicit factor of two associated with swapping the subscripts $k \leftrightarrow L-1-k$ (i.e., double counting when summing up to $k = L-2$), which remains in integral form (i.e., $\lambda \leftrightarrow L-\lambda$ when integrating to L). The expression for fusion is explicitly nonlocal due to the quadratic, integral dependence on the distribution, which describes randomly sampling two shorter repeats of appropriate lengths. In the second line, we have again taken the large L asymptotic limit $L \gg \Delta L = 1$ to suppress subdominant terms. In this form, it becomes clear that the integral is simply a convolution of the distribution $\rho(L)$ with itself over a finite length window; this can be readily interpreted as the distribution of the sum of two random lengths drawn from the same probability distribution $\rho(L)$ (albeit not necessarily normalized appropriately due to non-conservative transitions). Noting that $p_F < 1$ and $\mu < \nu$ (from empirical estimates), the fusion outflux is strictly less than the outflux from substitution-based fission alone; this contribution becomes negligible in the long length regime due to the finite target size for fusions (i.e., $2\mu p_F \ll \nu L$). However, the integral terms cannot be analogously compared due to their distinct functional forms and non-overlapping limits.

Due to the substantial analytic complications introduced by repeat fusion, we turned to our computational model to quantify its effects across the long repeat regime. Using the three-parameter multiplier-coupled model (see Equation S1), we produced final-time point flux plots (e.g., those shown in **Figure 6c**) across the parameter space

to assess the relative effects of each class of transitions at each length. While fusion generated prominent transitions in the short repeat regime (maintaining the geometric decay detailed above), it remained negligible at longer lengths, regardless of parameter combination. We confirmed this observation by exploring parameters within the 95% HDR of our alternative parameterizations (see **Table 1**), which reinforced this observation. This suggested that, at least in steady state, the dominant dynamics in the long repeat regime remain largely unaffected by infrequent transitions due to repeat fusion.

4.4 Steady-state condition for long repeat dynamics

Collecting the continuum approximations to each term of Equation S9, the continuous-time dynamics of long repeats in the asymptotic large L regime can be described by the following partial differential equation (PDE).

$$\partial_t\rho(L) \approx \left\{\frac{1}{2}\partial_L^2\left[\left((\epsilon_L+\kappa_L)L+2\iota_L+2(\mu(1-p_F)+\nu)\right)\rho(L)\right]-\partial_L\left[\left((\epsilon_L-\kappa_L)L-2\iota_L+2(\mu(1-p_F)-\nu)\right)\rho(L)\right]\right\}_{local} + \left\{-(\nu+\iota_L)L\rho(L)+\int_L^\infty 2(\nu+\iota_\lambda)\rho(\lambda)d\lambda\right\}_{fiss} + \left\{-2\mu p_F\rho(L)+\mu p_F\int_0^L\rho(\lambda)\rho(L-\lambda)d\lambda\right\}_{fuse} \tag{S32}$$

The above expression is again left in terms of general length-dependent rates of expansion, contraction, and insertion and appropriately labeled to differentiate local, fission-, and fusion-based contributions. For completeness, we have reintroduced local contributions from insertion and substitution (these subdominant effects will be dropped again shortly). Setting the time derivative to zero, we find an integral ordinary differential equation (ODE) for the steady state distribution.

$$\partial_t\rho_{ss}(L) = 0 \tag{S33}$$

Henceforth, the subscript ss (indicating steady state) will be dropped and assumed throughout. We now approximate this condition to focus on the dominant terms driving the distribution at large lengths. Again, substitutions and insertions are subdominant in the local terms due to a length-independent target size. Additionally, $\nu L \gg 2\mu p_F$ such that the fusion outflux remains subdominant at long lengths. These approximations yield a slightly simpler steady state condition in the asymptotic $L \gg 1$ regime.

$$\partial_t\rho(L) \approx \frac{1}{2}\partial_L^2\left[(\epsilon_L+\kappa_L)L\rho(L)\right]-\partial_L\left[(\epsilon_L-\kappa_L)L\rho(L)\right] - (\nu+\iota_L)L\rho(L)+\int_L^\infty 2(\nu+\iota_\lambda)\rho(\lambda)d\lambda+\mu p_F\int_0^L\rho(\lambda)\rho(L-\lambda)d\lambda \approx 0 \tag{S34}$$

Expressing this in terms of the power-law parameterization in Equation S1, we find the following.

$$\frac{1}{2}\partial_L^2\left[(C_\epsilon L^{\tau_\epsilon}+C_\kappa L^{\tau_\kappa})L\rho(L)\right]-\partial_L\left[(C_\epsilon L^{\tau_\epsilon}-C_\kappa L^{\tau_\kappa})L\rho(L)\right]-(\nu+C_\iota L^{\tau_\epsilon})L\rho(L)+\int_L^\infty 2(\nu+C_\iota\lambda^{\tau_\epsilon})\rho(\lambda)d\lambda + \mu p_F\int_0^L\rho(\lambda)\rho(L-\lambda)d\lambda \approx 0 \tag{S35}$$

Importantly, no generic closed-form solution to this ODE can be found. First, the complications introduced by fusion are significant, as integration must be performed over the short repeat length regime, limiting our ability to decouple the long length asymptotic dynamics. Second, even when omitting fusion entirely, the remaining terms describe a second-order integral ODE, which can be recast as a third order ODE to remove explicit nonlocal transitions; unfortunately, few third order ODEs are exactly soluble.

The functional form of the fusion term fundamentally limited our ability to further disentangle the dynamics. Motivated by our computational results, we proceeded under the *ansatz* that, for long lengths $L \gg 1$, the fusion term is everywhere negligible relative to fission and local transitions, the latter providing the largest contributions to the asymptotic dynamics due to length scaling. The validity of this assumption, which we confirmed by exploring the allowed parameter space (for various parameterizations) with our computational model, is likely a consequence of both the low rate associated with $\mu p_F \ll \nu L$ and the rapid geometric decay in the short length regime followed by the further (though sub-geometric) monotonic decay at longer lengths. In contrast, Equation S15 demonstrates the importance of fusion to the short length dynamics, which exactly balance length decreases due to ν substitutions. We

found non-negligible contributions from fusion only in the substitution-dominated short length regime, consistent with our analytic results for short repeats, and in cases where the distribution is far from steady state. A more principled argument for the relative suppression of fusion at long lengths likely exists, but is unnecessary for the present purposes. For further analysis, we proceed under the following approximation of Equation S35 for $L \gg 1$, which retains nonlocal transitions only in the form of repeat fission.

$$\frac{1}{2}\partial_L^2\left[(\epsilon_L+\kappa_L)L\rho(L)\right]-\partial_L\left[(\epsilon_L-\kappa_L)L\rho(L)\right]-(\nu+\iota_L)\,L\rho(L)+\int_L^\infty 2\,(\nu+\iota_\lambda)\,\rho(\lambda)d\lambda\approx 0 \tag{S36}$$

Again using the parameterization in Equation S1, steady state is maintained under the following approximation.

$$\frac{1}{2}\partial_L^2\left[\left(C_\epsilon L^{\tau_\epsilon}+C_\kappa L^{\tau_\kappa}\right)L\rho(L)\right]-\partial_L\left[\left(C_\epsilon L^{\tau_\epsilon}-C_\kappa L^{\tau_\kappa}\right)L\rho(L)\right]-\left(\nu+C_\iota L^{\tau_\epsilon}\right)L\rho(L)+\int_L^\infty 2\left(\nu+C_\iota\lambda^{\tau_\epsilon}\right)\rho(\lambda)d\lambda\approx 0 \tag{S37}$$

The above expression describes four distinct effects that collectively lead to steady state, parameters permitting: bidirectional diffusion due to net repeat instability from the combined effects of expansion and contraction, expansion-contraction bias generating a directional flux, net (substitution- and/or insertion-based) outflux due to repeat fission, and net nonlocal (substitution- and/or insertion-based) influx due to fission of any longer repeats. Strictly speaking, the outflux due to fission is technically local in the equation (despite representing non-local transitions, appropriately accounted for by the nonlocal influx), as it corresponds to the rate of interruptions of repeats in the focal length class without reference to the subsequent repeat lengths.

4.5 Decomposition of parameter space into dynamical regimes

To better understand the dynamics, we characterized the behavior of the multiplier-coupled power-law model of instability rates in qualitatively distinct parameter regimes as primarily controlled by a sum of two, three, or four of the terms in the more complete steady state approximation shown in Equation S37. These regimes can be identified by studying the length scaling associated with each term, which indicates the primary difference between local and fission-based contributions to changes in length. In contrast to the generality of Equation S34 (and, assuming subdominant fusion; Equation S36), the following decomposition is dependent on the details of both our empirically estimated mutation rates and the parameterization in Equation S1; differing estimates or parameterizations may result in differences in the quantities of importance to the dynamics, but are unlikely to fundamentally alter the subsequent qualitative conclusions about the steady state distribution.

4.5.1 Asymptotic length dependence of local transition rates and $\Delta\tau$

First focusing on local length changes, the quantity $\Delta\tau$ (see Equation S24) provides an indicator variable for the sign of the bias term at asymptotically large lengths. This can be seen by manipulating the length dependence inside the first derivative term representing the directional (i.e., signed) per repeat rates.

$$(\kappa_L L-\epsilon_L L)=(C_\kappa L^{\tau_\kappa}-C_\epsilon L^{\tau_\epsilon})L=(C_\kappa L^{\Delta\tau}-C_\epsilon)L^{1+\tau_\epsilon} \tag{S38}$$

The sign of this term determines the asymptotic dominance of either the expansion or contraction rate (i.e., the bias at long lengths) and is dependent only on $\Delta\tau$ (i.e., the constants C_ϵ and C_κ do not scale with length). For convenience, $\Delta\tau\equiv\tau_\kappa-\tau_\epsilon$ was defined to correspond to the sign of $\kappa_L-\epsilon_L$ such that positive $\Delta\tau$ indicates asymptotic contraction bias (and stability, explained below).

$$\lim_{L\to\infty}\operatorname{sign}[\kappa_L-\epsilon_L]=\operatorname{sign}[\Delta\tau] \tag{S39}$$

As described in Equation S23, $\Delta\tau$ (provided $\Delta\tau\neq 0$) specifies a length L^* at which the sign of $\kappa_L-\epsilon_L$ can reverse if the directional flux changes above $L=9$ (relative to the initially expansion-biased rates at low lengths, $\epsilon_{L=9}>\kappa_{L=9}$). The overly simplistic power law parameterization limits the behavior to, at most, one such sign change in the long length regime (i.e., there is no more than one intersection between two monotonically growing power law functions): asymptotic contraction-bias ($\Delta\tau\geq 0$) requires one sign reversal, while asymptotic expansion bias ($\Delta\tau\leq 0$) depicts a consistent directional flux throughout the long length regime (due to the empirically estimated expansion bias at $L=8$). The asymptotic length dependence of the directional flux coefficient is determined by the scaling of the faster of the two rates (i.e., the larger exponent, τ_ϵ or τ_κ).

$$\lim_{L\to\infty}\left(\kappa_L-\epsilon_L\right)L\propto\operatorname{sign}[\Delta\tau]mL^{1+\max[\tau_\epsilon,\tau_\kappa]} \tag{S40}$$

Here, the dependence on m is common to the definitions of C_ϵ and C_κ (see discussion below). As can be seen in Equation S28, the larger of the two exponents also dictates the asymptotic length dependence of the diffusion coefficient in the second derivative term.

$$\lim_{L\to\infty}(\epsilon_L + \kappa_L)L \propto mL^{1+\max[\tau_\epsilon,\tau_\kappa]} \tag{S41}$$

Monotonicity of both instability rates requires $\tau_\epsilon, \tau_\kappa \geq 0$. As a result, the sign of $\Delta\tau$ dictates the larger exponent (i.e., when $\Delta\tau > 0$, $\max[\tau_\epsilon,\tau_\kappa] = \tau_\kappa$; when $\Delta\tau < 0$, $\max[\tau_\epsilon,\tau_\kappa] = \tau_\epsilon$) and thus the asymptotic length scaling for the coefficients of both directional and diffusive local changes in length. Because $\Delta\tau$ adequately characterizes the local dynamics (which, in turn, determine the shape of the DRL at steady state), the parameter space roughly decomposes along lines of constant $\Delta\tau$. Parameter combinations with the same $\Delta\tau$ result in similar steady state DRLs (see **Supplementary Figure 11**), which, in our inference, form a ridge of similar posterior probabilities based on their divergence from the empirical DRL (see **Figure 5a** and **Supplementary Figure 8a**).

The linear dependence on m is common to the dominant local terms in Equation S37 ($C_\epsilon, C_\kappa \propto m$ in Equation S1), which describe the collective effects of repeat instability due to expansion and contraction. Unlike $\Delta\tau$, m contains no information about the relative contributions of expansion, contraction, and insertion (again linear in m; $C_\iota \propto m$). However, because substitution-driven transitions do not depend on m, this parameter captures a competition between instability-related effects (including fission due to insertion) and fission due to substitution. This generates deviations from lines of constant $\Delta\tau$ that are visible at low m values in **Figure 5a** and **Supplementary Figure 8a**. At low m and slower growth rates ($\tau_\epsilon, \tau_\kappa \lesssim 1$), substitutions play a more substantial role (further discussed below).

4.5.2 Relative strength of substitution- and insertion-driven fission and L_{fis}

In contrast to the local dynamics, repeat fission is asymptotically dominated by insertion, which outcompetes the substitution rate at asymptotically long lengths. A characteristic transition length L_{fis} emerges from the multiplier-coupled model, above which the fission rate is dominated by insertion-based interruptions. The fission-transition length can be found by comparing the per-target rates of substitution ν and insertion $C_\iota L^{\tau_\epsilon}$.

$$L_{\text{fis}} \equiv \left(\frac{\nu}{C_\iota}\right)^{1/\tau_\epsilon} = 9\left(\frac{\nu}{m \times \iota_8}\right)^{1/\tau_\epsilon} \tag{S42}$$

Here, definition of C_ι was used to show the explicit dependence on m. From our empirical estimates, $\nu/\iota_8 \approx 40$ for mononucleotide A-repeats (see **Figure 3b**); thus, for values of $m \lesssim 40$ (which includes all computationally explored parameters), the ratio being exponentiated is greater than one. Consequently, as τ_ϵ increases, L_{fis} decreases, eventually exiting the long length regime (i.e., $L_{\text{fis}} \sim 9$ for $\tau_\epsilon \gg 1$) such that, for large τ_ϵ, all fission events in long repeats are insertion-dominated. Substitutions dominate more of the long length regime when both τ_ϵ and m are small (i.e., for $\tau_\epsilon < 1$ and $m \ll 50$), while substitutions are subdominant at more long lengths when τ_ϵ and m are large.

The quantities τ_ϵ and m together characterize the asymptotic length dependence of repeat fission by determining the value of L_{fis}, the length at which a switch occurs from substitution- to insertion-dominated fission (at lengths $L < L_{\text{fis}}$ and $L > L_{\text{fis}}$, respectively). In the parameter regime where fission is entirely insertion-dominated at long length (i.e., $L_{\text{fis}} \sim 10$ when m, τ_ϵ, or both parameters are sufficiently large), all relevant terms in Equation S37 are linear in m such that the dynamics are independent of this parameter; in this case, the relative importance of local transitions and fission can be characterized by the values of τ_ϵ and $\Delta\tau$ (or, equivalently, τ_ϵ and τ_κ). In this extreme, in addition to the decoupling between short and long repeat dynamics, long repeats are predominantly subject to insertion- and deletion-based mutations (i.e., repeat instability-generated expansions, contractions, and insertions) and evolve *mechanistically* independently (i.e., change length predominantly due to distinct mutational processes) from short repeats; importantly, this claim implicitly requires that long repeat dynamics are independent of μ (i.e., fusion remains negligible). In the opposite extreme, where insertions remain irrelevant at all populated lengths, the dynamics are independent of τ_ϵ and can be represented in the space of $(\Delta\tau, m)$.

In Section 6 this supplemental note, we assess the range of plausible values of L_{fis} suggested by the results of our Bayesian inference procedure.

4.5.3 Distinguishable dynamical regimes

The sign of $\Delta\tau$ decomposes the space into two primary regions and the boundary between them, only one of which evolves towards a recognizable steady-state equilibrium on reasonable timescales. The repeat length distribution only stabilizes for the subset of the parameter space with $\Delta\tau \geq 0$, which we refer to as the (asymptotically) *contraction-biased* regime. Expansion-biased directional flux ($\Delta\tau < 0$) leads to indefinite repeat expansion, exceeding the net

shortening effects of contraction and repeat fission (the boundary at $\Delta\tau = 0$ is discussed below). In contrast, asymptotic contraction bias, which is directionally consistent with fission, truncates the distribution at finite lengths, leading to a stable distribution (consistent with our empirical observations). The collection of stable parameter combinations can be further decomposed based on the extent to which contraction dominates over expansion (i.e., the value of $\Delta\tau$); this is the primary subject of our subsequent analysis. Within each subregime of $\Delta\tau > 0$, the full set of changes in repeat length in Equation S32 that lead to a stable distribution can be reduced to a subset of effects that approximate the dominant contributions that shape the distribution. Parameter combinations with $\Delta\tau \gg 1$ are controlled largely by a local balance between diffusion and directional flux (contraction-biased at most or all lengths in the $L \gg 1$ regime), both of which dominate over fission. Intermediate values $1 \gtrsim \Delta\tau > 0.6$ show a balance between local transitions and an outflux due to fission (i.e., the non-integral terms representing fission in Equation S37). Weak contraction bias (very roughly $\Delta\tau \lesssim 0.5$) requires a full accounting of local effects and both the influx and outflux due to fission (i.e., the full set of terms in Equation S37).

At all points in the stable regime, the net increase in repeat length due to expansion (ignoring subdominant μ contributions) is counteracted by the combined effects of contraction (which exceeds expansion above some length L^*) and fission due to insertions and/or substitutions. Based on our computational analyses, fusion remains far too infrequent to stabilize long repeats (justifying the approximation in Equation S37). This regime is bounded by $\Delta\tau = 0$, but we note that very low positive values of $\Delta\tau$ have expansion-dominated rates across an extended range of lengths (i.e., $L^* \to \infty$ as $\Delta\tau \to 0$; see Equation S23), truncating the distribution at unrealistically high L. These parameters (i.e., small, positive $\Delta\tau$) are inconsistent with the empirical distribution.

The remaining set of parameter combinations fall into two categories, both of which are implausible with respect to the collection of empirical observations presented in this manuscript: those with very low instability rates (i.e., very slowly evolving distributions that do not equilibrate on evolutionarily-relevant timescales) and those with unstable dynamics (i.e., rapid aggregation of repeats and explosive growth in genome size). Exceedingly slow evolution occurs if m is order one and both exponents approach zero (i.e., $\tau_\epsilon, \tau_\kappa < 1$ such that τ_ϵ and $\|\Delta\tau\|$ both remain small). These parameter combinations depict repeat instability rates that grow much more slowly with length than our empirical rate estimates (see **Figure 3b**); as a result, this regime is entirely ruled out under the informative prior (indicating inconsistency with rate estimates based on popSTR [1]; see **Figure 5a**). We refer to this region of parameter space as the *slowly evolving* regime, as the repeat instability rates depicted remain scantly above the estimated substitution rates, even for the longest well-populated lengths in the human genome.

Parameter combinations that are dynamically unstable (referred to as the *unstable* regime) correspond to universally expansion-biased dynamics ($\Delta\tau < 0$, excluding slowly evolving parameters that may or may not equilibrate). This regime comprises the majority of the parameter space explored in our computational model, which can be dynamically disallowed simply by requiring steady state based on our empirical findings. These parameters represent a non-equilibrium dynamical regime subject to strong nonlinear effects that result in increasingly rapid changes in the repeat length distribution. In the multiplier-coupled model, parameters with $\Delta\tau = 0$ are similarly unstable due to the universal expansion-bias at all lengths, which is inherited from the empirically observed bias at $L = 8$ (i.e., $\kappa_9 - \epsilon_9 = m(\kappa_8 - \epsilon_8) > 0$) and subsequent parallel growth of expansion and contraction.

Last, we note that the decomposition of the dynamics is dramatically simplified for mid-to-large multipliers $m \gtrsim 4$. We focus our discussion of the analytics on this case, where the dynamics can be characterized largely by $\Delta\tau$ alone (see, e.g., **Figure 5a**). However, the same dynamics apply to smaller m, values with the additional complication that the balance between substitutions and insertions breaks the dynamical similarity along lines of constant $\Delta\tau$ (this can be seen as curvature along posterior ridges in **Figure 5a** due to more explicit dependence on τ_ϵ near the origin).

4.6 Unstable dynamics in the asymptotically expansion-biased regime $\Delta\tau \leq 0$

We briefly discuss the dynamics of the unstable regime, as it informs our considerations for more realistic parameter combinations. There are two distinguishable cases where steady state cannot be assumed, contrary to the observation of long term maintenance of the DRL across the primate phylogeny. First, the expansion rate, which is initially dominant at $L = 9$ (i.e., $\epsilon_9 > \kappa_9$), may have a length dependence that rapidly outcompetes that of contraction, resulting in increasingly larger expansion-bias with increasing length. This corresponds to $\Delta\tau < 0$ with large magnitudes $\|\Delta\tau\| \gg 1$. In the second case, which occurs for relatively small values of $\|\Delta\tau\|$ approaching $\Delta\tau = 0$ (i.e., when $\tau_\epsilon = \tau_\kappa$) the length dependence of expansion increases with length comparably to, or slightly in excess of, the length-dependence of the contraction rate. In this case, the directional flux is minimized such that the relative importance of repeat fission becomes inflated. In the former case with highly dominant expansion, the bias generates a large directional flux that rapidly increases repeat lengths. In this regime, the flux is well approximated by the rate $C_\epsilon L^{1+\tau_\epsilon}$ (i.e., τ_κ is negligible at all lengths $L > 10$) and the rate of changes in length rapidly accelerates with increasing repeat

length. This nonlinearity results in an indefinitely extending tail, some of which feeds back into lower length classes due to fission, as large τ_ϵ also generates large rates of insertion-based repeat fission $C_\iota L^{1+\tau_\epsilon}$; however, the coefficient C_ι is roughly two orders of magnitude smaller than C_ϵ such that fission alone is unable to counteract expansion alone at any length. Repeat fission additionally increases the mass of the distribution, as it does not conserve repeat number (one repeat is replaced with two shorter length repeats). These shorter repeats are then subject to the large directional push due to expansion, which further increases the weight in the distribution tail. This feedback loop generates a rapid, indefinitely growing genome, which reshapes the distribution. Rapid fission of any given repeat in the long tail equiprobably adds mass to the distribution in all shorter length classes, an integrated effect that is increased with increasing mass above a given length. This eventually leads to an extreme influx into the shortest length classes, inherently coupling the dynamics in the short and long length regimes (i.e., violating our assumption of separability and distorting the substitution-based geometric distribution). In addition to the unstable dynamics, the indefinite extension of the distribution tail quickly results in highly relevant expansion probabilities approaching one (because power-law rates do not saturate), which simultaneously makes computational modeling impractical across this regime and will invariably result in characteristic changes to the shape of the distribution when the power law parameterization is corrected to saturate at this probabilistic bound.

In the second case, for $\Delta\tau$ near zero (still assuming $\tau_\epsilon, \tau_\kappa > 1$ to avoid the slowly evolving regime), the directional flux is again expansion dominated and non-vanishing. Here, the relative importance of fission is inflated, which limits the rate at which the tail extends. However, the difference between the expansion and contraction rates, even at $L = 8$, is substantially in excess of the insertion rate. This leads to the inability of repeat fission to independently counteract the directional flux at all lengths.

$$(C_\epsilon - C_\kappa)L^{1+\tau_\epsilon} \gg C_\iota L^{1+\tau_\epsilon} \tag{S43}$$

In this sense, contraction must be sufficiently large to mitigate expansion in order for the distribution to truncate at finite length. This only occurs when $\Delta\tau > 0$ such that the contraction rate approaches, and eventually exceeds, the expansion rate (though this may occur at an extremely large length for the smallest values of $\Delta\tau > 0$). This defines a bound for the contraction-biased regime that leads to steady state. A small, non-negligible contribution from fission is relevant above this bound, but is insufficient to control the directional push from expansion for very low $\|\Delta\tau\|$. We note that this effort may be aided by substitution-based fission, but the associated length scaling (i.e., the fission rate νL) only becomes relevant for small multipliers m when $\tau_\epsilon \lesssim 1$ (i.e., when $\nu/C_\iota \sim \mathcal{O}(1)$ and $\nu/C_\iota L^{\tau_\epsilon} \sim \mathcal{O}(1)$ across relevant lengths); parameters in this range correspond to sufficiently small rates that evolution proceeds exceedingly slowly, as discussed above. Relative to the directional flux, diffusive changes in length are also magnified when $|\Delta\tau|$ is small, but do not stabilize the distribution.

4.7 Stable dynamics in the asymptotically contraction-biased regime $\Delta\tau > 0$

Given the similarity between primate DRLs (**Figure 1b**), realistic parameter combinations in the multiplier-coupled model sit within the subset that evolve towards stable, steady-state distributions. In contrast to the unstable regime, parameter combinations with asymptotic contraction bias $\Delta\tau > 0$ result in a distribution that approaches a stable steady state. Perhaps more intuitively, this regime can be equivalently characterized by L^*, the length at which expansion and contraction rates are equal and the directional flux vanishes. For all values $\Delta\tau > 0$, evaluation of Equation S23 shows that $L^* \geq 9$ (and $L^* \geq 10$ for realistic values of $\Delta\tau$) such that this intersection sits within, or near the boundary of, the long length regime. As the combination of our empirical estimates at $L = 8$ and the multiplier-coupled model (namely, $m > 0$) dictate that the expansion rate exceeds the contraction rate at length $L = 9$, an intersection between the rates must occur prior to the asymptotic dominance of contraction. Repeat lengths below L^* are necessarily expansion-biased and those above L^* are contraction-biased, with no directional flux at L^*. For large values of $\Delta\tau$, the value of L^* approaches a length close to $L = 10$. For very small $\Delta\tau$, this can sit at very large lengths, approaching $L^* \to \infty$ as $\Delta\tau \to 0$. At many values of $\Delta\tau > 0$, L^* occurs well within the tail of well-populated lengths in the whole-genome human DRL.

The transition between expansion- and contraction-biased lengths controls and complicates the dynamics. The rates of expansion and contraction can remain within the same order of magnitude over an extended range when $\Delta\tau$ is small, as the approach to L^* is slow from either side. This can result in a first derivative term (i.e., the directional flux) that remains relatively small over lengths spanning part or all of the distribution tail. In this case, the diffusion term, which arises as a subdominant correction to the discrete local behavior (see Section 4.1.2) plays an important role in the extended neighborhood of L^*. For the smallest values of $\Delta\tau \ll 1$, the intersection at L^* occurs at extreme lengths above those populated in the empirical distribution. This results in a dramatically extended tail due to a wide range of expansion-biased lengths before contraction bias becomes appreciable. Additionally, this generates a dramatically larger genome (with an excess of very long repeats) inconsistent with the observed range of mammalian

genome sizes. This is somewhat similar to the unstable dynamics described above, but is eventually counteracted by sufficiently large contraction rates that truncate the distribution and stabilize the (presumably unrealistic) shape of the distribution.

As mentioned above, the dynamics of long repeats across the space of parameters with $\Delta\tau > 0$ can be approximated by Equation S37 under the assumption that repeat fusion is sufficiently infrequent. However, the dynamics reduce further in parts of this regime where fission (influx and, to a lesser extent, outflux) can be treated as negligible. While only appropriate for some parameter combinations, such approximations can be useful because they eliminate the need to explicitly treat the nonlocal effects of fission influx, which reduces the second-order integro-differential equation to a second-order ODE.

4.7.1 Strong asymptotic contraction bias

We first attempted to describe the dynamics in the regime with sufficiently large $\Delta\tau$ such that the majority of long repeats have contraction-biased rates (i.e., $L^* \sim 10$ occurs immediately above the short repeat regime). In this case, the diffusion term remains relevant because L^* lies in the long length regime but the directional flux becomes increasingly relevant with increasing length, an effect magnified by necessarily larger $\Delta\tau$. For most contraction-biased parameter combinations, the dynamics of Equation S37 are well approximated by the following.

$$\frac{1}{2}\partial_L^2\left[(\epsilon_L + \kappa_L)L\rho(L)\right] - \partial_L\left[(\epsilon_L - \kappa_L)L\rho(L)\right] - (\nu + \iota_L)\,L\rho(L) \approx 0 \tag{S44}$$

In terms of the parameterization in Equation S1, this becomes the following.

$$\frac{1}{2}\partial_L^2\left[(C_\epsilon L^{\tau_\epsilon} + C_\kappa L^{\tau_\kappa})\,L\rho(L)\right] - \partial_L\left[(C_\epsilon L^{\tau_\epsilon} - C_\kappa L^{\tau_\kappa})\,L\rho(L)\right] - (\nu + C_\iota L^{\tau_\epsilon})\,L\rho(L) \approx 0 \tag{S45}$$

Here, we have treated the integral terms describing the influx due to fission as subdominant. These contributions are outcompeted by each of the remaining rates, which scale asymptotically in length with larger exponents (i.e., as $L^{1+\tau_\kappa}$ or $L^{1+\tau_\epsilon}$).

4.7.2 Strictly local approximation for strong asymptotic contraction bias $\Delta\tau \gg 1$

In the regime of largest $\Delta\tau$, the outflux due to fission is negligible, as well; the asymptotic scalings of the directional flux and diffusion coefficients ($\lim_{L\gg1}(\kappa_L L \pm \epsilon_L L) \approx C_\kappa L^{1+\tau_\kappa}$) outcompetes the fission outflux at very large lengths ($L^{\tau_\kappa} \gg L^{\tau_\epsilon}$). This defines a dynamical sub-regime within the space of contraction-biased parameter values, with dynamics well approximated by the following balance.

$$\frac{1}{2}\partial_L^2\left[(C_\epsilon L^{\tau_\epsilon} + C_\kappa L^{\tau_\kappa})\,L\rho(L)\right] - \partial_L\left[(C_\epsilon L^{\tau_\epsilon} - C_\kappa L^{\tau_\kappa})\,L\rho(L)\right] \approx 0 \tag{S46}$$

In this regime, sufficiently rapid decay in $\rho(L)$ results in a net negative directional flux (the correct sign associated with contraction bias) that counteracts the strictly positive diffusion term. For example, when $m = 4$, this expression provides a good approximation to the dynamics primarily for $\Delta\tau > 1.5$ (see **Supplementary Figure 14**) and breaks down as fission becomes increasingly relevant for weaker contraction bias (for larger multipliers, this regime breaks down at lower $\Delta\tau$; e.g., for $m = 16$, Equation S46 remains a good approximation for $\Delta\tau > 1$; see **Supplementary Figure 16**). In this regime, L^* occurs at or adjacent to $L = 10$ such that nearly all long lengths are contraction-biased. For example, the parameter combination $(m, \tau_\epsilon, \tau_\kappa) = (4, 0, 2)$ results in $L^* \approx 11$ and a contraction rate of roughly 2.5 times the expansion rate at $L = 18$, while for $(m, \tau_\epsilon, \tau_\kappa) = (4, 0, 4)$, $L^* \approx 12$ and the contraction rate is roughly tenfold the expansion rate at $L = 18$. In the more extreme case of $\Delta\tau = 4$, we can better understand the truncation of the distribution by further approximating Equation S46 under the very rough assumption that the asymptotic dependence is immediately relevant for lengths $L > L^*$.

$$\frac{1}{2}\partial_L^2\left[L^{1+\tau_\kappa}\rho(L)\right] \approx -\partial_L\left[L^{1+\tau_\kappa}\rho(L)\right] \tag{S47}$$

Noting that the contraction rate constant C_κ cancels such that this equation is dependent only on the exponent τ_κ, an approximation for the asymptotic shape of the steady state distribution can be obtained in closed form (valid only for lengths $L \gg L^*$).

$$\lim_{\Delta\tau\gg1}\rho(L) \approx c_1 L^{-(1+\tau_\kappa)} + c_2 L^{-(1+\tau_\kappa)} e^{-2L} \tag{S48}$$

To evaluate the accuracy of this rough approximation, the arbitrary constants c_1 and c_2 can be found using values from our computational model at two lengths L_1 and L_2 (where $L_1, L_2 \gg L^*$) to constrain the distribution at $\rho(L_1)$ and $\rho(L_2)$. Comparing this expression to numerical solutions, we found reasonable agreement for the most extreme values of $\Delta\tau > 0$ (e.g., $\Delta\tau$ = 3–4 for $m > 4$, the largest $\Delta\tau$ with computational modeled DRLs) at the longest lengths $L > L^*$ in the distribution and an expected departure as $L \to L^*$.

4.8 Intermediate asymptotic contraction bias

Intermediate values of $\Delta\tau > 0$ (e.g., roughly $1.5 > \Delta\tau \gtrsim 0.7$ for $m = 4$; see **Supplementary Figure 14**) require a description of the outflux due to fission that appears in Equation S45. At the larger end of this range (e.g., $\Delta\tau \sim 2$), the numerical solution to Equation S46, which omits all effects of fission, approximates the asymptotic shape of the distribution for lengths $L > L^*$. This indicates that the impact of outflux due to fission is primarily localized to intermediate lengths $L^* > L > 10$ and therefore most relevant when the rate of expansion exceeds contraction. At the same time, this suggests that the truncation of the distribution is driven by the contraction-biased directional flux, rather than fission alone or due to the combined effects of fission and contraction.

4.9 Weak asymptotic contraction bias

For smaller values of $\Delta\tau$ that approach $\tau_\epsilon = \tau_\kappa$ (e.g., roughly $\Delta\tau < 0.7$ for $m = 4$; see **Supplementary Figure 14**), the dynamics revert to Equation S37, which omits only fusion. The resulting distributions stabilize, in part, due to the nonlocal influx from fission of longer repeats. For these parameter combinations, L^* sits in the middle of the long length tail; for lengths $L > L^*$, the distribution is well-approximated by solutions to Equation S45. This is consistent with a nonlocal net flow from lengths $L > L^*$ to lengths $L^* > L > 10$ and indicates that the dynamically relevant effects of fission influx are primarily localized to the latter. The net effects of fission alone (i.e., fission influx minus outflux) result in a net loss of long repeats, with little gain from any existing longer length repeats, and a compensatory net gain of intermediate length repeats within the distribution tail (exceeding those lost to the short length regime). With decreasing values of $\Delta\tau \to 0$, L^* approaches very large values, extending the range of lengths that receive a net influx. All effects represented in S37 are thus required to adequately approximate the dynamics that lead to a steady state distribution when $\Delta\tau < 1$ (and a potentially broader range, depending on m).

The nonlocal integral dependence in Equation S37 that describes the influx due to fission complicates the steady state condition in this regime. To find solutions, we re-expressed the second-order integro-differential equation as a third order differential equation by applying an overall length derivative to each term.

$$\frac{1}{2}\partial_L^3\left[\left(C_\epsilon L^{\tau_\epsilon} + C_\kappa L^{\tau_\kappa}\right)L\rho(L)\right] - \partial_L^2\left[\left(C_\epsilon L^{\tau_\epsilon} - C_\kappa L^{\tau_\kappa}\right)L\rho(L)\right] - \partial_L\left(\nu + C_\iota L^{\tau_\epsilon}\right)L\rho(L) + \partial_L\left[\int_L^\infty 2\left(\nu + C_\iota \lambda^{\tau_\epsilon}\right)\rho(\lambda)d\lambda\right] \approx 0 \qquad \text{(S49)}$$

Taking this derivative allows us to apply the fundamental rule of calculus to replace the derivative the integral with the integrand evaluated at the integration limits. Under the assumption that the distribution decays sufficiently rapidly such that $L^{\tau_\epsilon}\rho(L) \to 0$ as $L \to \infty$, the integral term becomes the following.

$$\partial_L\left[\int_L^\infty 2\left(\nu + C_\iota \lambda^{\tau_\epsilon}\right)\rho(\lambda)d\lambda\right] = -2\left(\nu + C_\iota L^{\tau_\epsilon}\right)\rho(L) \qquad \text{(S50)}$$

This allows us to re-expresses the second-order integro-differential equation as the following third order ODE.

$$\frac{1}{2}\partial_L^3\left[\left(C_\epsilon L^{\tau_\epsilon} + C_\kappa L^{\tau_\kappa}\right)L\rho(L)\right] - \partial_L^2\left[C_\epsilon L^{\tau_\epsilon} - \left(C_\kappa L^{\tau_\kappa}\right)L\rho(L)\right] - \partial_L\left(\nu + C_\iota L^{\tau_\epsilon}\right)L\rho(L) - 2\left(\nu + C_\iota L^{\tau_\epsilon}\right)\rho(L) \approx 0 \qquad \text{(S51)}$$

After applying a length derivative to the steady state condition $\partial_L(\partial_t\rho(L)) = 0$, this now corresponds to a constraint on $\partial_L\rho(L)$, the flux through each length L. This can be seen by swapping the order of the length and time derivatives (i.e., $\partial_t(\partial_L\rho(L)) = 0$), dictating that the various fluxes through each length must sum to a time-independent constant ϕ_L. In the special case where $\phi_L = 0$, this corresponds to a steady state condition that maintains the shape of the distribution in equilibrium. We confirmed via our computational model that, once steady state was reached, the net flux through each individual bin independently vanished (see **Supplementary Figures 18–20**), indicating that the equilibrated state is maintained in a detailed balance. Insofar as our approximations remain valid, Equation S51 provides a local expression for the steady-state flux through the repeat length distribution in the large length regime; this includes the nonlocal contributions of repeat fission to the flux, represented by boundary effects at length L

(more accurately, at length $L+2 \approx L$). While this equation cannot be solved analytically, numerical solutions can be readily obtained.

Assuming the effects of fusion remain subdominant in the long length regime, Equation S51 captures the full set of relevant dynamics associated with repeat length transitions. Solutions to this equation, obtained after applying the additional constraint that the fluxes vanish, are applicable across the full range of parameters that evolve towards steady state distributions $\Delta\tau > 0$. In contrast, Equations S45 and S46 are approximations to these dynamics appropriate in a subset of parameter space (very roughly, when $2.5 > \Delta\tau > 1$ and when $\Delta\tau < 1$, respectively). However, in addition to aiding in our intuitive understanding of the dynamics, the absence of the third derivative in the latter equations makes numerical solutions more reliable, as they are less susceptible to instabilities in numerical techniques; this facilitates a slightly more reliable comparison between the numerical solutions and results of our computational model.

4.10 Obtaining numerical solutions to the steady state dynamics for $\Delta\tau < 0$

To compare our analytic understanding of the dynamics to the results of our computational model for generic parameters, we resorted to solving Equations S45, S46, and S51 numerically. All numerical solutions were obtained using the NDSolve function in Mathematica 14.0 [2]. Solutions to second-order differential equations require the specification of two additional constraints that together fix the normalization constant and the linear coefficient that determines the relative weight of the two real solutions to the equation, if both exist. The third order equation for constant flux requires a third constraint that ensures vanishing flux.

For the second-order equations, we chose to constrain the values of $\rho(L)$ at two lengths, L_1 and L_2, using the results of our computational model (i.e., $\rho(L_1) = \rho_{sim}(L_1)$ and $\rho(L_2) = \rho_{sim}(L_2)$, where $\rho_{sim}(L)$ is the value of the computationally propagated (i.e., 'simulated') distribution at length L once it has reached steady state). The choice of L_1 and L_2 is somewhat arbitrary, provided they are both in the long length regime where the continuum approximation is valid and that any additional constraints on the regime of validity are respected (e.g., $\Delta\tau > 0$ and sufficiently far from $\Delta\tau = 0$, $\tau_\epsilon, \tau_\kappa \geq 0$, etc.). For the majority of comparisons, we chose two lengths that are well defined for any parameter combination: $L_1 = L^*$ (rounded to the nearest integer value) and $L_2 = L_{\text{trunc}}$, where L_{trunc} is the length bin for which the occupancy of the non-normalized distribution first drops below a single count (i.e., $L_{\text{trunc}} = \min[L \text{ for } \rho(L) < 1]$) and represents the truncation point of the distribution. L_{trunc} is uniquely defined because of two properties: *1.* all computationally modeled distributions decayed monotonically, and *2.* we propagated the expected DRL under a mean field approximation, omitting stochastic effects. All comparisons were made using non-normalized DRLs to identify the truncation point of the distribution and obtain L_{trunc}.

For solutions of Equation S51, we chose to apply the third constraint by fixing the value of the DRL at $L_3 = L_2 - 1$ for convenience. We note that inappropriate choice of L_3 outside of the regime of validity of the approximation can result in numerical instability in solutions to the third order ODE (e.g., constraining the solution using a length class $\rho_{sim}(L_3)$ that has not yet equilibrated). We chose not to use the intuitive lower bound of the long length regime at $L = 10$ to identify any potential effects associated with the breakdown of the continuum approximation.

For any parameter combinations with $L^* < 10$ (i.e., non-equilibrium cases with $\Delta\tau < 0$) we chose a lower bound at $L_1 = 10$ to avoid values in the short length regime; however, unstable dynamics were compared to numerical solutions only to confirm significant departure from any steady state characterized by Equation S51 and to identify any common features that emerged.

Finally, for all comparisons in the long length regime, the value of ν was replaced with $\nu_{fission}$, the appropriate rate estimated from the three-unit context $AAA \to ABA$ in which substitutions $A \to B$ result in repeat fission. Mutation rates μ and ν that appear in Equation S10 were replaced with distinctly estimated rates relevant for the three-unit contexts associated with local transitions due to substitutions defined by μ_{local} (i.e., the summed rates of $ABB \to AAB$ and $BBA \to BAA$ substitutions) and ν_{local} (the summed rates of $AAB \to ABB$ and $BAA \to BBA$ substitutions), respectively.

5 Comparison between numerical solutions and computationally modeled distributions

As described above, numerical solutions to Equations S45, S46, and S51 were compared across the space of parameter combinations that led to steady state distributions. Because our computational model obtained results over a large, but finite number of iterations, parameters corresponding to insufficiently high mutation rates failed to equilibrate in the allotted time (i.e, in a number of iterations corresponding to at least 10^9 generations of evolution, after accounting for a factor that progressively rescales time to increase computational speed). These slowly evolving computational

results were localized to the lowest values of $(\tau_\epsilon, \tau_\kappa)$ for the lowest multipliers $m \leq 2.5$ (i.e., points closest to the origin of the $(\tau_\epsilon, \tau_\kappa)$ plane for $m \leq 2.5$; for larger values of $m \geq 4$, all parameters equilibrated sufficiently quickly) and spanned a larger range of parameter values for smaller m. For $m = 2.5$, this roughly corresponds to parameter values $\tau_\epsilon, \tau_\kappa \lesssim 1$. In this parameter range, the ridge of roughly equivalent posterior values (calculated by comparing computationally modeled distributions at the final time point to the empirical distribution) deviates from lines of constant $\Delta\tau$. This corresponds to the point at which substitution becomes non-negligible and substitution-based fission occurs at a rate comparable to or greater than insertion-based fission. Given indefinite time to evolve, such parameter combinations likely equilibrate, provided the combined action of contraction, substitution, and insertion is sufficient to truncate the distribution at finite length. As no equilibrium was reached, these points were excluded from our comparison to numerically produced steady-state distributions. Additionally, all such parameters were excluded from the 95% HDR of the posterior when using an informative prior (see **Figure 5a**).

For all points outside of this slowly evolving region, equilibrated steady state distributions are quantitatively similar along lines of constant $\Delta\tau$. The following plots show comparisons at points along an anti-diagonal line perpendicular to $\Delta\tau = 0$ defined by $\tau_\epsilon + \tau_\kappa = 3.5$; for comparison, we included parameter combinations with exponents $\tau_\epsilon = \tau_\kappa = 1.7$ (our discretization scheme did not allow for equal exponents that sum to 3.5). Parameter combinations on this line are representative of the full set of computationally modeled $\Delta\tau$ outside of the slowly evolving region and could be obtained for all considered multipliers. Along the line of $\tau_\epsilon + \tau_\kappa = 3.5$ (after adding a point with $\Delta\tau = 0$ and $\tau_\epsilon + \tau_\kappa = 3.4$), we selected examples that span the qualitatively distinct behaviors across the space of $\Delta\tau > 0$ for values $\Delta\tau = \{0, 0.1, 0.3, 0.5, 0.7, 1.1, 1.5, 2.5, 3.5\}$. The first two points were included for completeness, as they show examples of computationally modeled distributions that are not expected to be well described by numerical solutions. For $\Delta\tau = 0.1$ the location of $L^* \approx 942$, which is substantially longer than the maximum length included in our computational model at $L_{\text{bound}} = 200$; a reflective boundary condition was imposed at $L = L_{\text{bound}}$ to simultaneously prevent excessively mutation rates that preclude further computational iteration and to identify parameter combinations that result in excessively large genome size when truncation occurs at unrealistically large lengths $L_{\text{trunc}} \gg 200$ (e.g., for $\Delta\tau = 0.1$). The point at $\Delta\tau = 0$ was included as an example of unstable dynamics; the computationally modeled distribution equilibrates to the boundary condition at L_{bound} (note the balanced fluxes in the largest lengths for computational results with $\Delta\tau = 0$ in **Supplementary Figures 18i** and **19i**), resulting in an artifactual shape (i.e., the non-monotonicity in computationally modeled curves shown in **Supplementary Figures 14i**, **15i**, and **16i**).

5.0.1 Comparisons of dynamical approximations and steady-state distributions

Supplementary Figure 14 compares computationally modeled distributions for these example parameter combinations (for $m = 4$) to the geometric distribution that describes the short length regime (Equation S10 using values the average single-unit context rates $\mu = \mu_{B\to A}$ and $\nu = \mu_{A\to B}$) and to the three nested approximations of the long length tail of the distribution obtained by numerically solving Equation S45, S46, and S51. These plots contrast numerical solutions produced under the full set of dynamics (excluding fusion), in the absence of influx due to fission, and in the absence of fission entirely. By observing the lengths at which each successive approximation breaks down, we localized the incoming and outgoing flux due to fission in length space.

For the same parameter combinations, **Supplementary Figure 18** shows the total influx and outflux for each length associated with the individual effects of expansion, contraction, substitution-based fission, and insertion-based fission (as well as fusion and and local transitions due to substitutions). The total flux was separately normalized for each bin (for visualization purposes, as the true magnitudes differ dramatically); bins with equal incoming and outgoing net flux have reached equilibrium (i.e., are maintained in a detailed balance). Slight deviation from this equilibrium occurred in every run, indicating a true steady state distribution was not yet reached (as expected in finite time). However, for all results of interest, the magnitude of deviation is very small. Some parameters with modest values of $\Delta\tau$ showed slight deviation from equilibrium at L=1 characteristic of disequilibrium between the A and B distributions (i.e., a source of new A counts at the $L = 1$ boundary) but showed detailed balance across all remaining classes. This is in contrast to unstable parameter combinations, which harbor large deviations from equilibrium in many or most length classes; at the same time, non-equilibrium simulations rapidly populated the largest length classes, subsequently equilibrating to the artificially imposed boundary condition at the large L boundary of the computationally modeled grid.

Supplementary Figure 19 provides an alternative characterization of the flux for each bin: the incoming and outgoing fluxes for each mutational effect were plotted separately, rather than computing their net (substitutions were separated into local and nonlocal contributions to demonstrate subdominance of the former relative to expansion and contraction). When separating into directional fluxes, the dominance of expansion and contraction over all other fluxes in both directions is made clear; the much higher rates of repeat instability dominate local fluxes throughout

the long length regime. This also leads to large-scale diffusion, which captures the significant bidirectional flux that occurs for both expansions and contractions.

Supplementary Figure 20 shows a direct comparison between the computationally modeled sum of all fluxes (including fusion) at each length for comparison to three approximations of the full dynamics: local dynamics alone, local dynamics and fission outflux (treating influx as negligible), and local dynamics along with a full model of fission (all transitions other than fusion). The accuracy of each approximation can be seen at each length bin via the overlap with the net flux under the full model. In contrast to **Supplementary Figure 14**, which compares numerical solutions that approximate the steady-state distributions, these plots directly compare components of the finite difference equation (and, in a continuum approximation, the differential equations in steady state) for each model specified by Equations S37, S45, and S46. In particular, the nonlocal interactions in Equation S37 are accounted for directly, without requiring the intermediate step that leads to Equation S51. This provides a complementary set of comparisons that lead to the same qualitative observations about the regime of validity and accuracy of each approximation across the parameter space, while retaining length-dependent information about the role of each effect across the long repeat regime. Additionally, this provides further justification for the assumption that repeat fusion remains negligible for long repeat dynamics, despite it's qualitative importance to short repeat dynamics.

Supplementary Figures 15 and **16** show comparisons between numerical and computational results for the same values of τ_ϵ, τ_κ, and $\Delta\tau$ shown in **Supplementary Figure 14**, but with multipliers of $m = 1$ and $m = 16$, respectively. For intuition about the effect of the dynamics on the genome size, all comparisons below are shown for non-normalized distributions. The total genome-wide target for A bases, $\bar{L}_A$, corresponds to the weighted mean of the non-normalized distribution: $\bar{L}_\mathrm{A} = \sum_{L=1}^{\infty} L\ P(L) \approx \int_0^\infty dL\ L\ \rho(L)$. The total genome size is the sum of $\bar{L}_A$ and the corresponding target for B bases $\bar{L}_B$. Comparing the same values of $\Delta\tau$ across multipliers m, we found qualitative consistency of the decomposition into three dynamical regimes but with boundaries that quantitatively depend on m. When m is low, the approximations represented by numerical solutions to Equation S45 (along with the rough analytic solution) and Equation S46 break down at larger values of $\Delta\tau$ (relative to the same approximations at larger m) because substitutions become more relevant, increasing the relative importance of fissions. For example, for $m = 1$, the purely local approximation in Equation S45 breaks down at or above $\Delta\tau \sim 2$ (see **Supplementary Figure 15**), rather than $\Delta\tau \sim 1$ for $m = 4$ (see **Supplementary Figure 14**) or $\Delta\tau \sim 0.5$ for $m = 16$ (see **Supplementary Figure 16**). Here, the relative rate of substitution is closer to the insertion rate and the total rate of fission is closer to the expansion and contraction rates. The relative increase in the strength of fission implies that fission plays a more substantial role in the maintenance of the steady state distribution. In contrast to the more aggressive approximations, numerical solutions to Equation S51 (i.e., the continuum model with all effects except fusion) remain accurate across the $\tau_\epsilon + \tau_\kappa = 3$ line, even at low values of m.

Supplementary Figure 17 shows comparisons between the computationally modeled distribution, numerical solutions, and the closed form approximation in Equation S48 for parameter combination within and outside of the regime of validity of the latter (i.e., only appropriate when $\Delta\tau \gg 1$). Parameter combinations are shown for $\Delta\tau = \{2, 3, 4\}$ and $m = \{1, 4, 16\}$. This rough approximation captures the asymptotic falloff of the distribution only when $\Delta\tau$ and m are sufficiently large (very roughly $\Delta\tau \gtrsim$ 3–4 when $m > 4$), failing to characterize the shape at lengths closest to the lower boundary of the long length regime around $L = 10$.

6 Estimation of L^* and L_fis from the Bayesian posterior distribution

Using the results of our Bayesian inference, we estimated L^* and L_fis, parameters that emerged from our analysis of the repeat length dynamics at steady state. Under the informative prior, the subset of parameter values within the 95% HDR of the posterior lies along a narrow ridge of $\Delta\tau$ values (henceforth, *max posterior ridge*) with appreciable exponents $\tau_\epsilon, \tau_\kappa > 2$ and spanning multipliers $m = \{2.5, 4, 6.4\}$. This subset of realistic parameter combinations is localized at $\Delta\tau = 0.5$, rather than at specific values of the parameters τ_ϵ and τ_κ (at least within grid with step size of 0.1 for $\tau_\epsilon, \tau_\kappa$). Using Equation S23, these parameter values suggest a transition from expansion- to contraction-biased dynamics occurs at $L^* = 22.8$ (this is extended to a range of $L \approx$ 20–30 for the 99.7% HDR). This occurs at intermediate lengths within the long length regime, well below the truncation point of the DRL (for the T2T genome assembly, $L_\mathrm{trunc} = 65$). Posterior values under the uninformed (uniform) prior suggest a similar values for $\Delta\tau$ and thus L^*, despite a larger spread in the 95% HDR; this suggests that the rough estimation of L^* does not require instability rate estimates at intermediate lengths (e.g., those estimated from popSTR data [1]) but can instead be informed by properties of the DRL (e.g., onset length for repeat instability, truncation length, etc.).

In addition to constraining L^*, our inference results can be used to estimate the range of probable values of L_fis, representing the transition from substitution-dominated fission to insertion-dominated fission. Under the informative

prior, the maximum posterior of $(m, \tau_\epsilon, \tau_\kappa) = (4, 3.1, 3.6)$ has a corresponding fission-transition at $L_{\text{fis}} = 18.9$; the 95% HDR of the posterior spans a range of $m = 2.5$–6.4 and $\tau_\epsilon = 2$–3.5, which corresponds to a tight range of $L_{\text{fis}} = 17.2$–20.3 (under the uninformed prior, $L_{\text{fis}} \in [17.2, \infty)$; the posterior lacks power to estimate this parameter). These estimates suggest that a transition in the mutational process driving repeat interruption occurs somewhere in the neighborhood of $L \sim 20$, below which fission is largely a consequence of substitutions, while repeats above this range of lengths are primarily broken up by non-motif insertions.

The intermediate values of L^* and L_{fis} (which sit between the long repeat regime boundary and the truncation length) suggest that, in addition to the separability of long repeat dynamics from the substitution-driven short repeat regime, most long repeats are also *mechanistically independent* from short repeats; the primary mutational mechanisms that alter repeat length may be categorically different from replication and repair pathways that generate substitutions in shorter sequences. In this sense, two distinct boundaries can be placed on the lengths of repetitive sequences, corresponding to up to three distinct regimes: repeats below roughly 10 nucleotides are primarily subject to random substitutions; repeats below $L = L_{\text{fis}} \sim 20$, which may experience expansion and contraction, are subject to substitution-based interruptions; repeats longer than L_{fis} primarily exhibit repeat instability-based length changes, evolving both dynamically and mechanistically independently from shorter-length repeats subject to substitution-based effects.

Supplementary References

1. Kristmundsdottir, S. *et al.* Sequence variants affecting the genome-wide rate of germline microsatellite mutations. en. *Nat Commun* **14,** 3855 (2023).
2. Wolfram Research, Inc. *Mathematica, Version 14.0,* Champaign, IL, (2024).
