# Supplementary material for: Inherent instability of simple DNA repeats shapes an evolutionarily stable distribution of repeat lengths
Source: Nat Commun. 2025 Dec 9;17:93. doi: 10.1038/s41467-025-66725-5 (PMC12769509; doi:10.1038/s41467-025-66725-5)
Supplement: Supplementary file 2 — Description of Additional Supplementary Files [file 41467_2025_66725_MOESM2_ESM.pdf]

Description of Additional Supplementary Files

Supplementary Data 1: DRLs for mammalian genomes analyzed in this study. Distribution of repeat tract lengths. Tract length in units (columns) according to motif label (rows), grouped by unit length and genome assembly identifier.

Supplementary Data 2: Length-dependent instability rates calculated in this study. Rates for each mutation type (individual sheets), according to motif (columns) and tri-unit context or tract length in number of units (rows).
