# Supplementary material for: Inherent instability of simple DNA repeats shapes an evolutionarily stable distribution of repeat lengths
Source: Nat Commun. 2025 Dec 9;17:93. doi: 10.1038/s41467-025-66725-5 (PMC12769509; doi:10.1038/s41467-025-66725-5)
Supplement: Supplementary file 5 — Reporting Summary [file 41467_2025_66725_MOESM5_ESM.pdf]

nature portfolio

Corresponding author(s): Shamil Sunyaev, Sergei Mirkin

Last updated by author(s): Nov 4, 2025

Reporting Summary

Nature Portfolio wishes to improve the reproducibility of the work that we publish. This form provides structure for consistency and transparency in reporting. For further information on Nature Portfolio policies, see our Editorial Policies and the Editorial Policy Checklist.

Statistics

For all statistical analyses, confirm that the following items are present in the figure legend, table legend, main text, or Methods section.

n/a	Confirmed	
☐	☒	The exact sample size (n) for each experimental group/condition, given as a discrete number and unit of measurement
☐	☒	A statement on whether measurements were taken from distinct samples or whether the same sample was measured repeatedly
☒	☐	The statistical test(s) used AND whether they are one- or two-sided<br>*Only common tests should be described solely by name; describe more complex techniques in the Methods section.*
☒	☐	A description of all covariates tested
☒	☐	A description of any assumptions or corrections, such as tests of normality and adjustment for multiple comparisons
☐	☒	A full description of the statistical parameters including central tendency (e.g. means) or other basic estimates (e.g. regression coefficient) AND variation (e.g. standard deviation) or associated estimates of uncertainty (e.g. confidence intervals)
☒	☐	For null hypothesis testing, the test statistic (e.g. F, t, r) with confidence intervals, effect sizes, degrees of freedom and P value noted<br>*Give P values as exact values whenever suitable.*
☐	☒	For Bayesian analysis, information on the choice of priors and Markov chain Monte Carlo settings
☐	☒	For hierarchical and complex designs, identification of the appropriate level for tests and full reporting of outcomes
☒	☐	Estimates of effect sizes (e.g. Cohen's d, Pearson's r), indicating how they were calculated

Our web collection on statistics for biologists contains articles on many of the points above.

Software and code

Policy information about availability of computer code

Data collection	No software was used for data collection.
Data analysis	Custom code was written to perform the analysis in the current study, and is available in a Github repository (https://github.com/ryanmcggg/repeat_distributions).

For manuscripts utilizing custom algorithms or software that are central to the research but not yet described in published literature, software must be made available to editors and reviewers. We strongly encourage code deposition in a community repository (e.g. GitHub). See the Nature Portfolio guidelines for submitting code & software for further information.

Data

Policy information about availability of data

All manuscripts must include a data availability statement. This statement should provide the following information, where applicable:

- Accession codes, unique identifiers, or web links for publicly available datasets
- A description of any restrictions on data availability
- For clinical datasets or third party data, please ensure that the statement adheres to our policy

The datasets analyzed during the current study are freely available from the NCBI (https://www.ncbi.nlm.nih.gov/datasets/genome/), the UCSC Genome Browser (https://genome.ucsc.edu), and other studies as cited83-90. Instructions for accessing specific datasets are further detailed in the code repository (see Code Availability). DRLs for mammalian genomes analyzed in this study are provided in Supplementary Data File 1. Length-dependent instability rates calculated in this study are provided in Supplementary Data File 2.

Research involving human participants, their data, or biological material

Policy information about studies with human participants or human data. See also policy information about sex, gender (identity/presentation), and sexual orientation and race, ethnicity and racism.

Reporting on sex and gender	This study did not involve the creation of any new datasets or involvement of human participants. All analyses were performed using publicly available data.
Reporting on race, ethnicity, or other socially relevant groupings	This study did not involve the creation of any new datasets or involvement of human participants. Analysis was performed on publicly available data.
Population characteristics	Our measurement of mutation rates constitutes a re-analysis of genetic data from the Icelandic population; however, our use of publicly available summary statistics required no involvement with the original study population. This study did not involve the creation of any new datasets or involvement of human participants. All analyses were performed using publicly available data.
Recruitment	This study did not involve the creation of any new datasets or involvement of human participants. All analyses were performed using publicly available data.
Ethics oversight	This study did not involve the creation of any new datasets or involvement of human participants. All analyses were performed using publicly available data.

Note that full information on the approval of the study protocol must also be provided in the manuscript.

Field-specific reporting

Please select the one below that is the best fit for your research. If you are not sure, read the appropriate sections before making your selection.

- [x] Life sciences
- [ ] Behavioural & social sciences
- [ ] Ecological, evolutionary & environmental sciences

For a reference copy of the document with all sections, see nature.com/documents/nr-reporting-summary-flat.pdf

Life sciences study design

All studies must disclose on these points even when the disclosure is negative.

Sample size	Sample size was determined by availability of public data (e.g. one human telomere to telomere reference sequence, 315 mammalian genome assemblies, de novo mutations from 10,912 parent-child trios, popSTR mutations from 6,084 parent-child trios). These sample sizes represent the total of all unrestricted data available at the time of the study. Counts within the DRL are naturally limited by the size of the human genome. Sizes of grids of inferred parameters were limited by computational efficiency.
Data exclusions	No data were excluded from the analyses.
Replication	Our findings originate from the analysis of the human and other genomes; each genome represents a natural experiment and as such, cannot be replicated. Concerning reproducibility, our deterministic models and analytic equations produce the same outcome every time by design. All source data is publicly available. Together with the available code, all findings are able to be reproduced and figures can be replicated precisely. Our analysis of reproducibility in stochastic computational models is shown in Supplementary Fig. 13e. (Slight differences after reproducing this figure are expected due to randomness.)
Randomization	Not applicable to this study; samples were not allocated into groups for statistical analysis.
Blinding	Not applicable to this study; samples were not allocated into groups for statistical analysis.

Reporting for specific materials, systems and methods

We require information from authors about some types of materials, experimental systems and methods used in many studies. Here, indicate whether each material, system or method listed is relevant to your study. If you are not sure if a list item applies to your research, read the appropriate section before selecting a response.

Materials & experimental systems

n/a	Involved in the study
☒	☐ Antibodies
☒	☐ Eukaryotic cell lines
☒	☐ Palaeontology and archaeology
☒	☐ Animals and other organisms
☒	☐ Clinical data
☒	☐ Dual use research of concern
☒	☐ Plants

Methods

n/a	Involved in the study
☒	☐ ChIP-seq
☒	☐ Flow cytometry
☒	☐ MRI-based neuroimaging

Plants

Seed stocks	*Report on the source of all seed stocks or other plant material used. If applicable, state the seed stock centre and catalogue number. If plant specimens were collected from the field, describe the collection location, date and sampling procedures.*
Novel plant genotypes	*Describe the methods by which all novel plant genotypes were produced. This includes those generated by transgenic approaches, gene editing, chemical/radiation-based mutagenesis and hybridization. For transgenic lines, describe the transformation method, the number of independent lines analyzed and the generation upon which experiments were performed. For gene-edited lines, describe the editor used, the endogenous sequence targeted for editing, the targeting guide RNA sequence (if applicable) and how the editor was applied.*
Authentication	*Describe any authentication procedures for each seed stock used or novel genotype generated. Describe any experiments used to assess the effect of a mutation and, where applicable, how potential secondary effects (e.g. second site T-DNA insertions, mosiacism, off-target gene editing) were examined.*
